# Genetic associations with lipoprotein subfraction measures differ by ethnicity in the multi-ethnic study of atherosclerosis (MESA)

**DOI:** 10.1007/s00439-017-1782-y

**Published:** 2017-03-28

**Authors:** Zhe Wang, Ani Manichukal, David C. Goff, Samia Mora, Jose M. Ordovas, Nicholas M. Pajewski, Wendy S. Post, Jerome I. Rotter, Michele M. Sale, Stephanie A. Santorico, David Siscovick, Michael Y. Tsai, Donna K. Arnett, Stephen Rich, Alexis C. Frazier-Wood

**Affiliations:** 1 Department of Epidemiology, Human Genetics and Environmental Health, School of Public Health, University of Texas Health Science Center at Houston, Houston, TX 77030 USA; 2 Department of Public Health Sciences, University of Virginia, Charlottesville, VA 22908 USA; 3 Center for Public Health Genomics, University of Virginia, Charlottesville, VA 22908 USA; 4 Department of Epidemiology, Colorado School of Public Health, Aurora, CO 80045 USA; 5 Divisions of Preventive Medicine and Cardiovascular Medicine Brigham and Women’s Hospital, Harvard Medical School, Boston, MA 02115 USA; 6 Nutrition and Genomics Laboratory, Jean Mayer-US Department of Agriculture Human Nutrition Research Center on Aging, Tufts University, Boston, MA 02111 USA; 7 The Department of Epidemiology and Population Genetics, Centro Nacional Investigación Cardiovasculares (CNIC), 28029 Madrid, Spain; 8 IMDEA Food, 28049 Madrid, Spain; 9 Division of Public Health Sciences, Department of Biostatistical Sciences, Wake Forest School of Medicine, Winston-Salem, NC 27157 USA; 10 Division of Cardiology, Department of Medicine, Johns Hopkins University School of Medicine, Baltimore, MD 21205 USA; 11 Department of Epidemiology, Johns Hopkins Bloomberg School of Public Health, Baltimore, MD 21205 USA; 12 Los Angeles Biomedical Research Institute and Department of Pediatrics, Institute for Translational Genomics and Population Sciences, Harbor-UCLA Medical Center, Torrance, CA 90502 USA; 13 Department of Mathematical and Statistical Sciences, Human Medical Genetics and Genomics Program, Department of Biostatistics & Informatics, University of Colorado Denver, Denver, CO 80204 USA; 14 Cardiovascular Health Research Unit, Department of Medicine and Epidemiology, University of Washington, Seattle, WA 98195 USA; 15 Department of Laboratory Medicine and Pathology, University of Minnesota, Minneapolis, MN 55455 USA; 16 College of Public Health, University of Kentucky, Lexington, KY 40508 USA; 17 Department of Pediatrics, USDA/ARS Children’s Nutrition Research Center, Baylor College of Medicine, Houston, TX 77030 USA; 18 The New York Academy of Medicine, New York, NY 10029 USA

## Abstract

A recent genome-wide association study associated 62 single nucleotide polymorphisms (SNPs) from 43 genomic loci, with fasting lipoprotein subfractions in European–Americans (EAs) at genome-wide levels of significance across three independent samples. Whether these associations are consistent across ethnicities with a non-European ancestry is unknown. We analyzed 15 lipoprotein subfraction measures, on 1677 African–Americans (AAs), 1450 Hispanic–Americans (HAs), and 775 Chinese–Americans (CHN) participating in the multi-ethnic study of atherosclerosis (MESA). Genome-wide data were obtained using the Affymetrix 6.0 and Illumina HumanOmni chips. Linear regression models between genetic variables and lipoprotein subfractions were adjusted for age, gender, body mass index, smoking, study center, and genetic ancestry (based on principal components), and additionally adjusted for Mexican/Non-Mexican status in HAs. A false discovery rate correction was applied separately within the results for each ethnicity to correct for multiple testing. Power calculations revealed that we did not have the power for SNP-based measures of association, so we analyzed phenotype-specific genetic risk scores (GRSs), constructed as in the original genome-wide analysis. We successfully replicated all 15 GRS–lipoprotein associations in 2527 EAs. Among the 15 significant GRS–lipoprotein associations in EAs, 11 were significant in AAs, 13 in HAs, and 1 in CHNs. Further analyses revealed that ethnicity differences could not be explained by differences in linkage disequilibrium, lipid lowering drugs, diabetes, or gender. Our study emphasizes the importance of ethnicity (here indexing genetic ancestry) in genetic risk for CVD and highlights the need to identify ethnicity-specific genetic variants associated with CVD risk.

**Electronic supplementary material:**

The online version of this article (doi:10.1007/s00439-017-1782-y) contains supplementary material, which is available to authorized users.

## Introduction

Lipoproteins comprise a heterogeneous spectrum of particles, where each of the major lipoprotein fractions (i.e., very low-density, intermediate-density, low-density, and high-density lipoproteins; VLDL, IDL, LDL, and HDL, respectively) can be further subdivided into small, medium, and large subfractions that differ in size, density, and lipid content (Berneis and Krauss 2002). Greater concentrations of small LDL and large VLDL particles, and lower concentrations of large HDL particles relative to the other subfractions, have been previously shown to be associated with an increase in the risk of cardiovascular disease (CVD) and insulin resistance (IR) states, including diabetes (Gray et al. 1997; Garvey et al. 2003; Festa et al. 2005; Goff et al. 2005; Mora et al. 2009). Despite this evidence, the clinical utility of these and other lipoprotein particle measurements over more commonly used lipid and demographic measures is still uncertain (Krauss 2010; Steffen et al. 2015). However, since lipoprotein subfraction distributions are modifiable through therapeutic agents, as well as lifestyle (diet and exercise), they, nonetheless, represent a potential target for interventions aimed at reducing risk for CVD and IR (Beard et al. 1996; Lemieux et al. 2002; Melenovsky et al. 2002; Mauger et al. 2003; Wood et al. 2006). The associations between lipoprotein size and disease risk, coupled with the possibility that lipoprotein size may become a target for clinical interventions, have led to increased efforts to understand the etiology of lipoprotein subfraction heterogeneity.

In the most recent large-scale genome-wide association study (GWAS) meta-analysis, 62 SNPs from 43 genomic loci associated at genome-wide levels of significance with 22 lipoprotein measures, including subfraction concentrations, fraction diameters, and fraction particle numbers in a Caucasian population. Thirty-seven of the loci were replicated in two independent samples (Chasman et al. 2009). However, generalization of results from this GWAS is complicated for several reasons. First, the discovery population was exclusively women, although the replication samples included both males and females. Second, there are known differences in genetic variants associated with lipoprotein subfraction sizes between ethnic groups, for example, a separate study suggested that variation in the hepatic lipase (LIPC) gene was associated with mean HDL diameter in European–Americans (EA), but not in Americans reported as being from Chinese, African or Hispanic ethnic groups (Frazier-Wood et al. 2013). Similarly, variants in the APOB gene region were significantly associated with mean VLDL diameter in EAs and Hispanic–Americans (HA), this association was not present in Chinese nor African–Americans (CHN, AA, respectively) (Frazier-Wood et al. 2013). Such ethnic heterogeneity in gene–lipoprotein associations may reflect four possibilities: (1) the identity of single nucleotide polymorphisms (SNP) associated with disease risk or biomarkers differs across ethnic groups because of population differences in LD patterns (Aouizerat et al. 2003; Goodarzi et al. 2003; Wung and Aouizerat 2003); (2) the directions of effects at such loci could be different across populations; (3) they may display magnitude inconsistencies; or (4) different environmental mediators may be present in the various groups. Together these suggest that extra care is needed when applying genetic risk models to non-Caucasians (Carlson et al. 2013).

Our aim is to investigate whether associations of previously validated genetic variants and lipoprotein subfractions can be generalized to other ethnic populations, using data from the Multi-Ethnic Study of Atherosclerosis (MESA). Specifically, after replicating the associations of variants identified as associating with lipoprotein measures at genome-wide significance in Caucasians, and validated in two independent Caucasian samples (Chasman et al. 2009) in the EA population of MESA, we sought to examine these associations in the African-, Hispanic-, and Chinese populations of MESA, using both single SNP and summed genetic risk scores (GRS).

## Materials and methods

### Study population

The MESA study population consisted of 6814 men and women, ages 45–84 years, who self-identified as non-Hispanic White (European Americans, EA), AA, HA, or CHN, and were recruited from six regions in the United States from 2000 to 2002. The communities included Forsyth County, North Carolina; Northern Manhattan and the Bronx, New York; Baltimore City and Baltimore County, Maryland; St Paul, Minnesota; Chicago, Illinois; and Los Angeles County, California. MESA was designed to study the prevalence, risk factors, and progression of subclinical CVD in a multi-ethnic cohort. A detailed description of the study design and methods has been published previously (Bild et al. 2002). All participants were free of clinically apparent cardiovascular disease. Clinical characteristics, including anthropometric and blood-pressure measurements, were taken at the study clinics, where a fasting blood sample was also drawn. Questionnaires were administered at clinics to collect self-reported demographic data, including age, gender, race, country of origin, and information on lifestyle attributes and medical history. All participants gave informed consent, and the protocol was approved by the institutional review board of each of the study centers.

### Biochemical measurements

Twelve hour fasting blood was drawn and serum, and EDTA-anticoagulant tubes were collected and stored at −70° using a standardized protocol (Bild et al. 2002). Lipoprotein subfractions, including the measurements of VLDL, LDL, and HDL diameter and subclass concentrations, were determined by nuclear magnetic resonance (NMR) spectroscopy by LipoScience (North Carolina, now LabCorp). This technique simultaneously quantifies the average particle size of lipoprotein fractions and concentration (“number”) of lipoprotein subfraction particles expressed each as an average particle diameter (in nanometers; nm) or as lipoprotein particle concentration (in mol/l), respectively (Otvos 2001; Kuller et al. 2002; Mora et al. 2007; Mackey et al. 2012). NMR detects the signal emitted by lipoprotein methyl-group protons when in the field of a magnet charged at 400 MHz. Particle concentrations of lipoproteins of different sizes were estimated from the deconvoluted NMR signals. Weighted-average lipoprotein particle sizes are derived from the sum of the diameter of each subclass multiplied by its relative mass percentage based on the amplitude of its methyl NMR signal. NMR groups IDL as a subclass of LDL (Jeyarajah et al. 2006). In the original GWAS, Chasman and colleagues examined 15 lipoprotein subfraction measures determined by NMR together with LDL-C, HDL-C determined by NMR and enzymatic assay, triglycerides, ApoA1 and ApoB. Of these 15 lipoprotein subfractions, all 15 lipoprotein measures were assayed in MESA and included in the current analyses.

### DNA collection protocol and genotyping

Although our analyses focus on candidate loci, we utilized data from the MESA GWAS protocol. Genomic DNA was isolated from whole blood for genetic analysis on all participants. The quality of DNA was assessed by single SNP ABI TaqMan. Genome-wide data were obtained using the Affymetrix 6.0 chip and additional deeper sequencing using the Illumina HumanOmni chip. Exclusion criteria include heterozygosity >53% and individual-level genotyping call rate <95%. SNPs with call rate <95%, and monomorphic SNPs were removed. We further filtered on race/ethnic-specific Hardy–Weinberg equilibrium *p* value >1 × 10^−5^. IMPUTE version 2.2.2 was used to perform imputation for the MESA SHARe participants (chromosomes 1–22) using 1000 genomes as the reference panel. Imputed SNPs were filtered on observed/expected variance >0.5 derived from the MACH software (Li et al. 2010). Relationship inference was performed using the KING software (Manichaikul et al. 2010) to identify first- and second-degree relatives, and an unrelated set of individuals was identified for genome-wide association data collection. All pairs of individuals with KING-inferred kinship coefficient >0.2 were identified as first-degree relatives. Based on this criterion, all first-degree relatives were grouped into families, and an unrelated subset of individuals was constructed by choosing at most one individual from each family.

### Statistics

All analyses were performed using SAS 9.4 (SAS Institute, Cary, NC, USA).

#### Sample characteristics

To examine whether the ethnic groups differed by age, we performed *t* tests, with the exception of ethnicity differences by gender that were examined using a 2-degree of freedom (*df*) Chi-square (*χ* ^2^) test. These tests revealed significant age differences by ethnicity (Table 1). Since both age and gender were associated with lipoprotein measures in our sample (data not shown), analyses examining ethnicity differences in lipoprotein measures (also presented in Table 1) were examined using regression models with mean lipoprotein measure as the outcome and age and gender as covariates.

**Table 1:** Means (±standard deviation), or percentages for demographic characteristics, and fasting lipid traits and lipoprotein diameters for the MESA study participants

	EAs^a^	AAs^b^	HAs^c^	CHNs^d^	Ethnicity differences^e^
*N*	2527	1677	1450	775	–
Gender (% male)	47.7	46.0	48.4	49.2	
Age (years)	62.7 (10.2)	62.2 (10.1)	61.4 (10.3)	62.4 (10.4)	2, 4, 6
Lipid lowering drugs (% taking)	18.3	16.0	13.4	14.1	–
Hypertension (% yes)	38.6	59.2	41.9	37.7	–
Triglycerides (mg/dl)	133.05 (90.10)	104.90 (69.91)	158.19 (101.75)	143.00 (85.67)	–
VLDL large (nmol/L)	5.34 (6.71)	3.17 (4.17)	7.03 (7.47)	4.64 (6.81)	1, 2, 3, 4, 5, 6
VLDL medium (nmol/L)	28.78 (21.41)	21.07 (17.11)	32.80 (21.39)	39.57 (26.90)	1, 2, 3, 4, 5, 6
VLDL small (nmol/L)	34.12 (19.72)	29.21 (18.60)	35.95 (20.41)	41.49 (20.66)	1, 2, 3, 4, 5, 6
VLDL total (nmol/L)	68.24 (36.41)	53.45 (32.48)	75.78 (37.08)	85.71 (38.25)	1, 2, 3, 4, 5, 6
IDL total (nmol/L)	131.02 (101.76)	116.12 (90.11)	138.60 (107.29)	112.61 (89.12)	1, 2, 3, 4, 6
LDL large (nmol/L)	607.23 (259.65)	603.29 (251.01)	549.06 (270.45)	537.02 (239.85)	2, 3, 4, 5
LDL small (nmol/L)	505.88 (373.85)	497.24 (372.01)	631.88 (400.40)	552.09 (374.26)	2, 3, 4, 5, 6
LDL total (nmol/L)	1244.12 (324.50)	1216.67 (359.23)	1319.51 (347.43)	1201.73 (311.64)	1, 2, 3, 4, 6
HDL large (µmol/L)	6.09 (3.60)	6.41 (3.61)	5.35 (3.03)	6.03 (3.09)	1, 2, 4, 5, 6
HDL medium (µmol/L)	14.99 (7.44)	11.86 (6.05)	13.39 (6.50)	10.92 (5.74)	1, 2, 3, 4, 5, 6
HDL small (µmol/L)	13.95 (5.96)	15.24 (5.42)	14.47 (5.46)	16.69 (5.57)	1, 2, 3, 4, 5, 6
HDL total (µmol/L)	35.03 (7.06)	33.50 (6.51)	33.20 (6.27)	33.63 (5.85)	1, 2, 3
VLDL diameter (nm)	49.11 (8.39)	47.29 (6.72)	50.91 (8.54)	46.34 (7.18)	1, 2, 3, 4, 5, 6
LDL diameter (nm)	20.81 (0.56)	20.79 (0.51)	20.64 (0.58)	20.61 (0.51)	2, 3, 4, 5
HDL diameter (nm)	9.25 (0.46)	9.30 (0.49)	9.16 (0.43)	9.29 (0.41)	1, 2, 3, 4, 6
LDL-cholesterol (mg/dl)	117.10 (30.28)	116.47 (33.17)	119.82 (32.87)	115.10 (28.80)	–
HDL-cholesterol (mg/dl)	52.40 (15.76)	52.34 (15.16)	47.51 (13.04)	49.33 (12.41)	–

^a^EA: European American

^b^AA: African American

^c^HA–Hispanic American

^d^CA: Chinese American

^e^Significant (*p* < 0.05) differences between: 1: EAs and AAs; 2: EAs and HAs; 3: EAs and CHNs; 4: AAs and HAs; 5: AAs and CHNs; and 6: HAs and CHNs participants in *t* tests (age), *χ* ^2^ te**s**t (gender), and regression model (lipoprotein measures) of ethnicity difference

Log-transformations or square-root transformations were used when lipoprotein measures exhibited (residual) deviations from normality. Concentrations of large VLDL and large HDL particles were log-transformed, concentrations of medium and small VLDL, and medium HDL, as well as total VLDL, IDL, and HDL particles were square root transformed. We inspected graphical methods including histograms, and interpreted statistics including skewness and kurtosis to formally test for normality after transformation.

Since ethnicity was self-reported, within the full MESA cohort, we stratified by ethnic group and eliminated those individuals with top principal components (PCs) of ancestry >3.5 SD from the mean within any ethnic group. Details about the race/ethnic-specific PCs of ancestry used for adjustment have been reported previously (Manichaikul et al. 2012). Briefly, we constructed subsets of genotyped SNPs after LD-pruning for each ethnic group. Regions of long-range LD were removed, and local LD structures were thinned using a pairwise *R* ^2^ of no more than 0.2 in a 100 SNP window, moving at 25 SNP blocks. SMARTPCA (Patterson et al. 2006; Price et al. 2006) within EIGENSTRAT was used to compute PCs. Based on previous examination in MESA, we used 3 PCs for EAs, 1 PC for AAs, 3 PCs for HAs, and 1 PC for CHNs and finally eliminated 22 individuals from EAs, 3 from AAs and 1 from HAs due to being more than 3.5 SD from the mean PC value within that ethnic group. All models controlled for age, gender, BMI, current smoking status, study center, and principal components of ancestry (PCs, PC1–PC4) as fixed effects. In HAs, models were additionally adjusted for Mexican/Non-Mexican status.

#### SNP–lipoprotein associations

To examine genetic associations, we first fitted linear model with lipoprotein subfraction measures as the outcome and individual SNPs as the predictor. Initially, we explored the association between individual SNPs as in the original GWAS (Chasman et al. 2009) with lipoprotein measures in EAs. As in the original GWAS, the minor allele in EAs was coded as 1 and major allele was coded as 0 in an additive model. Subsequently, the associations were conducted in the other ethnic populations of the MESA. As the original associations reported by Chasman et al. (2009) may have reported an SNP–lipoprotein association, where the SNP was not directly associated with the outcome, but rather in linkage disequilibrium (LD) with the causal SNP, and because LD structures differ between ethnic groups (The International HapMap Consortium 2003), where SNP–lipoprotein associations were not significant at an FDR corrected *Q* < 0.05, we examined associations using proxy SNPs identified using SNAP (Johnson et al. 2008) which were in LD at *R* ^2^ > 0.8 in EAs, but not in the other ethnicities, and so may not be indexed by the identified SNP. An FDR correction was used within each ethnic group on *p* values for all associations including those with proxy SNPs.

#### GRS–phenotype associations

Unweighted GRSs were constructed and presented as our main results as they may be more robust against errors in estimating the effect sizes arising from limited sample sizes, and may be more suitable for reducing increased estimates of association due to population heterogeneity, population substructure, and “winner’s curse” (Dudbridge 2013). When constructing the GRSs, the constituent genotypes were rescored to have the same effect on the phenotype, based on the direction reported in the original GWAS (Chasman et al. 2009). The major allele was kept as the coded allele when the regression coefficient in the original GWAS (Chasman et al. 2009) was negative for a given SNP–lipoprotein association; the minor allele remained the coded allele when the original regression coefficient was positive. Whether the direction of our reported regression coefficient is consistent with the direction of the original coefficient is shown in S1 Table. This resulted in genotypes being scored both ways (with the minor allele and the major allele as the coded allele) based on the particular phenotype. Subsequently, we created GRS by summing the alleles of either the SNP from associations reported in EAs, or from a proxy SNP, where the original SNP–lipoprotein association was null (*Q* > 0.05), but the proxy SNP was significantly associated with lipoprotein subfraction measures at *Q* < 0.05. An FDR correction was used within each ethnic group, and corrected *q* values were reported (Benjamini and Hochberg 1995; Storey and Tibshirani 2003).

#### Power analysis for genetic associations

We conducted power analyses to estimate our power to detect associations in the MESA cohort for SNPs associated with lipoprotein measures in EAs from the original GWAS (Chasman et al. 2009). Power analyses were conducted using Quanto v 1.2.4 (Gauderman and Morrison 2001; Gauderman 2002) with Type I error 0.05 (thus our power calculation indicated ‘nominal significance’, i.e., not corrected for the number of independent tests). The observed effect sizes (*β*) from the SNP–lipoprotein associations in the Caucasian population of the original GWAS (Chasman et al. 2009), as well as the observed minor allele frequency (MAF) for each of the ethnic populations of MESA were used to calculate our power to replicate SNP–lipoprotein associations of the same or larger effect size across ethnicities. Our power estimates showed that we had limited (<80%) power for majority single SNP–phenotype associations (S1 Table); therefore, we present individual SNP–phenotype associations as supplementary data and focus on results using GRSs for each lipoprotein phenotype, since GRSs may have increased power over single SNP associations (Chasman et al. 2009; Frazier-Wood et al. 2014). Power analyses for GRS–lipoprotein associations were conducted in G*Power version 3.1.9.2 (Faul et al. 2007, 2009) specifying *R* ^2^ for the GRS–lipoprotein association in the fasting subsample in the original GWAS (Chasman et al. 2009) and Bonferroni corrected Type I error 0.05/15 = 0.0033, in a linear regression model, including ten predictors for European-, African-, and Chinese-ethnic group individuals: GRS, age, gender, BMI, current smoking status, study centers, and PC1–PC4. We specified 11 predictors in HAs as we included country of origin (Mexicans or non-Mexican Hispanics) in the model.

## Results

### Demographic characteristics in MESA

General characteristics of the MESA study participants are summarized in Table 1. There were no gender differences by ethnicity, but there were small (although statistically significant, *P* < 0.05) age differences (Table 1). For the 15 lipoprotein subfractions examined, we saw significant ethnic differences between all populations in seven subfractions: large VLDL, medium VLDL, small VLDL and VLDL total particles; medium and small HDL particles; and mean VLDL diameter (*P* < 0.05; Table 1). For the other subfractions, as whole we still observed a general pattern of differences between the ethnic groups, although in a few cases, these did not reach significance.

### Replication of previous genetic findings in EAs

Some SNPs associated with more than one lipoprotein measure in the original GWAS. We included the 62 unique SNPs which associated with at least one of the 15 phenotypes available in our data, leading to a total of 131 SNP–lipoprotein association models, to be run in the Caucasian population. One SNP (rs3129882) in the original GWAS is not available in our genotype data. We conducted a proxy SNP search using SNAP (Johnson et al. 2008), however, that query SNP is not in 1000 Genomes Pilot1 and there were no matching proxy SNPs found. Therefore, it was excluded from analysis. Initially, we replicated 59 out of 131 (45%) of the SNP–lipoprotein associations at an FDR-adjusted *Q* < 0.05 (S1 Table; Chasman et al. 2009). Only the association of rs7706174 and large LDL particle differed in direction as the previous GWAS, and was significant (*Q* < 0.05). However, which variant was the coded allele in the previous GWAS was not reported for this locus. It is possible that the different direction of this association might, therefore, be due to differences in allele coding between the original study and ours. Power analyses suggested that some of the non-replication may have been due to statistical power, as our power was less 80% in ~60% of SNP–lipoprotein associations in MESA EAs (S2 Table). However, using GRSs, we had over 99% power to detect the GRS–lipoprotein associations detected in the original GWAS in MESA EAs (S3 Table; Chasman et al. 2009). All GRS–lipoprotein associations were significant at an FDR *Q* < 0.05 (Table 2), suggesting that overall, the same genetic effects were operating on lipoprotein subfractions in our EA population as in the previous GWAS (Chasman et al. 2009; Table 2).

**Table 2:** Characteristics (mean and standard deviation) of Genetic Risk Scores (GRS) and associations^a^ of GRSs with lipoprotein phenotypes

Lipoprotein fractions		European–Americans (*n* = 2506)				African–Americans (*n* = 1610)				Hispanic–Americans (*n* = 1448)				Chinese–Americans (*n* = 775)			
		*β*	*P*	*Q*	GRS mean (SD)	*β*	*P*	*Q*	GRS mean (SD)	*β*	*P*	*Q*	GRS mean (SD)	*β*	*P*	*Q*	GRS mean (SD)
VLDL																	
Large	**0.10**		**7.14** × **10** ^**−8**^	**1.9** × **10** ^**−7**^	**7.84 (1.32)**	0.03	0.15	0.17	8.39 (1.37)	**0.09**	**4.27** × **10** ^**−5**^	**6.37** × **10** ^**−5**^	**7.79 (1.36)**	2.28 × 10^−5^	1.00	1.00	7.40 (1.22)
Medium	**0.13**		**3.4** × **10** ^**−9**^	**7.3** × **10** ^**−9**^	**12.14 (1.70)**	**0.09**	**0.002**	**0.003**	**11.73 (1.62)**	**0.16**	**4.3** × **10** ^**−9**^	**1.63** × **10** ^**−8**^	**11.97 (1.72)**	0.01	0.80	1.00	11.90 (1.57)
Small	**0.07**		**1.24** × **10** ^**−4**^	**1.43** × **10** ^**−4**^	**15.10 (1.96)**	0.03	0.22	0.23	14.51 (1.97)	**0.09**	**1.06** × **10** ^**−4**^	**1.33** × **10** ^**−4**^	**15.03 (1.98)**	−0.002	0.94	1.00	15.61 (1.77)
Total	**0.11**		**2.64** × **10** ^**−6**^	**3.60** × **10** ^**−6**^	**14.68 (1.88)**	0.06	0.04	0.05*	14.33 (1.82)	**0.18**	**2** × **10** ^**−10**^	**1.2** × **10** ^**−9**^	**13.99 (1.94)**	−0.01	0.81	1.00	13.81 (1.82)
IDL																	
Total	**0.21**		**4.38** × **10** ^**−4**^	**4.69** × **10** ^**−4**^	**4.30 (1.44)**	**0.15**	**0.03**	**0.04**	**4.58 (1.48)**	0.17	0.04	0.05*	4.45 (1.42)	**0.39**	**3.52** × **10** ^**−4**^	**0.005**	**4.68 (1.39)**
LDL																	
Large	**16.64**		**<1** × **10** ^**−10**^	**<1** × **10** ^**−10**^	**16.91 (2.06)**	**15.36**	**2.86** × **10** ^**−7**^	**2.15** × **10** ^**−6**^	**18.25 (2.00)**	**17.58**	**6.06** × **10** ^**−8**^	**1.30** × **10** ^**−7**^	**16.89 (2.12)**	5.79	0.19	0.48	17.38 (1.88)
Small	**20.10**		**<1** × **10** ^**−10**^	**<1** × **10** ^**−10**^	**11.71 (2.28)**	**11.82**	**0.003**	**0.005**	**11.36 (2.21)**	**22.91**	**1.41** × **10** ^**−7**^	**2.64** × **10** ^**−7**^	**11.10 (2.29)**	3.09	0.66	1.00	11.39 (1.85)
Total	**0.26**		**5.70** × **10** ^**−7**^	**8.55** × **10** ^**−7**^	**13.29 (1.74)**	**0.24**	**5.94** × **10** ^**−4**^	**0.001**	**11.81 (1.78)**	**0.29**	**4.67** × **10** ^**−5**^	**6.37** × **10** ^**−5**^	**13.34 (1.86)**	0.02	0.87	1.00	13.95 (1.53)
HDL																	
Large	**0.07**		**<1** × **10** ^**−10**^	**<1** × **10** ^**−10**^	**8.33 (1.91)**	**0.05**	**3** × **10** ^**−10**^	**4.1** × **10** ^**−9**^	**8.51 (1.76)**	**0.06**	**<1** × **10** ^**−10**^	**<1** × **10** ^**−10**^	**7.86 (1.92)**	0.03	0.02	0.08*	6.77 (1.65)
Medium	**0.06**		**3.27** × **10** ^**−5**^	**4.09** × **10** ^**−5**^	**3.69 (1.33)**	**0.10**	**5.52** × **10** ^**−6**^	**2.76** × **10** ^**−5**^	**3.26 (0.99)**	0.02	0.18	0.18	4.45 (1.37)	0.05	0.07	0.26	4.30 (1.11)
Small	**0.58**		**<1** × **10** ^**−10**^	**<1** × **10** ^**−10**^	**4.07 (1.40)**	**0.33**	**6.58** × **10** ^**−4**^	**0.001**	**5.04 (1.40)**	**0.51**	**4.27** × **10** ^**−8**^	**1.07** × **10** ^**−7**^	**4.33 (1.50)**	0.35	0.01	0.08*	5.22 (1.32)
Total	**0.26**		**6.50** × **10** ^**−4**^	**6.50** × **10** ^**−4**^	**5.12 (1.60)**	**0.24**	**0.03**	**0.04**	**5.37 (1.40)**	**0.25**	**0.01**	**0.01**	**4.89 (1.54)**	0.25	0.13	0.41	4.63 (1.16)
Diameters																	
VLDL	**0.07**		**1.60** × **10** ^**−8**^	**3.00** × **10** ^**−8**^	**1.47 (0.98)**	0.004	0.75	0.75	1.17 (0.95)	**0.07**	**1.23** × **10** ^**−6**^	**2.04** × **10** ^**−6**^	**1.60 (1.09)**	0.02	0.36	0.78	1.55 (0.93)
LDL	**0.04**		**<1** × **10** ^**−10**^	**2** × **10** ^**−10**^	**12.01 (1.75)**	**0.02**	**3.92** × **10** ^**−4**^	**0.001**	**11.68 (1.82)**	**0.06**	**<1** × **10** ^**−10**^	**<1** × **10** ^**−10**^	**12.76 (1.89)**	0.004	0.68	1.00	12.95 (1.64)
HDL	**0.04**		**<1** × **10** ^**−10**^	**<1** × **10** ^**−10**^	**9.94 (1.78)**	**0.03**	**1.08** × **10** ^**−5**^	**4.05** × **10** ^**−5**^	**11.22 (1.68)**	**0.03**	**2.70** × **10** ^**−8**^	**8.10** × **10** ^**−8**^	**10.53 (1.76)**	0.004	0.64	1.00	10.15 (1.68)

Significant results in bold

* Nominally significant results

^a^Models are adjusted for age, gender, BMI, current smoking status, study center, and principal components (PC1–PC4) in all ethnicities; models are additionally adjusted for Mexican/Non-Mexican status in Hispanic Americans

### Examination of associations in other ethnicities

*SNP*–*lipoprotein associations* 15 out of 131 original SNP–lipoprotein associations were significant after multiple testing correction (FDR *Q* < 0.05) in AAs, 37 out of 131 were significant in HAs, and 1 out of 131 were significant in CHNs (S1 Table). For those SNP–lipoprotein associations that were not significant after accounting for multiple testing, we conducted the associations using proxy SNPs (S4–7 Table). From these proxy SNPs, we saw only two additional proxy SNP–lipoprotein associations in CHNs (rs676210—small VLDL particle and rs673548—small VLDL particle; original SNP rs6754295 (both *Q* < 0.05; S6 Table). As no other significant SNP–lipoprotein association was reported with proxy SNPs, we did not include proxy SNPs in the next step of GRS calculations.

*GRS*–*lipoprotein associations* We had over 95% power to detect GRS–lipoprotein associations in AAs, HAs, and CHNs based on the effect size reported in the GWAS meta-analysis (S3 Table). For AAs, 11 out of 15 GRS–lipoprotein associations were significant at an FDR *Q* < 0.05 (Table 2). Specifically, the GRS associations with most VLDL subfraction measures, including concentrations of large, small, and total VLDL particles, as well as mean VLDL diameters, were not significant in AAs. Almost all of (13 out of 15) GRS–lipoprotein associations were significant at an FDR *Q* < 0.05 in HAs except for the association with total IDL particle concentration and medium HDL particle concentration. Only 1 out of 15 GRS–lipoprotein associations were significant at an FDR *Q* < 0.05 in CHNs, which is the GRS association with concentration of total IDL particles (Table 2).

### Secondary analyses

We considered the potential modifying effects of lipid lowering drugs, diabetes, and gender in the association of genetic variants with lipoprotein subfraction measures. In secondary analyses, we additionally excluded MESA participants on lipid lowering medications and those with type 2 diabetes (Malave et al. 2012) at baseline to minimize environmental influences on lipoprotein subfraction measures. Although we observed moderately attenuated associations with smaller coefficients, the overall pattern of results for GRS–lipoprotein associations was not substantially different from the primary analyses (S8 Table). In addition, as the original GWAS included only female participants (Chasman et al. 2009), we conducted gender-stratified analyses for the GRS–lipoprotein associations (S9 Table). only a very small number of cases were the GRS–lipoprotein associations significant for one gender, but not the other (S9 Table). In which case, we tested for a statistical interaction between gender and GRS in the associations. We did not observe any significant interactions with gender after multiple testing correction, justifying our inclusion of both genders in the main analyses, but as these analyses are limited in power, we do not draw main conclusions from the lack of an interaction (S9 Table).

## Discussion

This study replicated SNP–lipoprotein and GRS–lipoprotein associations previously validated in EAs (Chasman et al. 2009), and further investigated whether those associations could be generalized to Hispanic-, African-, and Chinese-ethnic groups living in the US. We provide evidence that there is heterogeneity in the genetic basis of the lipoprotein subfractions across ethnic groups: among the 15 significant associations between GRSs and lipoprotein subfraction phenotypes in EAs, 11 showed significant associations in AAs, 13 in HAs, and only 1 in CHNs.

In our initial analyses, we replicated 45% of the original SNP–lipoprotein associations in EAs (at an FDR-adjusted significance level), and even less in AAs, HAs, and CHNs. Numerous reasons could account for the failure to replicate remaining SNPs; for example, the limited statistical power we had in replicating and examining individual SNP–lipoprotein associations, or sample differences between our cohort and those of the discovery GWAS (notably ours was of mixed gender). We did tentatively explore the possibility that for significant associations in EAs, the lack of replication in the other ethnic groups arose from the putatively causal SNP not being genotyped in the original discovery efforts, possibly creating differential associations due to variable LD patterns across the ethnic groups (Tam and Consortium 2003; Frazier-Wood and Rich 2015). However, we did not observe many proxy SNP–lipoprotein associations that reached an FDR-adjusted significance level, with the exception of the associations of two proxy SNPs (rs676210 and rs673548) with concentration of small VLDL particles in CHNs. Although 131 association tests may be considered modest in the GWAS era, our sample size may also be considered modest for genetic analysis too. Given this, even with an FDR correction for multiple testing, we cannot rule out the possibility that these two associations are false positives Therefore, the lack of significant proxy SNP associations suggests that we cannot provide evidence that the heterogeneity in genetic effects for lipoprotein subfraction measures is due to differences in LD structure across ethnicities.

Despite the fact that only some of the previous SNP–lipoprotein associations were significantly replicated in our data, we did have more than 95% power to detect GRS–lipoprotein associations. All GRS–lipoprotein associations we examined were significant in EAs, validating the previous findings by (Chasman et al. 2009). Most were significant in HAs; however, the GRS association with most VLDL subfraction measures, including concentrations of large VLDL and small VLDL particles, VLDL total particles, and VLDL diameters, was not significant in AAs and CHNs. In addition, none of the examined GRS–lipoprotein associations were significant in CHNs except for the GRS association with concentration of total IDL particles. The differing GRS–lipoprotein associations we observed, given that we considered the role of ethnicity-specific LD structure in our GRS creations, suggests that overall, the genetic effects on lipoprotein subfractions were most similar between HAs and EAs. Some of the genetic effects on lipoprotein subfractions in AAs did mirror those of EAs, but to a lesser extent than did those of the HIS group. AA associations also had some similarities to those seen in the HIS group, although again, this was to a small extent. CHNs largely differed from the ones in EAs, with only one GRS–lipoprotein association, from those GRSs derived in EA populations being significant in the CHN group. Although we must emphasize the small size of the CHN population in this study, our findings are, nonetheless, consistent with the literature about genetic structures and differentiation across ethnicities, which found distinct and non-overlapping clustering of the Caucasian, African–American and Chinese samples in the US (Mountain and Cavalli-Sforza 1997; Jorde and Wooding 2004; Shriver et al. 2004), while Hispanics, who represent a recently admixed group between Native Americans, Caucasians, and Africans, did not form a distinct subgroup (Hanis et al. 1991; González Burchard et al. 2005), and, therefore, are expected to be more genetically similar to EAs than CHNs and AAs.

There is currently a dearth of studies that have investigated ethnic heterogeneity in genetic associations with lipoprotein subfraction measures. To the best of our knowledge, only one such study has been conducted: they found that the variants in the *APOB* gene region were associated with mean VLDL diameter in EAs and HAs, but not in AAs and CHNs, while variation in the *LIPC* gene was suggestively associated with mean HDL diameters only in EAs (Frazier-Wood et al. 2013). Thirty-seven of the lipoprotein-associated loci included that the current analyses are also robustly associated with lipids in Caucasian populations (Teslovich et al. 2010; Global Lipids Genetics 2013); we find mixed evidence on whether these variants also associate with lipids in other ancestry populations (Keebler et al. 2009; Lanktree et al. 2009; Teslovich et al. 2010; Chang et al. 2011; Dumitrescu et al. 2011; Musunuru et al. 2012; Bryant et al. 2013; Wu et al. 2013; Below et al. 2016). Some of these studies were able to replicate the same associations (or at minimally identify directionally consistent effects) identified in EAs in non-European ancestry populations (Keebler et al. 2009; Lanktree et al. 2009; Teslovich et al. 2010; Bryant et al. 2013); for example, the associations between lipids and variants in *APOB* as well as *PPP1R3B* genes that were identified in EAs were well replicated in HISs and AAs (Teslovich et al. 2010; Bryant et al. 2013). However, several multi-ethnic analyses of lipid-associated loci had reported that the specific-associated variants often differed across ethnicities (Teslovich et al. 2010; Dumitrescu et al. 2011; Musunuru et al. 2012; Bryant et al. 2013; Wu et al. 2013). Interestingly, several lipids genes identified from large European ancestry GWAS, including *APOC1*-*APOE*, *MAFB*, *LIPG,* etc, were observed to have differential association among East Asians (Teslovich et al. 2010), which are consistent with our results suggesting that CHNs largely differed from the ones in EAs. A trans-ethnic fine-mapping study of lipid loci identified different variants in the *PCSK9* and *APOA5* genes that influenced LDL-C levels in Europeans and AAs (Wu et al. 2013). This emphasizes the need for more research on the transferability of SNPs across ancestry groups, and we hope that the data here eventually contribute to a broader body of evidence on this issue than is currently available.

The strengths of this study include the large multi-ethnic population, and it is one of the first studies focusing on ethnicity difference of genetic associations with a full profile of lipoprotein subfraction measures. In addition, eliminating those individuals with top principal components (PCs) of ancestry >3.5 SD from the mean within each ethnic group minimizes the misclassification of self-report ancestry. However, there are some limitations that must be considered upon interpreting our results. First, we were underpowered to fully replicate all the previous individual SNP–phenotype associations: our EA group was smaller than in the original EA GWAS, the other ethnic groups were smaller still, and our total number of participants may have been slightly smaller than necessary due to the use of the KING software to remove close relatives over an approach based on graph theory (Staples et al. 2013). This sample size issue is particularly pertinent to the CHN group. However, our GRS analysis was well-powered and showed significant GRS–phenotype associations in EAs. In addition, even though the imputation quality was well controlled in EAs with an observed/expected variance greater than 0.5, 3 out of 61 SNPs in CHNs and 1 out of 61 SNPs in AAs had low imputation quality (Supplementary Table 10) and may contribute to failure of replication in these two ethnicity groups. However, given the GRS-based results to which these SNP contribute very little variance, it is not likely that very few SNPs of lowered imputation quality adversely affect our results.

In addition, as suggested by the gender-specific effect on genetic basis of lipid profiles and metabolic syndrome (McCarthy et al. 2003; Wung and Aouizerat 2003; Aulchenko et al. 2009), there could be increased heterogeneity due to gender effects in our population when compared to the original GWAS (Chasman et al. 2009), since the original GWAS was conducted in a sample of exclusively women. We attempted to explore the GRS–lipoprotein associations stratified by gender. However, due to the reduced sample size in stratified analysis, we could not draw any firm conclusions and encourage further research into this. Finally, we were unable to explore reasons for the ethnic heterogeneity in SNP–lipoprotein associations observed and the roles of non-genetic differences between the populations, gene–gene interactions, and gene–environment interactions would form important directions for future research (Ma et al. 2012). Our study emphasizes the need for future studies for associations with lipoprotein subfraction measures in non-Caucasian populations, which may identify novel SNPs. Such work may help to better understand ethnic differences in CVD risk, and may elucidate different pathways to risk between the groups.

In conclusion, associations between GRS and lipoprotein subfraction measures largely differed by self-reported ethnicity except between HAs and EAs. The observed differences may be due to unidentified genetic influences on lipoproteins in ethnic groups with differing genetic ancestries, or other factors, such as gene–environment interactions. Our study highlights the need for future research which investigates ethnicity-specific SNPs associated with CVD risk, leading to the possibility of ethnicity- (and potentially ancestry-) stratified prediction and precision treatment.

## Electronic supplementary material

Below is the link to the electronic supplementary material.

Supplementary material 1 (DOCX 318 kb)
