# Supplementary material for: Genetic associations with lipoprotein subfraction measures differ by ethnicity in the multi-ethnic study of atherosclerosis (MESA)
Source: Hum Genet. 2017 Mar 28;136(6):715–26. doi: 10.1007/s00439-017-1782-y (PMC5429342; doi:10.1007/s00439-017-1782-y)
Supplement: Supplementary file 1 — Supplementary material 1 (DOCX 318 kb) [file 439_2017_1782_MOESM1_ESM.docx]

**S 1 Table** SNP-phenotype associations^a^ validated as associating with European Americans in a previous GWAS, conducted among the four ethnic groups in MESA

| Chr | Genes | SNPs | European-American | | | African-American | | | Hispanic-American | | | Chinese-American | | |  |
| --- | --- | --- | --- | --- | --- | --- | --- | --- | --- | --- | --- | --- | --- | --- | --- |
|  |  |  | beta | +/-^b^ | P | beta | +/-^b^ | P | beta | +/-^b^ | P | beta | +/-^b^ | P |  |
| VLDL Large | |  |  |  |  |  |  |  |  |  |  |  |  |  |  |
| 2 | GCKR | rs1260326 | 0.18 | + | **7.48*10^-7**^** | 0.12 | + | 0.04^*^ | 0.16 | + | **7.63*10^-4**^** | 0.16 | + | 0.04^*^ |  |
| 6 | **BTNL2** | **rs3129882^c^** |  |  |  |  |  |  |  |  |  |  |  |  |  |
| 8 | LPL | rs1059611 | -0.18 | + | **0.002^**^** | -0.08 | + | 0.13 | -0.11 | + | 0.12 | 0.17 | _ | 0.12 |  |
| 11 | APOA1-A5 | rs3135506 | 0.11 | + | 0.14 | 0.26 | + | 0.008^*^ | 0.32 | + | **2.13*10^-6**^** | -4.36 | _ | 0.82 |  |
| 19 | APOC1-APOE | rs439401 | -0.09 | + | 0.07 | -0.01 | + | 0.83 | 0.002 | _ | 0.97 | -0.05 | + | 0.54 |  |
| 5 | TIMD4\|HAVCR1 | rs1354163 | -0.04 | + | 0.42 | 0.10 | _ | 0.26 | -0.19 | + | 0.03^*^ | 0.10 | _ | 0.84 |  |
| 16 | LCAT | rs1109166 | 0.009 | + | 0.85 | -0.03 | _ | 0.50 | -0.02 | _ | 0.75 | -0.12 | _ | 0.34 |  |
| 20 | MAFB | rs1076759 | -0.05 | + | 0.44 | 0.002 | _ | 0.97 | 0.01 | _ | 0.83 | 0.09 | _ | 0.28 |  |
| VLDL Medium | |  |  |  |  |  |  |  |  |  |  |  |  |  |  |
| 1 | ANGPTL3 | rs10889353 | -0.14 | + | **0.02^**^** | -0.11 | + | 0.09 | -0.15 | + | 0.03^*^ | -0.18 | + | 0.20 |  |
| 2 | APOB | rs673548 | -0.22 | + | **0.001^**^** | -0.07 | + | 0.38 | -0.07 | + | 0.34 | -0.02 | + | 0.86 |  |
| 2 | GCKR | rs1260326 | 0.08 | + | 0.15 | 0.20 | + | 0.02^*^ | 0.17 | + | 0.03^*^ | 0.07 | + | 0.54 |  |
| 7 | MLXIPL | rs2240466 | -0.32 | + | **3.26*10^-4**^** | -0.44 | + | **0.007^**^** | -0.36 | + | 0.01^*^ | 0.01 | _ | 0.97 |  |
| 8 | LPL | rs1059611 | -0.43 | + | **4.66*10^-7**^** | -0.15 | + | 0.07 | -0.50 | + | **1.93*10^-5**^** | -0.07 | + | 0.71 |  |
| 11 | APOA1-A5 | rs3135506 | 0.36 | + | **0.003^**^** | 0.37 | + | 0.01^*^ | 0.52 | + | **1.35*10^-6**^** | -8.52 | _ | 0.79 |  |
| 10 | JMJD1C | rs12768534 | 0.06 | + | 0.29 | 0.04 | + | 0.52 | -0.05 | _ | 0.48 | -0.09 | _ | 0.49 |  |
| 5 | TIMD4\|HAVCR1 | rs1354163 | -0.06 | + | 0.48 | 0.20 | _ | 0.15 | -0.45 | + | **7.11*10^-4**^** | 0.51 | _ | 0.52 |  |
| 16 | LCAT | rs255052 | -0.03 | _ | 0.70 | -0.06 | _ | 0.45 | -0.01 | _ | 0.91 | 0.001 | + | 0.99 |  |
| 20 | MAFB | rs2865892 | 0.02 | _ | 0.85 | -0.04 | + | 0.65 | -0.16 | + | 0.08 | 0.05 | _ | 0.68 |  |
| VLDL Small | |  |  |  |  |  |  |  |  |  |  |  |  |  |  |
| 1 | PCSK9 | rs11591147 | -0.02 | + | 0.95 | -0.05 | + | 0.94 | -0.15 | + | 0.76 | 9.82 | _ | 0.05 |  |
| 1 | ANGPTL3 | rs1167998 | -0.06 | + | 0.27 | -0.04 | + | 0.57 | -0.15 | + | 0.02^*^ | -0.06 | + | 0.54 |  |
| 1 | CELSR2/PSRC1/SPRT1 | rs646776 | -0.07 | + | 0.25 | -0.006 | + | 0.92 | -0.15 | + | 0.04^*^ | -0.05 | + | 0.78 |  |
| 2 | APOB | rs6754295 | -0.29 | + | **9.65*10^-7**^** | -0.11 | + | 0.09 | -0.30 | + | **2.84*10^-5**^** | -0.32 | + | **5.55*10^-4*^** |  |
| 8 | LPL | rs328 | -0.29 | + | **3.13*10^-4**^** | -0.04 | + | 0.76 | -0.16 | + | 0.19 | 0.10 | _ | 0.44 |  |
| 9 | ABO | rs507666 | 0.19 | + | **0.002^**^** | 0.13 | + | 0.19 | 0.16 | + | 0.09 | 0.21 | + | 0.04^*^ |  |
| 11 | APOA1-A5 | rs3135506 | 0.01 | + | 0.92 | 0.17 | + | 0.21 | 0.15 | + | 0.14 | 9.53 | + | 0.68 |  |
| 16 | CETP | rs1800775 | -0.02 | + | 0.67 | 0.09 | _ | 0.19 | 0.14 | _ | 0.04^*^ | 0.24 | _ | 0.006^*^ |  |
| 19 | LDLR | rs6511720 | -0.15 | + | 0.12 | -0.04 |  | 0.76 | 0.06 | _ | 0.71 | -1.37 |  | 0.11 |  |
| 19 | APOC1-APOE | rs439401 | 0.08 | _ | 0.22 | -0.03 | + | 0.75 | -0.11 | + | 0.20 | -0.02 | + | 0.87 |  |
| 5 | TIMD4\|HAVCR1 | rs1363232 | -0.06 | + | 0.27 | -0.07 | + | 0.28 | -0.10 | + | 0.18 | -0.17 | + | 0.14 |  |
| 16 | LCAT | rs255052 | -0.12 | _ | 0.10 | 7.04*10^-4^ | + | 0.99 | 0.04 | + | 0.64 | 0.08 | + | 0.55 |  |
| 20 | MAFB | rs6102085 | -0.02 | _ | 0.71 | 0.05 | + | 0.44 | -0.02 | _ | 0.81 | 0.16 | + | 0.05 |  |
| VLDL Total | |  |  |  |  |  |  |  |  |  |  |  |  |  |  |
| 1 | PCSK9 | rs11591147 | 0.36 | _ | 0.34 | -0.12 | + | 0.88 | -0.13 | + | 0.82 | 17.46 | _ | 0.006^*^ |  |
| 1 | ANGPTL3 | rs10889353 | -0.16 | + | **0.02**** | -0.19 | + | 0.01^*^ | -0.22 | + | **0.005^**^** | -0.21 | + | 0.10 |  |
| 2 | APOB | rs673548 | -0.34 | + | **3.88*10^-6**^** | -0.14 | + | 0.11 | -0.25 | + | **0.004^**^** | 0.27 | _ | 0.02^*^ |  |
| 2 | GCKR | rs780094 | 0.05 | + | 0.45 | 0.23 | + | 0.01^*^ | 0.21 | + | 0.01^*^ | 0.09 | + | 0.42 |  |
| 8 | LPL | rs328 | -0.56 | + | **9.8*10^-9**^** | -0.12 | + | 0.42 | -0.61 | + | **1.54*10^-5**^** | 0.02 | _ | 0.90 |  |
| 11 | APOA1-A5 | rs3135506 | 0.30 | + | **0.02^**^** | 0.42 | + | 0.01^*^ | 0.56 | + | **2.98*10^-6**^** | -0.48 | _ | 0.99 |  |
| 16 | CETP | rs1800775 | -0.09 | + | 0.15 | -0.05 | + | 0.55 | -0.01 | + | 0.89 | 0.05 | _ | 0.67 |  |
| 19 | APOC1-APOE | rs439401 | 0.006 | _ | 0.94 | 0.05 | _ | 0.68 | -0.09 | + | 0.39 | -0.08 | + | 0.52 |  |
| 5 | TIMD4\|HAVCR1 | rs1354163 | -0.12 | + | 0.17 | 0.17 | _ | 0.28 | -0.51 | + | **5.22*10^-4**^** | 0.33 | _ | 0.65 |  |
| 16 | LCAT | rs255052 | -0.08 | _ | 0.36 | -0.04 | _ | 0.68 | 0.025 | + | 0.81 | 0.04 | + | 0.84 |  |
| 20 | MAFB | rs2865892 | -0.03 | + | 0.78 | 0.02 | _ | 0.82 | -0.19 | + | 0.06 | 0.09 | _ | 0.46 |  |
| 10 | JMJD1C | rs7923609 | -0.04 | _ | 0.54 | -0.09 |  | 0.23 | -0.03 | + | 0.73 | -0.004 | + | 0.97 |  |
| IDL total | |  |  |  |  |  |  |  |  |  |  |  |  |  |  |
| 2 | GCKR | rs780094 | 0.15 | + | 0.24 | 0.20 | + | 0.28 | 0.11 | + | 0.56 | 0.21 | + | 0.34 |  |
| 15 | LIPC | rs1532085 | 0.37 | + | **0.005^**^** | 0.15 | + | 0.30 | 0.50 | + | **0.005^**^** | 0.35 | + | 0.12 |  |
| 16 | CETP | rs1800775 | -0.37 | + | **0.005^**^** | -0.23 | + | 0.15 | -0.36 | + | 0.05 | -0.36 | + | 0.12 |  |
| 5 | *TIMD4\|HAVCR1* | rs1363232 | -0.12 | + | 0.34 | -0.10 | + | 0.49 | -0.05 | + | 0.80 | -0.72 | + | 0.02^*^ |  |
| 16 | *LCAT* | rs255049 | -0.05 | _ | 0.77 | 0.11 | + | 0.47 | -0.29 | _ | 0.15 | 0.80 | + | 0.02^*^ |  |
| LDL Large | |  |  |  |  |  |  |  |  |  |  |  |  |  |  |
| 1 | PCSK9 | rs11591147 | -70.40 | + | 0.10 | 42.52 | _ | 0.66 | -33.00 | + | 0.65 | -1700.27 | + | 0.02^*^ |  |
| 2 | APOB | rs1713222 | -23.80 | + | **0.01^**^** | -15.85 | + | 0.11 | -38.72 | + | **0.007^**^** | 81.43 | _ | 0.52 |  |
| 5 | HMGCR | rs5744680 | 11.30 | + | 0.11 | 2.76 | + | 0.83 | -8.60 | _ | 0.38 | 15.50 | + | 0.19 |  |
| 8 | LPL | rs2083637 | 27.30 | + | **2.92*10^-4**^** | -3.10 | _ | 0.77 | 23.86 | + | 0.04^*^ | 2.86 | + | 0.85 |  |
| 11 | FADS1-3 | rs1535 | -26.20 | + | **3.49*10^-4**^** | -27.69 | + | 0.03^*^ | -28.53 | + | **0.006^**^** | -9.33 | + | 0.45 |  |
| 15 | LIPC | rs1800588 | 30.60 | + | **2.75*10^-4**^** | 29.12 | + | **0.001^**^** | 59.47 | + | **8.5*10^-9**^** | 23.09 | + | 0.08 |  |
| 16 | CETP | rs1864163 | -35.80 | + | **6.17*10^-5**^** | -22.78 | + | 0.04^*^ | -50.34 | + | **3.58*10^-5**^** | -52.76 | + | 0.005^*^ |  |
| 18 | LIPG | rs8090363 | -8.26 | + | 0.24 | -1.98 | + | 0.83 | -13.52 | + | 0.20 | 3.23 | _ | 0.81 |  |
| 19 | LDLR | rs6511720 | -19.10 | + | 0.17 | -19.52 | + | 0.31 | 13.63 | _ | 0.54 | -222.30 | + | 0.07 |  |
| 19 | APOC1-APOE | rs4803750 | -44.10 | + | **0.001^**^** | 10.37 | _ | 0.48 | -6.98 | + | 0.62 | 23.70 | _ | 0.30 |  |
| 20 | PLTP | rs4810479 | -19.90 | + | **0.01^**^** | -18.53 | + | 0.04^*^ | -12.45 | + | 0.21 | 12.85 | _ | 0.30 |  |
| 5 | **TIMD4\|HAVCR1** | rs7706174 | 23.90 | _ | **0.004^**^** | -4.29 | + | 0.64 | 3.51 | _ | 0.78 | 3.34 | _ | 0.82 |  |
| 20 | MAFB | rs6102085 | 9.32 | + | 0.19 | 1.46 | + | 0.88 | -0.96 | _ | 0.92 | -3.19 | _ | 0.79 |  |
| LDL Small | |  |  |  |  |  |  |  |  |  |  |  |  |  |  |
| 1 | CELSR2/PSRC1/SPRT1 | rs646776 | -3.57 | + | 0.77 | -15.70 | + | 0.23 | -42.09 | + | **0.009^**^** | -49.84 | + | 0.17 |  |
| 2 | GCKR | rs780094 | 23.30 | + | **0.02^**^** | -3.61 | _ | 0.82 | -2.70 | _ | 0.86 | 12.92 | + | 0.48 |  |
| 8 | LPL | rs2083637 | -50.90 | + | **1.38*10^-6**^** | 7.09 | _ | 0.65 | -55.28 | + | **0.001^**^** | -5.89 | + | 0.79 |  |
| 8 | TRIB1 | rs6982636 | -31.00 | + | **0.001^**^** | -4.11 |  | 0.75 | -15.33 | + | 0.28 | -9.87 | + | 0.60 |  |
| 11 | APOA1-A5 | rs3135506 | 61.80 | + | **0.003^**^** | 50.81 | + | 0.08 | 54.33 | + | 0.01^*^ | -3212.93 | _ | 0.52 |  |
| 15 | LIPC | rs1800588 | -1.74 | + | 0.88 | -37.89 | + | **0.005^**^** | -56.96 | + | **1.53*10^-4**^** | -0.72 | + | 0.97 |  |
| 16 | CETP | rs1800775 | -54.20 | + | **5.70*10^-8**^** | -24.85 | + | 0.07 | -54.18 | + | **2.96*10^-4**^** | -3.30 | + | 0.86 |  |
| 19 | APOC1-APOE | rs769449 | 46.60 | + | **0.005^**^** | 72.40 | + | 0.10 | -24.60 | _ | 0.47 | 69.81 | + | 0.06 |  |
| 20 | PLTP | rs6065906 | 35.00 | + | **0.004^**^** | 25.20 | + | 0.14 | 13.89 | + | 0.52 | 44.71 | + | 0.44 |  |
| 5 | TIMD4\|HAVCR1 | rs7706174 | -15.70 | _ | 0.17 | 20.90 | + | 0.11 | -8.87 | _ | 0.63 | -16.62 | _ | 0.46 |  |
| 16 | LCAT | rs7200210 | -14.70 | _ | 0.51 | 7.15 | + | 0.59 | -1.70 | _ | 0.95 | -38.86 | _ | 0.70 |  |
| 20 | MAFB | rs2865892 | 10.30 | _ | 0.48 | 23.51 | _ | 0.19 | -23.79 | + | 0.19 | 55.21 | _ | 0.007^*^ |  |
| LDL Total | |  |  |  |  |  |  |  |  |  |  |  |  |  |  |
| 1 | PCSK9 | rs11591147 | 0.11 | _ | 0.89 | 2.14 | _ | 0.29 | -1.33 | + | 0.32 | -31.53 | + | 0.02^*^ |  |
| 1 | CELSR2/PSRC1/SPRT1 | rs646776 | -0.29 | + | 0.06 | -0.67 | + | **3.61*10^-4**^** | -1.11 | + | **3.26*10^-8**^** | -0.58 | + | 0.21 |  |
| 2 | APOB | rs312985 | -0.58 | + | **1.93*10^-4**^** | -0.75 | + | **4.67*10^-5**^** | -0.69 | + | **0.003^**^** | -0.11 | + | 0.94 |  |
| 2 | GCKR | rs780094 | 0.10 | + | 0.44 | -0.03 | _ | 0.90 | -0.17 | + | 0.38 | 0.17 | _ | 0.46 |  |
| 8 | TRIB1 | rs6982636 | -0.28 | + | 0.03 | -0.04 | + | 0.82 | -0.27 | + | 0.13 | -0.09 | + | 0.70 |  |
| 11 | APOA1-A5 | rs3135506 | 0.30 | + | 0.26 | 0.19 | + | 0.65 | 0.18 | + | 0.51 | -41.69 | _ | 0.51 |  |
| 16 | CETP | rs1800775 | -0.36 | + | **0.006^**^** | -0.19 | + | 0.34 | -0.08 | + | 0.69 | 0.08 | _ | 0.76 |  |
| 19 | LDLR | rs6511720 | -0.38 | + | 0.14 | -0.16 | + | 0.69 | -0.71 | + | 0.08 | -1.42 | + | 0.54 |  |
| 19 | APOC1-APOE | rs769449 | 0.74 | + | **8.15*10^-4**^** | 1.74 | + | **0.006^**^** | -0.32 | _ | 0.46 | 0.87 | + | 0.06 |  |
| 5 | TIMD4\|HAVCR1 | rs1363232 | 0.14 | _ | 0.32 | 0.05 | _ | 0.80 | 0.01 | + | 0.95 | -0.22 | _ | 0.48 |  |
| 20 | MAFB | rs2865892 | -0.01 | + | 0.94 | 0.18 | _ | 0.50 | -0.35 | + | 0.12 | 0.76 | _ | 0.003^*^ |  |
| HDL Large | |  |  |  |  |  |  |  |  |  |  |  |  |  |  |
| 8 | LPL | rs331 | 0.09 | + | **1.36*10^-6**^** | 0.03 | + | 0.22 | 0.03 | + | 0.15 | -0.02 | _ | 0.52 |  |
| 11 | FADS1-3 | rs174546 | -0.09 | + | **8.37*10^-7**^** | -0.07 | + | 0.05 | -0.08 | + | **2.33*10^-4**^** | -0.06 | + | 0.03^*^ |  |
| 12 | CCDC92/DNAH10/ZNF664 | rs7307277 | 0.04 | + | **0.01^**^** | 0.06 | + | 0.01^*^ | 0.03 | + | 0.19 | -0.01 | _ | 0.79 |  |
| 15 | LIPC | rs1800588 | 0.11 | + | **5.33*10^-8**^** | 0.11 | + | **2.46*10^-6**^** | 0.17 | + | **<1*10^-10**^** | 0.08 | + | 0.01^*^ |  |
| 16 | CETP | rs1800775 | 0.12 | + | **<1*10^-10**^** | 0.08 | + | **4.06*10^-4**^** | 0.11 | + | **7.49*10^-8**^** | 0.01 | + | 0.61 |  |
| 18 | LIPG | rs4939883 | -0.04 | + | 0.08 | -0.02 | + | 0.39 | -0.02 | + | 0.53 | -0.02 | + | 0.60 |  |
| 20 | PLTP | rs6065904 | -0.10 | + | **3.79*10^-6**^** | -0.11 | + | **1.28*10^-4**^** | -0.05 | + | 0.05 | -0.03 | + | 0.34 |  |
| 5 | TIMD4\|HAVCR1 | rs6873053 | 0.003 | + | 0.94 | -0.21 | _ | 0.02^*^ | -0.008 | + | 0.91 | -0.30 | + | 0.53 |  |
| 16 | LCAT | rs2271293 | 0.02 | + | 0.42 | 0.05 | + | 0.24 | 0.02 | + | 0.46 | -0.06 | _ | 0.56 |  |
| 20 | MAFB | rs2865879 | 0.02 | + | 0.15 | -0.02 | _ | 0.52 | 0.02 | + | 0.44 | 0.01 | + | 0.63 |  |
| HDL Medium | |  |  |  |  |  |  |  |  |  |  |  |  |  |  |
| 1 | APOA2 | rs4073054 | -0.06 | + | **0.03^*^** | -0.12 | + | **0.001^**^** | -0.04 | + | 0.29 | -0.16 | + | 0.03^*^ |  |
| 11 | FADS1-3 | rs174537 | 0.06 | + | **0.02^**^** | 0.05 | + | 0.37 | 0.02 | + | 0.48 | 0.03 | + | 0.53 |  |
| 19 | APOC1-APOE | rs405509 | -0.09 | + | **7.26*10^-4**^** | -0.15 | + | **1.57*10^-4**^** | -0.01 | + | 0.72 | -0.07 | + | 0.24 |  |
| 5 | *TIMD4\|HAVCR1* | rs4704810 | 0.02 | + | 0.59 | 0.006 | + | 0.92 | 0.02 | + | 0.57 | -0.01 | _ | 0.87 |  |
| HDL Small | |  |  |  |  |  |  |  |  |  |  |  |  |  |  |
| 2 | GCKR | rs780094 | 0.28 | + | 0.08 | 0.25 | + | 0.30 | 0.59 | **+** | **0.005^**^** | 0.73 | + | 0.009^*^ |  |
| 11 | APOA1-A5 | rs518181 | 0.62 | + | **2.34*10^-4**^** | -0.04 | _ | 0.88 | -0.10 | _ | 0.63 | 0.60 | + | 0.07 |  |
| 15 | LIPC | rs1800588 | -0.56 | + | **0.004^**^** | -0.33 | + | 0.11 | -0.53 | + | 0.01^*^ | 0.19 | _ | 0.55 |  |
| 20 | PLTP | rs4810479 | 1.39 | + | **<1*10^-10**^** | 1.15 | + | **2.21*10^-8**^** | 1.21 | + | **2*10^-9**^** | 0.51 | + | 0.07 |  |
| 16 | *LCAT* | rs1109166 | 0.02 | + | 0.91 | -0.16 | _ | 0.43 | 0.34 | + | 0.16 | -0.56 | + | 0.24 |  |
| HDL total | |  |  |  |  |  |  |  |  |  |  |  |  |  |  |
| 2 | GCKR | rs1260326 | 0.22 | + | 0.21 | 0.49 | + | 0.12 | 0.55 | + | 0.02^*^ | 0.45 | + | 0.11 |  |
| 11 | APOA1-A5 | rs518181 | 0.19 | + | 0.29 | 0.15 | + | 0.61 | -0.09 | _ | 0.70 | 0.34 | + | 0.31 |  |
| 16 | CETP | rs7499892 | -0.81 | + | **0.003^**^** | -0.99 | + | **1.77*10^-4**^** | -0.77 | + | **0.006^**^** | -0.56 | + | 0.16 |  |
| 20 | PLTP | rs6065906 | 0.55 | + | **0.01^**^** | 0.46 | + | 0.12 | 1.03 | + | **0.002^**^** | -0.40 | + | 0.64 |  |
| 16 | *LCAT* | rs1109166 | 0.14 | + | 0.56 | 0.06 | + | 0.78 | 0.40 | + | 0.12 | 0.002 | + | 0.99 |  |
| 20 | *MAFB* | rs6016408 | -0.12 | _ | 0.54 | -0.15 | _ | 0.55 | -0.73 | _ | **0.006^**^** | -0.56 | _ | 0.28 |  |
| 11 | SBF2 | rs7938647 | 0.35 | + | 0.07 | -0.99 | _ | 0.03^*^ | 0.18 |  | 0.53 | -3.99 | _ | 0.22 |  |
| VLDL diameter | |  |  |  |  |  |  |  |  |  |  |  |  |  |  |
| 2 | APOB | rs676210 | 0.07 | + | **9.11*10^-4**^** | 0.03 | + | 0.25 | 0.06 | + | 0.01^*^ | 0.06 | + | 0.05 |  |
| 2 | GCKR | rs1260326 | 0.07 | + | **7.74*10^-6**^** | -0.007 | _ | 0.77 | 0.09 | + | **7.78*10^-5**^** | 0.08 | + | 0.005^*^ |  |
| 8 | intergenic, PPP1R3B | rs983309 | 0.04 | + | 0.19 | 0.004 | + | 0.83 | 0.05 | + | 0.05 | 0.18 | + | 0.15 |  |
| LDL diameter | |  |  |  |  |  |  |  |  |  |  |  |  |  |  |
| 2 | GCKR | rs780094 | -0.03 | + | 0.03^*^ | -0.002 | + | 0.92 | -0.03 | + | 0.24 | -0.008 | + | 0.76 |  |
| 8 | LPL | rs2083637 | 0.08 | + | **9.20*10^-7**^** | 0.005 | + | 0.82 | 0.08 | + | **0.002^**^** | 0.01 | + | 0.74 |  |
| 11 | APOA1-A5 | rs3135506 | -0.12 | + | **5.83*10^-5**^** | -0.08 | + | 0.05 | -0.13 | + | **2.95*10^-5**^** | 4.13 | _ | 0.55 |  |
| 15 | LIPC | rs1800588 | 0.02 | + | 0.22 | 0.06 | + | **0.001^**^** | 0.11 | + | **4.39*10^-7**^** | 0.03 | + | 0.30 |  |
| 16 | CETP | rs1800775 | 0.09 | + | **1.10*10^-9**^** | 0.03 | + | 0.11 | 0.12 | + | **6.01*10^-8**^** | 0.02 | + | 0.44 |  |
| 18 | LIPG | rs4939883 | -0.007 | + | 0.70 | -0.01 | + | 0.48 | -0.02 | + | 0.39 | -0.04 | + | 0.20 |  |
| 20 | PLTP | rs6065906 | -0.06 | + | **9.64*10^-4**^** | -0.06 | + | 0.01^*^ | -0.04 | + | 0.16 | -0.07 | + | 0.36 |  |
| 5 | TIMD4\|HAVCR1 | rs7706174 | 0.03 | _ | 0.09 | -0.03 | + | 0.13 | 0.01 | _ | 0.62 | 0.02 | _ | 0.55 |  |
| 16 | LCAT | rs7200210 | 0.02 | _ | 0.47 | 0.005 | _ | 0.78 | 0.02 | _ | 0.56 | 0.19 | + | 0.16 |  |
| 20 | MAFB | rs2865892 | -0.008 | _ | 0.72 | -0.006 | _ | 0.80 | 0.04 |  | 0.10 | -0.06 | _ | 0.04^*^ |  |
| HDL diameter | |  |  |  |  |  |  |  |  |  |  |  |  |  |  |
| 2 | GCKR | rs780094 | -0.02 | + | 0.08 | 0.006 | _ | 0.75 | 0.004 | _ | 0.80 | -1.54*10^-4^ | + | 0.99 |  |
| 8 | LPL | rs331 | 0.04 | + | **0.001^**^** | 0.02 | + | 0.35 | 0.01 | + | 0.43 | -0.03 | _ | 0.28 |  |
| 11 | FADS1-3 | rs1535 | -0.05 | + | **4.51*10^-6**^** | -0.04 | + | 0.09 | -0.06 | + | **2.54*10^-4**^** | -0.06 | + | 0.002^*^ |  |
| 15 | LIPC | rs1800588 | 0.07 | + | **1.09*10^-6**^** | 0.07 | + | **9.11*10^-6**^** | 0.10 | + | **3*10^-10**^** | 0.03 | + | 0.18 |  |
| 16 | CETP | rs1800775 | 0.07 | + | **2.0*10^-10**^** | 0.05 | + | **0.001^**^** | 0.04 | + | **0.004^**^** | -0.01 | _ | 0.57 |  |
| 18 | LIPG | rs4939883 | -0.02 | + | 0.26 | -0.01 | + | 0.41 | -0.005 | + | 0.80 | 0.05 | _ | 0.03^*^ |  |
| 20 | PLTP | rs6065906 | -0.09 | + | **3.0*10^-10**^** | -0.05 | + | 0.01^*^ | -0.03 | + | 0.20 | -0.12 | + | 0.06 |  |
| 16 | LCAT | rs4986970 | -0.09 | + | **0.02^**^** | 0.04 | _ | 0.72 | -0.01 | + | 0.92 | 1.91*10^-4^ | _ | 0.99 |  |
| 20 | MAFB | rs2865892 | -0.003 | _ | 0.85 | -0.03 | _ | 0.13 | 0.003 | + | 0.87 | -0.01 | _ | 0.53 |  |

“^a^ models are adjusted for age, gender, BMI, current smoking status, study center and Principle Components (PC1-PC4) in all ethnicities; additionally models are adjusted for Mexican/Non-Mexican status in Hispanic Americans.

^b^ +/- : + indicates observed betas are in the same direction as the betas in the original GWAS; - indicates observed betas are in the opposite direction comparing to the betas in the original GWAS

**^c^ rs3129882 annotated to BTNL2 gene in the GWAS conducted by Chasman and colleagues is not available in MESA’s dataset, as well as not in 1000 Genome Pilot 1. Therefore it is not included in further analysis.**

^*^nominally significant p<0.05; ^**^**False discovery rate adjusted significant, in** **bold** “

**S 2** **Table** Power calculations to replicate the individual SNP-phenotype associations previously reported in European-Americans, in four ethnic groups in MESA

| Marker | Chr | Gene | Position | EA^a^ (n=2506) | | AA^b^ (n=1610) | | HA^c^ (n=1448) | | CHN^d^ (n=775) | |
| --- | --- | --- | --- | --- | --- | --- | --- | --- | --- | --- | --- |
|  |  |  |  | MAF | Power | CAF^e^ | Power | CAF^e^ | Power | CAF^e^ | Power |
| VLDL large |  |  |  |  |  |  |  |  |  |  |  |
| rs1260326 | 2 | GCKR | 27730940 | 0.44 | 60% | 0.16 | 56% | 0.33 | 34% | 0.50 | 27% |
| rs1059611 | 8 | LPL | 19824563 | 0.12 | 38% | 0.17 | 64% | 0.09 | 20% | 0.12 | 18% |
| rs3135506 | 11 | APOA1-A5 | 116662407 | 0.06 | 38% | 0.06 | 53% | 0.12 | 34% | 1.6*10^-4^ | 5% |
| rs439401 | 19 | APOC1-APOE | 45414451 | 0.38 | 27% | 0.21 | 30% | 0.46 | 18% | 0.56 | 14% |
| rs1354163 | 5 | TIMD4\|HAVCR1 | 156420703 | 0.15 | 12% | 0.05 | 10% | 0.07 | 8% | 0.01 | 6% |
| rs1109166 | 16 | LCAT | 67977382 | 0.17 | 13% | 0.61 | 22% | 0.26 | 11% | 0.10 | 8% |
| rs1076759 | 20 | MAFB | 39353684 | 0.09 | 11% | 0.18 | 17% | 0.17 | 10% | 0.20 | 9% |
| VLDL Medium | |  |  |  |  |  |  |  |  |  |  |
| rs10889353 | 1 | ANGPTL3 | 63118196 | 0.31 | 63% | 0.37 | 67% | 0.34 | 46% | 0.20 | 19% |
| rs673548 | 2 | APOB | 21237544 | 0.21 | 91% | 0.22 | 92% | 0.26 | 78% | 0.73 | 42% |
| rs1260326 | 2 | GCKR | 27730940 | 0.44 | 84% | 0.16 | 62% | 0.33 | 60% | 0.50 | 32% |
| rs2240466 | 7 | MLXIPL | 72856269 | 0.11 | 55% | 0.04 | 29% | 0.07 | 29% | 0.08 | 17% |
| rs1059611 | 8 | LPL | 19824563 | 0.12 | 93% | 0.17 | 98% | 0.09 | 67% | 0.12 | 40% |
| rs3135506 | 11 | APOA1-A5 | 116662407 | 0.06 | 99% | 0.06 | 99% | 0.12 | 99% | 1.6*10^-4^ | 6% |
| rs12768534 | 10 | JMJD1C | 65340897 | 0.45 | 45% | 0.45 | 45% | 0.40 | 30% | 0.31 | 15% |
| rs1354163 | 5 | TIMD4\|HAVCR1 | 156420703 | 0.15 | 18% | 0.05 | 12% | 0.07 | 11% | 0.01 | 6% |
| rs255052 | 16 | LCAT | 68024995 | 0.14 | 18% | 0.21 | 21% | 0.16 | 14% | 0.09 | 8% |
| rs2865892 | 20 | MAFB | 39403896 | 0.12 | 17% | 0.14 | 18% | 0.19 | 16% | 0.28 | 11% |
| VLDL Small |  |  |  |  |  |  |  |  |  |  |  |
| rs11591147 | 1 | PCSK9 | 55505647 | 0.01 | 73% | 0.01 | 61% | 0.01 | 50% | 0.001 | 10% |
| rs1167998 | 1 | ANGPTL3 | 62931632 | 0.32 | 81% | 0.64 | 71% | 0.41 | 62% | 0.25 | 34% |
| rs646776 | 1 | CELSR2/PSRC1/SPRT1 | 109818530 | 0.21 | 92% | 0.36 | 92% | 0.24 | 76% | 0.06 | 24% |
| rs6754295 | 2 | APOB | 21206183 | 0.24 | 99% | 0.28 | >99% | 0.27 | >99% | 0.71 | 97% |
| rs328 | 8 | LPL | 19819724 | 0.11 | 80% | 0.06 | 48% | 0.08 | 47% | 0.12 | 39% |
| rs507666 | 9 | ABO | 136149399 | 0.20 | 86% | 0.11 | 57% | 0.15 | 56% | 0.20 | 42% |
| rs3135506 | 11 | APOA1-A5 | 116662407 | 0.06 | 97% | 0.06 | 91% | 0.12 | 97% | 0.00 | 7% |
| rs1800775 | 16 | CETP | 56995236 | 0.49 | 96% | 0.57 | 89% | 0.51 | 81% | 0.53 | 57% |
| rs6511720 | 19 | LDLR | 11202306 | 0.11 | 76% | 0.11 | 64% | 0.08 | 44% | 0.01 | 11% |
| rs439401 | 19 | APOC1-APOE | 45414451 | 0.38 | 79% | 0.21 | 54% | 0.46 | 58% | 0.56 | 37% |
| rs1363232 | 5 | TIMD4\|HAVCR1 | 156383422 | 0.34 | 34% | 0.45 | 29% | 0.25 | 21% | 0.16 | 13% |
| rs255052 | 16 | LCAT | 68024995 | 0.14 | 39% | 0.21 | 39% | 0.16 | 28% | 0.09 | 15% |
| rs6102085 | 20 | MAFB | 39281629 | 0.36 | 40% | 0.28 | 29% | 0.40 | 27% | 0.51 | 19% |
| VLDL Total |  |  |  |  |  |  |  |  |  |  |  |
| rs11591147 | 1 | PCSK9 | 55505647 | 0.01 | 48% | 0.01 | 42% | 0.01 | 33% | 0.001 | 8% |
| rs10889353 | 1 | ANGPTL3 | 63118196 | 0.31 | 66% | 0.37 | 81% | 0.34 | 66% | 0.20 | 34% |
| rs673548 | 2 | APOB | 21237544 | 0.21 | >99% | 0.22 | >99% | 0.26 | 99% | 0.73 | 92% |
| rs780094 | 2 | GCKR | 27741237 | 0.43 | 61% | 0.18 | 64% | 0.33 | 66% | 0.47 | 46% |
| rs328 | 8 | LPL | 19819724 | 0.11 | 93% | 0.06 | 75% | 0.08 | 71% | 0.12 | 59% |
| rs3135506 | 11 | APOA1-A5 | 116662407 | 0.06 | >99% | 0.06 | 99% | 0.12 | >99% | 1.6*10^-4^ | 6% |
| rs1800775 | 16 | CETP | 56995236 | 0.49 | 42% | 0.57 | 67% | 0.51 | 54% | 0.53 | 34% |
| rs439401 | 19 | APOC1-APOE | 45414451 | 0.38 | 51% | 0.21 | 55% | 0.46 | 56% | 0.56 | 36% |
| rs1354163 | 5 | TIMD4\|HAVCR1 | 156420703 | 0.15 | 27% | 0.05 | 16% | 0.07 | 16% | 0.01 | 7% |
| rs255052 | 16 | LCAT | 68024995 | 0.14 | 29% | 0.21 | 36% | 0.16 | 25% | 0.09 | 13% |
| rs2865892 | 20 | MAFB | 39403896 | 0.12 | 22% | 0.14 | 23% | 0.19 | 21% | 0.28 | 17% |
| rs7923609 | 10 | JMJD1C | 65133822 | 0.49 | 31% | 0.34 | 48% | 0.31 | 36% | 0.30 | 23% |
| IDL total |  |  |  |  |  |  |  |  |  |  |  |
| rs780094 | 2 | GCKR | 27741237 | 0.43 | 5% | 0.18 | 5% | 0.33 | 5% | 0.47 | 5% |
| rs1532085 | 15 | LIPC | 58683366 | 0.37 | 5% | 0.49 | 5% | 0.39 | 5% | 0.45 | 5% |
| rs1800775 | 16 | CETP | 56995236 | 0.49 | 5% | 0.57 | 5% | 0.51 | 5% | 0.53 | 5% |
| rs1363232 | 5 | *TIMD4\|HAVCR1* | 156383422 | 0.34 | 5% | 0.45 | 5% | 0.25 | 5% | 0.16 | 5% |
| rs255049 | 16 | *LCAT* | 68013471 | 0.19 | 5% | 0.63 | 5% | 0.27 | 5% | 0.10 | 5% |
| LDL Large |  |  |  |  |  |  |  |  |  |  |  |
| rs11591147 | 1 | PCSK9 | 55505647 | 0.01 | 57% | 0.01 | 45% | 0.01 | 38% | 0.001 | 10% |
| rs1713222 | 2 | APOB | 21271323 | 0.18 | 84% | 0.27 | 81% | 0.15 | 55% | 0.003 | 8% |
| rs5744680 | 5 | HMGCR | 74879890 | 0.40 | 73% | 0.86 | 38% | 0.49 | 52% | 0.52 | 40% |
| rs2083637 | 8 | LPL | 19865175 | 0.29 | 76% | 0.20 | 53% | 0.22 | 47% | 0.21 | 35% |
| rs1535 | 11 | FADS1-3 | 61597972 | 0.33 | 66% | 0.15 | 36% | 0.52 | 48% | 0.60 | 36% |
| rs1800588 | 15 | LIPC | 58723675 | 0.22 | >99% | 0.50 | >99% | 0.47 | >99% | 0.36 | 96% |
| rs1864163 | 16 | CETP | 56997233 | 0.24 | 97% | 0.30 | 93% | 0.28 | 85% | 0.15 | 55% |
| rs8090363 | 18 | LIPG | 47133828 | 0.39 | 69% | 0.28 | 49% | 0.33 | 44% | 0.27 | 32% |
| rs6511720 | 19 | LDLR | 11202306 | 0.11 | 80% | 0.11 | 66% | 0.08 | 47% | 0.01 | 12% |
| rs4803750 | 19 | APOC1-APOE | 45247627 | 0.07 | 96% | 0.08 | 91% | 0.14 | 96% | 0.07 | 66% |
| rs4810479 | 20 | PLTP | 44545048 | 0.27 | 70% | 0.40 | 64% | 0.46 | 56% | 0.61 | 42% |
| rs7706174 | 5 | TIMD4\|HAVCR1 | 156475398 | 0.21 | 46% | 0.35 | 45% | 0.18 | 28% | 0.19 | 23% |
| rs6102085 | 20 | MAFB | 39281629 | 0.36 | 33% | 0.28 | 24% | 0.40 | 22% | 0.51 | 18% |
| LDL Small |  |  |  |  |  |  |  |  |  |  |  |
| rs646776 | 1 | CELSR2/PSRC1/SPRT1 | 109818530 | 0.21 | 95% | 0.36 | 92% | 0.24 | 78% | 0.06 | 28% |
| rs780094 | 2 | GCKR | 27741237 | 0.43 | >99% | 0.18 | 92% | 0.33 | 95% | 0.47 | 86% |
| rs2083637 | 8 | LPL | 19865175 | 0.29 | >99% | 0.20 | 89% | 0.22 | 84% | 0.21 | 64% |
| rs6982636 | 8 | TRIB1 | 126479315 | 0.46 | 97% | 0.37 | 86% | 0.40 | 79% | 0.54 | 62% |
| rs3135506 | 11 | APOA1-A5 | 116662407 | 0.06 | >99% | 0.06 | 98% | 0.12 | >99% | 1.6*10^-4^ | 7% |
| rs1800588 | 15 | LIPC | 58723675 | 0.22 | 96% | 0.50 | 95% | 0.47 | 89% | 0.36 | 70% |
| rs1800775 | 16 | CETP | 56995236 | 0.49 | >99% | 0.57 | >99% | 0.51 | >99% | 0.53 | 98% |
| rs769449 | 19 | APOC1-APOE | 45410002 | 0.12 | >99% | 0.04 | 85% | 0.07 | 92% | 0.09 | 85% |
| rs6065906 | 20 | PLTP | 44554015 | 0.19 | 99% | 0.17 | 92% | 0.13 | 77% | 0.03 | 24% |
| rs7706174 | 5 | TIMD4\|HAVCR1 | 156475398 | 0.21 | 34% | 0.35 | 32% | 0.18 | 21% | 0.19 | 16% |
| rs7200210 | 16 | LCAT | 67981896 | 0.05 | 33% | 0.36 | 68% | 0.10 | 32% | 0.01 | 9% |
| rs2865892 | 20 | MAFB | 39403896 | 0.12 | 63% | 0.14 | 52% | 0.19 | 52% | 0.28 | 45% |
| LDL total |  |  |  |  |  |  |  |  |  |  |  |
| rs11591147 | 1 | PCSK9 | 55505647 | 0.01 | 5% | 0.01 | 5% | 0.01 | 5% | 0.001 | 5% |
| rs646776 | 1 | CELSR2/PSRC1/SPRT1 | 109818530 | 0.21 | 5% | 0.36 | 5% | 0.24 | 5% | 0.06 | 5% |
| rs312985 | 2 | APOB | 21378805 | 0.22 | 5% | 0.36 | 5% | 0.19 | 5% | 0.01 | 5% |
| rs780094 | 2 | GCKR | 27741237 | 0.43 | 5% | 0.18 | 5% | 0.33 | 5% | 0.47 | 5% |
| rs6982636 | 8 | TRIB1 | 126479315 | 0.46 | 5% | 0.37 | 5% | 0.40 | 5% | 0.54 | 5% |
| rs3135506 | 11 | APOA1-A5 | 116662407 | 0.06 | 5% | 0.06 | 5% | 0.12 | 5% | 0.00 | 5% |
| rs1800775 | 16 | CETP | 56995236 | 0.49 | 5% | 0.57 | 5% | 0.51 | 5% | 0.53 | 5% |
| rs6511720 | 19 | LDLR | 11202306 | 0.11 | 5% | 0.11 | 5% | 0.08 | 5% | 0.01 | 5% |
| rs769449 | 19 | APOC1-APOE | 45410002 | 0.12 | 5% | 0.04 | 5% | 0.07 | 5% | 0.09 | 5% |
| rs1363232 | 5 | TIMD4\|HAVCR1 | 156383422 | 0.34 | 5% | 0.45 | 5% | 0.25 | 5% | 0.16 | 5% |
| rs2865892 | 20 | MAFB | 39403896 | 0.12 | 5% | 0.14 | 5% | 0.19 | 5% | 0.28 | 5% |
| HDL Large |  |  |  |  |  |  |  |  |  |  |  |
| rs331 | 8 | LPL | 19820405 | 0.29 | 96% | 0.36 | 88% | 0.25 | 89% | 0.21 | 61% |
| rs174546 | 11 | FADS1-3 | 61569830 | 0.33 | 87% | 0.10 | 41% | 0.51 | 85% | 0.59 | 59% |
| rs7307277 | 12 | CCDC92/DNAH10/ZNF664 | 124475156 | 0.36 | 76% | 0.35 | 59% | 0.31 | 66% | 0.08 | 22% |
| rs1800588 | 15 | LIPC | 58723675 | 0.22 | >99% | 0.50 | >99% | 0.47 | >99% | 0.36 | >99% |
| rs1800775 | 16 | CETP | 56995236 | 0.49 | >99% | 0.57 | >99% | 0.51 | >99% | 0.53 | >99% |
| rs4939883 | 18 | LIPG | 47167214 | 0.17 | 83% | 0.46 | 86% | 0.18 | 77% | 0.20 | 56% |
| rs6065904 | 20 | PLTP | 44534651 | 0.23 | >99% | 0.23 | >99% | 0.35 | >99% | 0.30 | 99% |
| rs6873053 | 5 | TIMD4\|HAVCR1 | 156376703 | 0.07 | 20% | 0.02 | 10% | 0.03 | 12% | 0.002 | 6% |
| rs2271293 | 16 | LCAT | 67902070 | 0.11 | 25% | 0.08 | 17% | 0.12 | 24% | 0.02 | 9% |
| rs2865879 | 20 | MAFB | 39385254 | 0.35 | 27% | 0.17 | 16% | 0.25 | 21% | 0.29 | 16% |
| HDL Medium |  |  |  |  |  |  |  |  |  |  |  |
| rs4073054 | 1 | APOA2 | 161200487 | 0.37 | 11% | 0.22 | 10% | 0.25 | 9% | 0.11 | 7% |
| rs174537 | 11 | FADS1-3 | 61552680 | 0.33 | 11% | 0.10 | 8% | 0.51 | 10% | 0.59 | 9% |
| rs405509 | 19 | APOC1-APOE | 45408836 | 0.48 | 12% | 0.29 | 11% | 0.46 | 11% | 0.68 | 9% |
| rs4704810 | 5 | *TIMD4\|HAVCR1* | 156273481 | 0.37 | 7% | 0.11 | 6% | 0.41 | 7% | 0.35 | 7% |
| HDL Small |  |  |  |  |  |  |  |  |  |  |  |
| rs780094 | 2 | GCKR | 27741237 | 0.43 | 97% | 0.18 | 92% | 0.33 | 87% | 0.47 | 68% |
| rs518181 | 11 | APOA1-A5 | 116772787 | 0.39 | 99% | 0.82 | 85% | 0.60 | 94% | 0.79 | 61% |
| rs1800588 | 15 | LIPC | 58723675 | 0.22 | 90% | 0.50 | 93% | 0.47 | 91% | 0.36 | 65% |
| rs4810479 | 20 | PLTP | 44545048 | 0.27 | >99% | 0.40 | >99% | 0.46 | >99% | 0.61 | 99% |
| rs1109166 | 16 | *LCAT* | 67977382 | 0.17 | 41% | 0.61 | 50% | 0.26 | 40% | 0.10 | 17% |
| HDL total |  |  |  |  |  |  |  |  |  |  |  |
| rs1260326 | 2 | GCKR | 27730940 | 0.44 | 99% | 0.16 | 84% | 0.33 | 95% | 0.50 | 87% |
| rs518181 | 11 | APOA1-A5 | 116772787 | 0.39 | 92% | 0.82 | 67% | 0.60 | 83% | 0.79 | 52% |
| rs7499892 | 16 | CETP | 57006590 | 0.18 | 89% | 0.38 | 93% | 0.25 | 87% | 0.17 | 59% |
| rs6065906 | 20 | PLTP | 44554015 | 0.19 | 99% | 0.17 | 95% | 0.13 | 99% | 0.03 | 31% |
| rs1109166 | 16 | *LCAT* | 67977382 | 0.17 | 55% | 0.61 | 58% | 0.26 | 55% | 0.10 | 24% |
| rs6016408 | 20 | *MAFB* | 39291059 | 0.002 | 6% | 0.24 | 18% | 0.21 | 17% | 0.07 | 10% |
| rs7938647 | 11 | SBF2 | 10061423 | 0.28 | 49% | 0.06 | 18% | 0.17 | 31% | 0.002 | 6% |
| VLDL diameter |  |  |  |  |  |  |  |  |  |  |  |
| rs676210 | 2 | APOB | 21231524 | 0.21 | 73% | 0.15 | 64% | 0.24 | 55% | 0.73 | 49% |
| rs1260326 | 2 | GCKR | 27730940 | 0.44 | 91% | 0.16 | 71% | 0.33 | 68% | 0.50 | 62% |
| rs983309 | 8 | intergenic, PPP1R3B | 9177732 | 0.10 | 71% | 0.29 | 95% | 0.22 | 74% | 0.01 | 12% |
| LDL diameter |  |  |  |  |  |  |  |  |  |  |  |
| rs780094 | 2 | GCKR | 27741237 | 0.43 | 99% | 0.18 | 91% | 0.33 | 92% | 0.47 | 86% |
| rs2083637 | 8 | LPL | 19865175 | 0.29 | 99% | 0.20 | 93% | 0.22 | 85% | 0.21 | 71% |
| rs3135506 | 11 | APOA1-A5 | 116662407 | 0.06 | 99% | 0.06 | 96% | 0.12 | 99% | 1.6*10^-4^ | 6% |
| rs1800588 | 15 | LIPC | 58723675 | 0.22 | >99% | 0.50 | >99% | 0.47 | >99% | 0.36 | 99% |
| rs1800775 | 16 | CETP | 56995236 | 0.49 | >99% | 0.57 | >99% | 0.51 | >99% | 0.53 | >99% |
| rs4939883 | 18 | LIPG | 47167214 | 0.17 | 95% | 0.46 | 99% | 0.18 | 80% | 0.20 | 70% |
| rs6065906 | 20 | PLTP | 44554015 | 0.19 | >99% | 0.17 | 98% | 0.13 | 88% | 0.03 | 32% |
| rs7706174 | 5 | TIMD4\|HAVCR1 | 156475398 | 0.21 | 58% | 0.35 | 61% | 0.18 | 36% | 0.19 | 29% |
| rs7200210 | 16 | LCAT | 67981896 | 0.05 | 45% | 0.36 | 86% | 0.10 | 41% | 0.01 | 11% |
| rs2865892 | 20 | MAFB | 39403896 | 0.12 | 36% | 0.14 | 33% | 0.19 | 30% | 0.28 | 28% |
| HDL diameter |  |  |  |  |  |  |  |  |  |  |  |
| rs780094 | 2 | GCKR | 27741237 | 0.43 | 74% | 0.18 | 38% | 0.33 | 55% | 0.47 | 42% |
| rs331 | 8 | LPL | 19820405 | 0.29 | 88% | 0.36 | 72% | 0.25 | 70% | 0.21 | 47% |
| rs1535 | 11 | FADS1-3 | 61597972 | 0.33 | 70% | 0.15 | 34% | 0.52 | 59% | 0.60 | 41% |
| rs1800588 | 15 | LIPC | 58723675 | 0.22 | >99% | 0.50 | >99% | 0.47 | >99% | 0.36 | 99% |
| rs1800775 | 16 | CETP | 56995236 | 0.49 | >99% | 0.57 | >99% | 0.51 | >99% | 0.53 | 99% |
| rs4939883 | 18 | LIPG | 47167214 | 0.17 | 75% | 0.46 | 75% | 0.18 | 61% | 0.20 | 46% |
| rs6065906 | 20 | PLTP | 44554015 | 0.19 | >99% | 0.17 | >99% | 0.13 | 98% | 0.03 | 43% |
| rs4986970 | 16 | LCAT | 67976320 | 0.03 | 21% | 0.01 | 10% | 0.01 | 11% | 0.002 | 7% |
| rs2865892 | 20 | MAFB | 39403896 | 0.12 | 24% | 0.14 | 19% | 0.19 | 24% | 0.28 | 20% |

Abbreviations: ^a^: EA: European American; ^b^: AA: African American; ^c^: HA – Hispanic American; ^d^: CHN: Chinese American; ^e^: CAF: coded allele frequency

Summary: 54 out of 131(41%) associations reached 80% power in EAs; 47 out of 131(36%) associations reached 80% power in AAs; 38 out of 131(29%) associations reached 80% power in Has; 17 out of 131(13%) associations reached 80% power in CHNs.

**S 3** **Table** Power to reject the null hypothesis that “genetic variants underlying lipoprotein measures in European Americans are the same as those in non-Caucasian populations”

| Independent variable | R^2^ of GRS | Power to replicate | Power to reject null hypothesis | | |
| --- | --- | --- | --- | --- | --- |
|  |  | European Americans (N=2506) | African Americans (N=1610) | Hispanic Americans (N=1448) | Chinese Americans (N=775) |
| VLDL large | .041 | >99% | >99% | >99% | >99% |
| VLDL medium | .060 | >99% | >99% | >99% | >99% |
| VLDL small | .074 | >99% | >99% | >99% | >99% |
| VLDL total | .086 | >99% | >99% | >99% | >99% |
| IDL total | .035 | >99% | >99% | >99% | >99% |
| LDL large | .114 | >99% | >99% | >99% | >99% |
| LDL small | .094 | >99% | >99% | >99% | >99% |
| LDL total | .150 | >99% | >99% | >99% | >99% |
| HDL large | .125 | >99% | >99% | >99% | >99% |
| HDL medium | .044 | >99% | >99% | >99% | >99% |
| HDL small | .057 | >99% | >99% | >99% | >99% |
| HDL total | .056 | >99% | >99% | >99% | >99% |
| VLDL mean size | .025 | >99% | >99% | >99% | 96% |
| LDL mean size | .087 | >99% | >99% | >99% | >99% |
| HDL mean size | .117 | >99% | >99% | >99% | >99% |

**S 4 Table** SNP-phenotype associations using proxy SNPS in the African American population of MESA

| Gene | Chr | SNPs | Proxy SNPs | distance | African-American | | |  |
| --- | --- | --- | --- | --- | --- | --- | --- | --- |
|  |  |  |  |  | beta | P | Q |  |
| VLDL Large | |  |  |  |  |  |  |  |
| GCKR | 2p23.3 | rs1260326 |  |  | 0.12 | 0.04^*^ | 0.30 |  |
| LPL | 8p21.3 | rs1059611 |  |  | -0.08 | 0.13 | 0.44 |  |
|  |  |  | rs3735964 | 518 | -0.10 | 0.26 | 0.56 |  |
|  |  |  | rs12679834 | 4130 | -0.16 | 0.04 | 0.28 |  |
|  |  |  | rs17482753 | 8083 | -0.10 | 0.28 | 0.58 |  |
|  |  |  | rs12678919 | 19659 | -0.11 | 0.10 | 0.44 |  |
|  |  |  | rs12682115 | 22497 | -0.10 | 0.07 | 0.38 |  |
|  |  |  | rs10503669 | 23127 | -0.11 | 0.25 | 0.55 |  |
|  |  |  | rs17411024 | 27571 | -0.08 | 0.15 | 0.44 |  |
|  |  |  | rs7016880 | 52183 | -0.25 | 0.009 | 0.14 |  |
|  |  |  | rs7816447 | 58387 | -0.20 | 0.04 | 0.28 |  |
| APOA1-A5 | 11q23.3 | rs3135506 |  |  | 0.26 | 0.008^*^ | 0.14 |  |
| APOC1-APOE | 19q13.32 | rs439401 |  |  | -0.01 | 0.83 | 0.95 |  |
| TIMD4\|HAVCR1 | 5q33.3 | rs1354163 |  |  | 0.10 | 0.26 | 0.56 |  |
|  |  |  | rs1501910 | 1913 | 0.15 | 0.07 | 0.39 |  |
|  |  |  | rs6896499 | 10622 | 0.07 | 0.31 | 0.61 |  |
|  |  |  | rs1948759 | 21954 | 0.09 | 0.08 | 0.41 |  |
|  |  |  | rs2902132 | 24822 | 0.07 | 0.34 | 0.64 |  |
|  |  |  | rs905211 | 26515 | 0.10 | 0.10 | 0.44 |  |
|  |  |  | rs1393209 | 56837 | 0.06 | 0.39 | 0.70 |  |
|  |  |  | rs1553316 | 58806 | 0.05 | 0.41 | 0.71 |  |
|  |  |  | rs7731951 | 28982 | 0.06 | 0.40 | 0.71 |  |
|  |  |  | rs1553317 | 58721 | 0.05 | 0.47 | 0.76 |  |
| LCAT | 16q22.1 | rs1109166 |  |  | 0.03 | 0.50 | 0.78 |  |
|  |  |  | rs7199443 | 136253 | 0.05 | 0.28 | 0.59 |  |
| MAFB | 20q12 | rs1076759 |  |  | 0.002 | 0.97 | 0.99 |  |
|  |  |  | rs6102127 | 22078 | -0.04 | 0.58 | 0.84 |  |
| VLDL Medium | |  |  |  |  |  |  |  |
| ANGPTL3 | 1p31.3 | rs10889353 |  |  | -0.11 | 0.09 | 0.44 |  |
|  |  |  | rs1168089 | 4477 | 0.10 | 0.14 | 0.44 |  |
|  |  |  | rs7539035 | 11429 | 0.09 | 0.18 | 0.45 |  |
|  |  |  | rs6587980 | 27338 | 0.05 | 0.45 | 0.74 |  |
|  |  |  | rs1748195 | 68603 | 0.09 | 0.15 | 0.44 |  |
|  |  |  | rs4329540 | 91172 | 0.10 | 0.14 | 0.44 |  |
|  |  |  | rs1168018 | 117076 | 0.09 | 0.16 | 0.44 |  |
|  |  |  | rs1781195 | 129224 | 0.09 | 0.14 | 0.44 |  |
|  |  |  | rs656297 | 210601 | 0.08 | 0.24 | 0.54 |  |
|  |  |  | rs10889333 | 161166 | -0.07 | 0.27 | 0.58 |  |
|  |  |  | rs1168099 | 4663 | 0.09 | 0.15 | 0.44 |  |
|  |  |  | rs10889347 | 42370 | 0.09 | 0.16 | 0.44 |  |
|  |  |  | rs6678483 | 43754 | 0.09 | 0.15 | 0.44 |  |
|  |  |  | rs6675401 | 44221 | 0.09 | 0.15 | 0.44 |  |
|  |  |  | rs10789117 | 45931 | 0.09 | 0.15 | 0.44 |  |
|  |  |  | rs1168013 | 121358 | 0.09 | 0.17 | 0.45 |  |
|  |  |  | rs10158897 | 205277 | -0.05 | 0.47 | 0.76 |  |
|  |  |  | rs6690733 | 57885 | 0.10 | 0.14 | 0.44 |  |
|  |  |  | rs1748201 | 72690 | 0.09 | 0.16 | 0.44 |  |
|  |  |  | rs10889337 | 137589 | 0.05 | 0.42 | 0.71 |  |
|  |  |  | rs1168032 | 150449 | 0.10 | 0.13 | 0.44 |  |
|  |  |  | rs10889335 | 158095 | -0.10 | 0.13 | 0.44 |  |
|  |  |  | rs1979722 | 178099 | -0.04 | 0.50 | 0.78 |  |
|  |  |  | rs1748199 | 61060 | 0.10 | 0.12 | 0.44 |  |
|  |  |  | rs3913007 | 196610 | -0.05 | 0.48 | 0.76 |  |
|  |  |  | rs631106 | 216389 | 0.08 | 0.24 | 0.54 |  |
|  |  |  | rs637723 | 211468 | 0.08 | 0.25 | 0.55 |  |
|  |  |  | rs4350231 | 195536 | -0.05 | 0.46 | 0.75 |  |
|  |  |  | rs624660 | 197808 | 0.08 | 0.25 | 0.55 |  |
|  |  |  | rs583609 | 201400 | 0.08 | 0.25 | 0.55 |  |
|  |  |  | rs11207969 | 206445 | 0.04 | 0.51 | 0.79 |  |
|  |  |  | rs1167998 | 186564 | 0.08 | 0.23 | 0.54 |  |
|  |  |  | rs10889332 | 167338 | 0.03 | 0.65 | 0.86 |  |
|  |  |  | rs880694 | 55088 | 0.08 | 0.21 | 0.50 |  |
|  |  |  | rs11207997 | 56290 | 0.05 | 0.47 | 0.76 |  |
|  |  |  | rs1168040 | 157621 | 0.10 | 0.14 | 0.44 |  |
|  |  |  | rs11208000 | 9822 | 0.09 | 0.15 | 0.44 |  |
|  |  |  | rs12136083 | 44877 | -0.11 | 0.10 | 0.44 |  |
|  |  |  | rs7518497 | 59500 | -0.12 | 0.08 | 0.40 |  |
|  |  |  | rs1168114 | 37847 | 0.08 | 0.19 | 0.47 |  |
|  |  |  | rs1168113 | 37902 | 0.10 | 0.12 | 0.44 |  |
|  |  |  | rs6682423 | 52867 | 0.08 | 0.21 | 0.51 |  |
|  |  |  | rs4409689 | 59169 | 0.09 | 0.18 | 0.45 |  |
|  |  |  | rs9787151 | 60942 | 0.08 | 0.19 | 0.46 |  |
| APOB | 2p24.1 | rs673548 |  |  | -0.07 | 0.38 | 0.69 |  |
|  |  |  | rs6754295 | 31361 | -0.004 | 0.95 | 0.99 |  |
| GCKR | 2p23.3 | rs1260326 |  |  | 0.20 | 0.02^*^ | 0.22 |  |
| MLXIPL | 7q11.23 | rs2240466 |  |  | -0.44 | 0.007^**^ | 0.14 |  |
| LPL | 8p21.3 | rs1059611 |  |  | -0.15 | 0.07 | 0.38 |  |
|  |  |  | rs3735964 | 518 | -0.09 | 0.53 | 0.79 |  |
|  |  |  | rs12679834 | 4130 | -0.16 | 0.15 | 0.44 |  |
|  |  |  | rs17482753 | 8083 | -0.08 | 0.56 | 0.83 |  |
|  |  |  | rs12678919 | 19659 | -0.03 | 0.77 | 0.92 |  |
|  |  |  | rs12682115 | 22497 | -0.06 | 0.46 | 0.75 |  |
|  |  |  | rs10503669 | 23127 | -0.01 | 0.96 | 0.99 |  |
|  |  |  | rs17411024 | 27571 | -0.04 | 0.67 | 0.88 |  |
|  |  |  | rs7016880 | 52183 | -0.20 | 0.16 | 0.44 |  |
|  |  |  | rs7816447 | 58387 | -0.14 | 0.31 | 0.61 |  |
| APOA1-A5 | 11q23.3 | rs3135506 |  |  | 0.37 | 0.01^*^ | 0.15 |  |
| JMJD1C | 10q21.3 | rs12768534 |  |  | 0.04 | 0.52 | 0.79 |  |
|  |  |  | rs10822184 | 3744 | -0.03 | 0.68 | 0.89 |  |
|  |  |  | rs10822186 | 9486 | -0.02 | 0.79 | 0.93 |  |
|  |  |  | rs10761785 | 22131 | NA | NA | NA |  |
| TIMD4\|HAVCR1 | 5q33.3 | rs1354163 |  |  | 0.20 | 0.15 | 0.44 |  |
|  |  |  | rs1501910 | 1913 | 0.19 | 0.12 | 0.44 |  |
|  |  |  | rs6896499 | 10622 | 0.05 | 0.61 | 0.85 |  |
|  |  |  | rs1948759 | 21954 | 0.11 | 0.13 | 0.44 |  |
|  |  |  | rs2902132 | 24822 | 0.04 | 0.70 | 0.90 |  |
|  |  |  | rs905211 | 26515 | 0.10 | 0.24 | 0.54 |  |
|  |  |  | rs1393209 | 56837 | 0.07 | 0.52 | 0.79 |  |
|  |  |  | rs1553316 | 58806 | 0.05 | 0.59 | 0.84 |  |
|  |  |  | rs7731951 | 28982 | 0.04 | 0.73 | 0.92 |  |
|  |  |  | rs1553317 | 58721 | 0.04 | 0.70 | 0.90 |  |
| LCAT | 16q22.1 | rs255052 |  |  | -0.06 | 0.45 | 0.74 |  |
|  |  |  | rs255048 | 12146 | 0.07 | 0.74 | 0.92 |  |
| MAFB | 20q12 | rs2865892 |  |  | -0.04 | 0.65 | 0.86 |  |
|  |  |  | rs2206665 | 5724 | -0.05 | 0.62 | 0.85 |  |
| VLDL Small | |  |  |  |  |  |  |  |
| PCSK9 | 1p32.3 | rs11591147 |  |  | -0.05 | 0.94 | 0.98 |  |
| ANGPTL3 | 1p31.3 | rs1167998 |  |  | 0.04 | 0.57 | 0.84 |  |
|  |  |  | rs4350231 | 8972 | -0.09 | 0.16 | 0.44 |  |
|  |  |  | rs11207969 | 19881 | 0.03 | 0.59 | 0.84 |  |
|  |  |  | rs3913007 | 10046 | -0.09 | 0.15 | 0.44 |  |
|  |  |  | rs10889332 | 19226 | 0.02 | 0.76 | 0.92 |  |
|  |  |  | rs10889337 | 48975 | 0.00 | 0.99 | 1.00 |  |
|  |  |  | rs10889333 | 25398 | -0.11 | 0.10 | 0.44 |  |
|  |  |  | rs10889335 | 28469 | -0.12 | 0.05 | 0.35 |  |
|  |  |  | rs12116574 | 106205 | -0.12 | 0.04 | 0.31 |  |
|  |  |  | rs3850634 | 118966 | -0.12 | 0.05 | 0.31 |  |
|  |  |  | rs4587594 | 202298 | -0.10 | 0.11 | 0.44 |  |
|  |  |  | rs11208004 | 213807 | -0.11 | 0.10 | 0.44 |  |
|  |  |  | rs10158897 | 18713 | -0.09 | 0.15 | 0.44 |  |
|  |  |  | rs1168018 | 69488 | 0.03 | 0.57 | 0.84 |  |
|  |  |  | rs6587980 | 159226 | 0.01 | 0.89 | 0.97 |  |
|  |  |  | rs1979722 | 8465 | -0.09 | 0.17 | 0.45 |  |
|  |  |  | rs10789119 | 166603 | -0.13 | 0.04 | 0.29 |  |
|  |  |  | rs1570694 | 171585 | -0.10 | 0.12 | 0.44 |  |
|  |  |  | rs995000 | 175894 | -0.14 | 0.03 | 0.25 |  |
|  |  |  | rs10889353 | 186564 | -0.13 | 0.04 | 0.28 |  |
|  |  |  | rs10159255 | 89184 | -0.09 | 0.12 | 0.44 |  |
|  |  |  | rs11207995 | 117919 | -0.12 | 0.06 | 0.36 |  |
|  |  |  | rs11207997 | 130274 | 0.01 | 0.88 | 0.97 |  |
|  |  |  | rs1168114 | 224411 | 0.05 | 0.42 | 0.71 |  |
|  |  |  | rs1168113 | 224466 | 0.04 | 0.52 | 0.79 |  |
|  |  |  | rs6682423 | 239431 | 0.05 | 0.42 | 0.71 |  |
|  |  |  | rs4409689 | 245733 | 0.05 | 0.41 | 0.71 |  |
|  |  |  | rs12136083 | 231441 | -0.14 | 0.04 | 0.28 |  |
|  |  |  | rs7518497 | 246064 | -0.12 | 0.06 | 0.35 |  |
|  |  |  | rs1168041 | 28618 | 0.03 | 0.66 | 0.87 |  |
|  |  |  | rs880694 | 241652 | 0.05 | 0.42 | 0.71 |  |
|  |  |  | rs9787151 | 247506 | 0.05 | 0.41 | 0.71 |  |
| CELSR2/PSRC1/SPRT1 | 1p13.3 | rs646776 |  |  | -0.006 | 0.92 | 0.97 |  |
|  |  |  | rs7528419 | 1338 | -0.002 | 0.97 | 0.99 |  |
|  |  |  | rs599839 | 3636 | 0.11 | 0.12 | 0.44 |  |
| APOB | 2p24.1 | rs6754295 |  |  | -0.11 | 0.09 | 0.44 |  |
|  |  |  | rs6544366 | 2166 | -0.18 | 0.03 | 0.28 |  |
|  |  |  | rs11902417 | 7291 | -0.10 | 0.17 | 0.45 |  |
|  |  |  | rs6728178 | 12254 | -0.10 | 0.18 | 0.45 |  |
|  |  |  | rs676210 | 25341 | -0.24 | 0.00 | 0.13 |  |
|  |  |  | rs673548 | 31361 | -0.12 | 0.10 | 0.44 |  |
| LPL | 8p21.3 | rs328 |  |  | -0.04 | 0.76 | 0.92 |  |
| ABO | 9q34.2 | rs507666 |  |  | 0.13 | 0.19 | 0.46 |  |
| APOA1-A5 | 11q23.3 | rs3135506 |  |  | 0.17 | 0.21 | 0.51 |  |
| CETP | 16q13 | rs1800775 |  |  | -0.09 | 0.19 | 0.47 |  |
| LDLR | 19p13.2 | rs6511720 |  |  | -0.04 | 0.76 | 0.92 |  |
|  |  |  | rs2228671 | 8606 | 0.16 | 0.44 | 0.73 |  |
| APOC1-APOE | 19q13.32 | rs439401 |  |  | -0.03 | 0.75 | 0.92 |  |
| TIMD4\|HAVCR1 | 5q33.3 | rs1363232 |  |  | -0.07 | 0.28 | 0.58 |  |
|  |  |  | rs7724832 | 2052 | -0.04 | 0.55 | 0.82 |  |
|  |  |  | rs6874202 | 8206 | -0.04 | 0.50 | 0.78 |  |
|  |  |  | rs12657266 | 12581 | -0.04 | 0.51 | 0.78 |  |
|  |  |  | rs1501908 | 14747 | -0.04 | 0.50 | 0.78 |  |
|  |  |  | rs7717984 | 14251 | 0.02 | 0.76 | 0.92 |  |
| LCAT | 16q22.1 | rs255052 |  |  | 7.04*10-4 | 0.99 | 1.00 |  |
|  |  |  | rs255048 | 12146 | -0.03 | 0.87 | 0.97 |  |
| MAFB | 20q12 | rs6102085 |  |  | 0.05 | 0.44 | 0.73 |  |
|  |  |  | rs6016404 | 1091 | -0.02 | 0.80 | 0.93 |  |
| VLDL Total | |  |  |  |  |  |  |  |
| PCSK9 | 1p32.3 | rs11591147 |  |  | -0.12 | 0.88 | 0.97 |  |
| ANGPTL3 | 1p31.3 | rs10889353 |  |  | -0.19 | 0.01^*^ | 0.16 |  |
|  |  |  | rs1168089 | 4477 | 0.13 | 0.08 | 0.40 |  |
|  |  |  | rs7539035 | 11429 | 0.11 | 0.16 | 0.44 |  |
|  |  |  | rs6587980 | 27338 | 0.06 | 0.40 | 0.71 |  |
|  |  |  | rs1748195 | 68603 | 0.11 | 0.15 | 0.44 |  |
|  |  |  | rs4329540 | 91172 | 0.11 | 0.14 | 0.44 |  |
|  |  |  | rs1168018 | 117076 | 0.11 | 0.15 | 0.44 |  |
|  |  |  | rs1781195 | 129224 | 0.11 | 0.16 | 0.44 |  |
|  |  |  | rs656297 | 210601 | 0.10 | 0.18 | 0.45 |  |
|  |  |  | rs10889333 | 161166 | -0.14 | 0.06 | 0.38 |  |
|  |  |  | rs1168099 | 4663 | 0.11 | 0.13 | 0.44 |  |
|  |  |  | rs10889347 | 42370 | 0.11 | 0.14 | 0.44 |  |
|  |  |  | rs6678483 | 43754 | 0.11 | 0.14 | 0.44 |  |
|  |  |  | rs6675401 | 44221 | 0.11 | 0.14 | 0.44 |  |
|  |  |  | rs10789117 | 45931 | 0.11 | 0.14 | 0.44 |  |
|  |  |  | rs1168013 | 121358 | 0.10 | 0.17 | 0.45 |  |
|  |  |  | rs10158897 | 205277 | -0.11 | 0.14 | 0.44 |  |
|  |  |  | rs6690733 | 57885 | 0.11 | 0.13 | 0.44 |  |
|  |  |  | rs1748201 | 72690 | 0.11 | 0.15 | 0.44 |  |
|  |  |  | rs10889337 | 137589 | 0.06 | 0.41 | 0.71 |  |
|  |  |  | rs1168032 | 150449 | 0.11 | 0.14 | 0.44 |  |
|  |  |  | rs10889335 | 158095 | -0.17 | 0.02 | 0.23 |  |
|  |  |  | rs1979722 | 178099 | -0.11 | 0.16 | 0.44 |  |
|  |  |  | rs1748199 | 61060 | 0.12 | 0.11 | 0.44 |  |
|  |  |  | rs3913007 | 196610 | -0.11 | 0.14 | 0.44 |  |
|  |  |  | rs631106 | 216389 | 0.10 | 0.17 | 0.45 |  |
|  |  |  | rs637723 | 211468 | 0.10 | 0.18 | 0.45 |  |
|  |  |  | rs4350231 | 195536 | -0.11 | 0.14 | 0.44 |  |
|  |  |  | rs624660 | 197808 | 0.10 | 0.18 | 0.45 |  |
|  |  |  | rs583609 | 201400 | 0.10 | 0.18 | 0.45 |  |
|  |  |  | rs11207969 | 206445 | 0.07 | 0.34 | 0.64 |  |
|  |  |  | rs1167998 | 186564 | 0.10 | 0.17 | 0.45 |  |
|  |  |  | rs10889332 | 167338 | 0.05 | 0.49 | 0.77 |  |
|  |  |  | rs880694 | 55088 | 0.12 | 0.11 | 0.44 |  |
|  |  |  | rs11207997 | 56290 | 0.06 | 0.42 | 0.72 |  |
|  |  |  | rs1168040 | 157621 | 0.11 | 0.16 | 0.44 |  |
|  |  |  | rs11208000 | 9822 | 0.12 | 0.13 | 0.44 |  |
|  |  |  | rs12136083 | 44877 | -0.21 | 0.008^*^ | 0.14 |  |
|  |  |  | rs7518497 | 59500 | -0.21 | 0.008^*^ | 0.14 |  |
|  |  |  | rs1168114 | 37847 | 0.12 | 0.11 | 0.44 |  |
|  |  |  | rs1168113 | 37902 | 0.12 | 0.10 | 0.44 |  |
|  |  |  | rs6682423 | 52867 | 0.12 | 0.11 | 0.44 |  |
|  |  |  | rs4409689 | 59169 | 0.12 | 0.10 | 0.44 |  |
|  |  |  | rs9787151 | 60942 | 0.12 | 0.10 | 0.44 |  |
| APOB | 2p24.1 | rs673548 |  |  | -0.14 | 0.11 | 0.44 |  |
|  |  |  | rs6754295 | 31361 | -0.07 | 0.39 | 0.69 |  |
| GCKR | 2p23.3 | rs780094 |  |  | 0.23 | 0.01^*^ | 0.16 |  |
| LPL | 8p21.3 | rs328 |  |  | -0.12 | 0.42 | 0.71 |  |
| APOA1-A5 | 11q23.3 | rs3135506 |  |  | 0.42 | 0.01^*^ | 0.16 |  |
| CETP | 16q13 | rs1800775 |  |  | 0.05 | 0.55 | 0.82 |  |
| APOC1-APOE | 19q13.32 | rs439401 |  |  | 0.05 | 0.68 | 0.89 |  |
| TIMD4\|HAVCR1 | 5q33.3 | rs1354163 |  |  | 0.17 | 0.28 | 0.58 |  |
|  |  |  | rs1501910 | 1913 | 0.21 | 0.14 | 0.44 |  |
|  |  |  | rs6896499 | 10622 | 0.03 | 0.83 | 0.95 |  |
|  |  |  | rs1948759 | 21954 | 0.13 | 0.12 | 0.44 |  |
|  |  |  | rs2902132 | 24822 | 0.02 | 0.89 | 0.97 |  |
|  |  |  | rs905211 | 26515 | 0.11 | 0.28 | 0.58 |  |
|  |  |  | rs1393209 | 56837 | 0.06 | 0.64 | 0.87 |  |
|  |  |  | rs1553316 | 58806 | 0.05 | 0.65 | 0.87 |  |
|  |  |  | rs7731951 | 28982 | 0.02 | 0.87 | 0.97 |  |
|  |  |  | rs1553317 | 58721 | 0.03 | 0.75 | 0.92 |  |
| LCAT | 16q22.1 | rs255052 |  |  | -0.04 | 0.68 | 0.89 |  |
|  |  |  | rs255048 | 12146 | 0.04 | 0.86 | 0.96 |  |
| MAFB | 20q12 | rs2865892 |  |  | 0.02 | 0.82 | 0.94 |  |
|  |  |  | rs2206665 | 5724 | 0.01 | 0.93 | 0.98 |  |
| JMJD1C | 10q21.3 | rs7923609 |  |  | -0.09 | 0.23 | 0.53 |  |
|  |  |  | rs10822184 | 203331 | -0.04 | 0.61 | 0.85 |  |
|  |  |  | rs10822186 | 216561 | -0.02 | 0.81 | 0.94 |  |
|  |  |  | rs4746203 | 190175 | -0.03 | 0.64 | 0.87 |  |
|  |  |  | rs3847326 | 198037 | -0.04 | 0.59 | 0.84 |  |
|  |  |  | rs10761785 | 184944 | NA | NA | NA |  |
| IDL total | |  |  |  |  |  |  |  |
| GCKR | 2p23.3 | rs780094 |  |  | 0.20 | 0.28 | 0.58 |  |
| LIPC | 15q22.1 | rs1532085 |  |  | 0.15 | 0.30 | 0.61 |  |
|  |  |  | rs7350789 | 3698 | 0.11 | 0.44 | 0.74 |  |
| CETP | 16q13 | rs1800775 |  |  | 0.23 | 0.15 | 0.44 |  |
| *TIMD4\|HAVCR1* | 5q33.3 | rs1363232 |  |  | -0.10 | 0.49 | 0.78 |  |
|  |  |  | rs7724832 | 2052 | -0.05 | 0.76 | 0.92 |  |
|  |  |  | rs6874202 | 8206 | 0.17 | 0.27 | 0.58 |  |
|  |  |  | rs12657266 | 12581 | 0.17 | 0.27 | 0.58 |  |
|  |  |  | rs1501908 | 14747 | 0.19 | 0.22 | 0.52 |  |
|  |  |  | rs7717984 | 14251 | -0.05 | 0.80 | 0.93 |  |
| *LCAT* | 16q22.1 | rs255049 |  |  | -0.11 | 0.47 | 0.76 |  |
| LDL Large | |  |  |  |  |  |  |  |
| PCSK9 | 1p32.3 | rs11591147 |  |  | 42.52 | 0.66 | 0.87 |  |
| APOB | 2p24.1 | rs1713222 |  |  | -15.85 | 0.11 | 0.44 |  |
|  |  |  | rs12714264 | 5805 | -45.37 | 0.11 | 0.44 |  |
| HMGCR | 5q13.3 | rs5744680 |  |  | -2.76 | 0.83 | 0.95 |  |
|  |  |  | rs3846662 | 228806 | 2.32 | 0.86 | 0.96 |  |
|  |  |  | rs12654264 | 231287 | -2.57 | 0.77 | 0.92 |  |
|  |  |  | rs11749783 | 253808 | -1.04 | 0.91 | 0.97 |  |
|  |  |  | rs7703051 | 254403 | -0.98 | 0.91 | 0.97 |  |
|  |  |  | rs3846663 | 224164 | -3.82 | 0.70 | 0.90 |  |
|  |  |  | rs2335418 | 276411 | -15.78 | 0.14 | 0.44 |  |
|  |  |  | rs11957260 | 271158 | 0.28 | 0.98 | 1.00 |  |
| LPL | 8p21.3 | rs2083637 |  |  | -3.10 | 0.77 | 0.92 |  |
|  |  |  | rs765547 | 1099 | -11.07 | 0.36 | 0.67 |  |
|  |  |  | rs1919484 | 4501 | -10.89 | 0.37 | 0.68 |  |
|  |  |  | rs17411126 | 9903 | 0.09 | 0.99 | 1.00 |  |
|  |  |  | rs4922117 | 12589 | -0.56 | 0.96 | 0.99 |  |
|  |  |  | rs17489282 | 12657 | -0.01 | 1.00 | 1.00 |  |
|  |  |  | rs17411045 | 12813 | -0.01 | 1.00 | 1.00 |  |
|  |  |  | rs17411031 | 12865 | -0.01 | 1.00 | 1.00 |  |
|  |  |  | rs17489268 | 13130 | 0.34 | 0.97 | 0.99 |  |
|  |  |  | rs327 | 45639 | 13.84 | 0.10 | 0.44 |  |
|  |  |  | rs331 | 44770 | 10.61 | 0.23 | 0.53 |  |
|  |  |  | rs301 | 48241 | NA | NA | NA |  |
|  |  |  | rs297 | 48804 | 13.41 | 0.16 | 0.44 |  |
|  |  |  | rs3916027 | 40307 | 17.40 | 0.04 | 0.31 |  |
|  |  |  | rs12541912 | 5384 | 0.19 | 0.99 | 1.00 |  |
|  |  |  | rs4523270 | 8636 | -1.14 | 0.91 | 0.97 |  |
|  |  |  | rs2165558 | 8915 | -1.05 | 0.92 | 0.97 |  |
|  |  |  | rs15285 | 40508 | 27.12 | 0.002^*^ | 0.06 |  |
|  |  |  | rs11986942 | 2270 | -23.00 | 0.009^*^ | 0.15 |  |
|  |  |  | rs13702 | 40683 | 26.69 | 0.002^*^ | 0.07 |  |
|  |  |  | rs326 | 45736 | -20.41 | 0.02^*^ | 0.19 |  |
|  |  |  | rs1441766 | 2387 | 21.44 | 0.01^*^ | 0.16 |  |
| FADS1-3 | 11q12.2 | rs1535 |  |  | -27.69 | 0.03^*^ | 0.28 |  |
|  |  |  | rs174568 | 4156 | -39.42 | 0.008^*^ | 0.14 |  |
|  |  |  | rs174546 | 28142 | -40.98 | 0.006^*^ | 0.14 |  |
|  |  |  | rs174576 | 5538 | -7.04 | 0.47 | 0.75 |  |
|  |  |  | rs174566 | 5610 | -27.80 | 0.006^*^ | 0.14 |  |
|  |  |  | rs174577 | 6842 | -14.42 | 0.10 | 0.44 |  |
|  |  |  | rs174537 | 45292 | -36.38 | 0.01 | 0.16 |  |
|  |  |  | rs174583 | 11778 | -2.08 | 0.83 | 0.95 |  |
|  |  |  | rs102275 | 40169 | 17.94 | 0.07 | 0.38 |  |
|  |  |  | rs174535 | 46616 | -34.47 | 0.001^*^ | 0.06 |  |
|  |  |  | rs4246215 | 33673 | -43.06 | 0.003^*^ | 0.11 |  |
|  |  |  | rs174556 | 17337 | -41.94 | 0.006^*^ | 0.14 |  |
|  |  |  | rs174555 | 18212 | -42.01 | 0.006^*^ | 0.14 |  |
|  |  |  | rs174549 | 26590 | -42.39 | 0.006^*^ | 0.14 |  |
| LIPC | 15q22.1 | rs1800588 |  |  | 29.12 | 0.001^*^ | 0.06 |  |
| CETP | 16q13 | rs1864163 |  |  | -22.78 | 0.04^*^ | 0.28 |  |
| LIPG | 18q21.1 | rs8090363 |  |  | -1.98 | 0.83 | 0.95 |  |
| LDLR | 19p13.2 | rs6511720 |  |  | -19.52 | 0.31 | 0.61 |  |
|  |  |  | rs2228671 | 8606 | -15.22 | 0.61 | 0.85 |  |
| APOC1-APOE | 19q13.32 | rs4803750 |  |  | 10.37 | 0.48 | 0.76 |  |
|  |  |  | rs1531517 | 5454 | -9.34 | 0.43 | 0.72 |  |
| PLTP | 20q13.12.B | rs4810479 |  |  | -18.53 | 0.04^*^ | 0.31 |  |
| TIMD4\|HAVCR1 | 5q33.3 | rs7706174 |  |  | -4.29 | 0.64 | 0.87 |  |
|  |  |  | rs6878732 | 10142 | 1.22 | 0.89 | 0.97 |  |
|  |  |  | rs10059699 | 9244 | -2.43 | 0.79 | 0.93 |  |
| MAFB | 20q12 | rs6102085 |  |  | 1.46 | 0.88 | 0.97 |  |
|  |  |  | rs6016404 | 1091 | 0.61 | 0.95 | 0.99 |  |
| LDL Small | |  |  |  |  |  |  |  |
| CELSR2/PSRC1/SPRT1 | 1p13.3 | rs646776 |  |  | -15.70 | 0.23 | 0.54 |  |
|  |  |  | rs7528419 | 1338 | -8.12 | 0.59 | 0.84 |  |
|  |  |  | rs599839 | 3636 | 1.52 | 0.92 | 0.97 |  |
| GCKR | 2p23.3 | rs780094 |  |  | -3.61 | 0.82 | 0.94 |  |
| LPL | 8p21.3 | rs2083637 |  |  | 7.09 | 0.65 | 0.87 |  |
|  |  |  | rs765547 | 1099 | 5.88 | 0.74 | 0.92 |  |
|  |  |  | rs1919484 | 4501 | 2.78 | 0.88 | 0.97 |  |
|  |  |  | rs17411126 | 9903 | 14.60 | 0.32 | 0.62 |  |
|  |  |  | rs4922117 | 12589 | 14.59 | 0.32 | 0.62 |  |
|  |  |  | rs17489282 | 12657 | 14.96 | 0.31 | 0.61 |  |
|  |  |  | rs17411045 | 12813 | 14.96 | 0.31 | 0.61 |  |
|  |  |  | rs17411031 | 12865 | 14.96 | 0.31 | 0.61 |  |
|  |  |  | rs17489268 | 13130 | 15.60 | 0.29 | 0.59 |  |
|  |  |  | rs327 | 45639 | -2.55 | 0.84 | 0.95 |  |
|  |  |  | rs331 | 44770 | -1.60 | 0.90 | 0.97 |  |
|  |  |  | rs301 | 48241 | NA | NA | NA |  |
|  |  |  | rs297 | 48804 | -6.58 | 0.64 | 0.87 |  |
|  |  |  | rs3916027 | 40307 | -0.60 | 0.96 | 0.99 |  |
|  |  |  | rs12541912 | 5384 | 17.98 | 0.24 | 0.54 |  |
|  |  |  | rs4523270 | 8636 | 23.72 | 0.11 | 0.44 |  |
|  |  |  | rs2165558 | 8915 | 23.89 | 0.11 | 0.44 |  |
|  |  |  | rs15285 | 40508 | -10.31 | 0.41 | 0.71 |  |
|  |  |  | rs11986942 | 2270 | -7.85 | 0.54 | 0.82 |  |
|  |  |  | rs13702 | 40683 | -10.24 | 0.41 | 0.71 |  |
|  |  |  | rs326 | 45736 | 16.48 | 0.19 | 0.46 |  |
|  |  |  | rs1441766 | 2387 | 8.14 | 0.52 | 0.79 |  |
| TRIB1 | 8q24.13 | rs6982636 |  |  | -4.11 | 0.75 | 0.92 |  |
|  |  |  | rs2980856 | 2936 | -7.08 | 0.60 | 0.85 |  |
|  |  |  | rs17321515 | 7094 | 11.94 | 0.34 | 0.64 |  |
|  |  |  | rs2980869 | 8935 | 11.87 | 0.35 | 0.65 |  |
|  |  |  | rs10808546 | 16503 | -9.98 | 0.46 | 0.75 |  |
|  |  |  | rs6982502 | 47 | 5.51 | 0.76 | 0.92 |  |
|  |  |  | rs2001945 | 1337 | 5.14 | 0.74 | 0.92 |  |
| APOA1-A5 | 11q23.3 | rs3135506 |  |  | 50.81 | 0.08 | 0.40 |  |
| LIPC | 15q22.1 | rs1800588 |  |  | -37.89 | 0.005^*^ | 0.14 |  |
| CETP | 16q13 | rs1800775 |  |  | 24.85 | 0.07 | 0.38 |  |
| APOC1-APOE | 19q13.32 | rs769449 |  |  | 72.40 | 0.10 | 0.44 |  |
| PLTP | 20q13.12.B | rs6065906 |  |  | 25.20 | 0.14 | 0.44 |  |
|  |  |  | rs7679 | 22487 | 24.52 | 0.39 | 0.70 |  |
|  |  |  | rs6073972 | 36283 | 34.18 | 0.23 | 0.54 |  |
| TIMD4\|HAVCR1 | 5q33.3 | rs7706174 |  |  | 20.90 | 0.11 | 0.44 |  |
|  |  |  | rs6878732 | 10142 | -14.57 | 0.24 | 0.55 |  |
|  |  |  | rs10059699 | 9244 | 26.42 | 0.046 | 0.31 |  |
| LCAT | 16q22.1 | rs7200210 |  |  | 7.15 | 0.59 | 0.84 |  |
|  |  |  | rs6499141 | 184762 | -1.36 | 0.92 | 0.97 |  |
|  |  |  | rs7200950 | 292144 | -1.47 | 0.92 | 0.97 |  |
|  |  |  | rs7195415 | 292839 | -1.49 | 0.91 | 0.97 |  |
|  |  |  | rs9972635 | 299316 | -11.74 | 0.39 | 0.69 |  |
| MAFB | 20q12 | rs2865892 |  |  | 23.51 | 0.19 | 0.47 |  |
|  |  |  | rs2206665 | 5724 | 8.45 | 0.65 | 0.87 |  |
| LDL Total | |  |  |  |  |  |  |  |
| PCSK9 | 1p32.3 | rs11591147 |  |  | 2.14 | 0.29 | 0.59 |  |
| CELSR2/PSRC1/SPRT1 | 1p13.3 | rs646776 |  |  | -0.67 | 3.61*10^-4*^ | 0.03* |  |
| APOB | 2p24.1 | rs312985 |  |  | -0.75 | 4.67*10^-5*^ | 0.01* |  |
| GCKR | 2p23.3 | rs780094 |  |  | -0.03 | 0.90 | 0.97 |  |
| TRIB1 | 8q24.13 | rs6982636 |  |  | -0.04 | 0.82 | 0.94 |  |
|  |  |  | rs2980856 | 2936 | -0.03 | 0.89 | 0.97 |  |
|  |  |  | rs17321515 | 7094 | 0.22 | 0.22 | 0.52 |  |
|  |  |  | rs2980869 | 8935 | 0.22 | 0.23 | 0.54 |  |
|  |  |  | rs10808546 | 16503 | -0.09 | 0.65 | 0.87 |  |
|  |  |  | rs6982502 | 47 | -0.12 | 0.64 | 0.87 |  |
|  |  |  | rs2001945 | 1337 | -0.11 | 0.61 | 0.85 |  |
| APOA1-A5 | 11q23.3 | rs3135506 |  |  | 0.19 | 0.65 | 0.87 |  |
| CETP | 16q13 | rs1800775 |  |  | 0.19 | 0.34 | 0.64 |  |
| LDLR | 19p13.2 | rs6511720 |  |  | -0.16 | 0.69 | 0.89 |  |
|  |  |  | rs2228671 | 8606 | 0.32 | 0.61 | 0.85 |  |
| APOC1-APOE | 19q13.32 | rs769449 |  |  | 1.74 | 0.006^**^ | 0.14 |  |
| TIMD4\|HAVCR1 | 5q33.3 | rs1363232 |  |  | 0.05 | 0.80 | 0.93 |  |
|  |  |  | rs7724832 | 2052 | -0.14 | 0.43 | 0.72 |  |
|  |  |  | rs6874202 | 8206 | 0.02 | 0.90 | 0.97 |  |
|  |  |  | rs12657266 | 12581 | 0.02 | 0.89 | 0.97 |  |
|  |  |  | rs1501908 | 14747 | 0.06 | 0.77 | 0.92 |  |
|  |  |  | rs7717984 | 14251 | 0.13 | 0.59 | 0.84 |  |
| MAFB | 20q12 | rs2865892 |  |  | 0.18 | 0.50 | 0.78 |  |
|  |  |  | rs2206665 | 5724 | 0.03 | 0.92 | 0.97 |  |
| HDL Large | |  |  |  |  |  |  |  |
| LPL | 8p21.3 | rs331 |  |  | 0.03 | 0.22 | 0.52 |  |
|  |  |  | rs17489268 | 31640 | 0.03 | 0.27 | 0.58 |  |
|  |  |  | rs17411031 | 31905 | 0.03 | 0.29 | 0.59 |  |
|  |  |  | rs17411045 | 31957 | 0.03 | 0.29 | 0.59 |  |
|  |  |  | rs17489282 | 32113 | 0.03 | 0.29 | 0.59 |  |
|  |  |  | rs4922117 | 32181 | 0.03 | 0.29 | 0.59 |  |
|  |  |  | rs17411126 | 34867 | 0.03 | 0.28 | 0.59 |  |
|  |  |  | rs2083637 | 44770 | 0.02 | 0.38 | 0.69 |  |
|  |  |  | rs765547 | 45869 | 0.02 | 0.42 | 0.72 |  |
|  |  |  | rs1919484 | 49271 | 0.03 | 0.29 | 0.59 |  |
|  |  |  | rs15285 | 4262 | 0.03 | 0.17 | 0.45 |  |
|  |  |  | rs13702 | 4087 | 0.03 | 0.17 | 0.45 |  |
|  |  |  | rs326 | 966 | -0.05 | 0.03^*^ | 0.28 |  |
|  |  |  | rs2165558 | 35855 | 0.02 | 0.43 | 0.72 |  |
|  |  |  | rs4523270 | 36134 | 0.02 | 0.42 | 0.71 |  |
|  |  |  | rs12541912 | 39386 | 0.02 | 0.46 | 0.75 |  |
| FADS1-3 | 11q12.2 | rs174546 |  |  | -0.07 | 0.05 | 0.35 |  |
|  |  |  | rs1535 | 28142 | -0.07 | 0.03^*^ | 0.28 |  |
|  |  |  | rs174566 | 22532 | -0.03 | 0.24 | 0.55 |  |
|  |  |  | rs174576 | 33680 | -0.01 | 0.58 | 0.84 |  |
|  |  |  | rs174577 | 34984 | 0.001 | 0.96 | 0.99 |  |
|  |  |  | rs102275 | 12027 | -0.02 | 0.52 | 0.79 |  |
|  |  |  | rs174535 | 18474 | -0.03 | 0.29 | 0.59 |  |
|  |  |  | rs174583 | 39920 | -0.01 | 0.71 | 0.91 |  |
| CCDC92/DNAH10/ZNF664 | 12q24.31.B | rs7307277 |  |  | 0.06 | 0.01^*^ | 0.16 |  |
|  |  |  | rs7311969 | 4823 | -0.04 | 0.09 | 0.43 |  |
|  |  |  | rs1187415 | 16373 | -0.04 | 0.04^*^ | 0.31 |  |
|  |  |  | rs12823740 | 17154 | -0.03 | 0.26 | 0.57 |  |
|  |  |  | rs7973683 | 25933 | -0.04 | 0.08 | 0.40 |  |
|  |  |  | rs4765219 | 35046 | 0.06 | 0.009 | 0.15 |  |
|  |  |  | rs7312404 | 28428 | 0.06 | 0.006 | 0.14 |  |
|  |  |  | rs4930726 | 46825 | -0.05 | 0.02^*^ | 0.20 |  |
|  |  |  | rs11057396 | 56094 | 0.06 | 0.01^*^ | 0.16 |  |
|  |  |  | rs12298484 | 56482 | 0.05 | 0.02^*^ | 0.23 |  |
|  |  |  | rs10846580 | 59703 | -0.03 | 0.18 | 0.45 |  |
|  |  |  | rs9971695 | 61665 | -0.04 | 0.04^*^ | 0.30 |  |
|  |  |  | rs3802999 | 61058 | 0.05 | 0.02^*^ | 0.20 |  |
|  |  |  | rs11057397 | 55428 | 0.05 | 0.02^*^ | 0.20 |  |
|  |  |  | rs7305864 | 33276 | 0.05 | 0.02^*^ | 0.20 |  |
|  |  |  | rs6488914 | 27315 | 0.06 | 0.008^*^ | 0.14 |  |
|  |  |  | rs2178663 | 41251 | 0.06 | 0.006^*^ | 0.14 |  |
|  |  |  | rs11057401 | 47850 | 0.06 | 0.007^*^ | 0.14 |  |
|  |  |  | rs4930721 | 57267 | 0.06 | 0.02^*^ | 0.19 |  |
|  |  |  | rs1054852 | 21160 | -0.04 | 0.05 | 0.35 |  |
| LIPC | 15q22.1 | rs1800588 |  |  | 0.11 | 2.46*10^-6*^ | 7.4*10^-4*^ |  |
| CETP | 16q13 | rs1800775 |  |  | -0.08 | 4.06*10^-4*^ | 0.03* |  |
| LIPG | 18q21.1 | rs4939883 |  |  | -0.02 | 0.39 | 0.70 |  |
|  |  |  | rs10438978 | 9028 | -0.03 | 0.11 | 0.44 |  |
|  |  |  | rs7239867 | 2497 | 0.02 | 0.66 | 0.87 |  |
|  |  |  | rs7240405 | 8124 | -0.009 | 0.79 | 0.93 |  |
|  |  |  | rs2156552 | 14454 | 0.02 | 0.73 | 0.92 |  |
|  |  |  | rs1943973 | 12302 | -0.002 | 0.96 | 0.99 |  |
| PLTP | 20q13.12.B | rs6065904 |  |  | -0.11 | 1.28*10^-4*^ | 0.02* |  |
| TIMD4\|HAVCR1 | 5q33.3 | rs6873053 |  |  | -0.21 | 0.02^*^ | 0.23 |  |
| LCAT | 16q22.1 | rs2271293 |  |  | 0.05 | 0.24 | 0.54 |  |
|  |  |  | rs1124324 | 4583 | 0.04 | 0.10 | 0.44 |  |
|  |  |  | rs3809630 | 22670 | 0.05 | 0.08 | 0.41 |  |
|  |  |  | rs16942887 | 25972 | 0.05 | 0.06 | 0.36 |  |
|  |  |  | rs4474673 | 143292 | 0.05 | 0.07 | 0.38 |  |
|  |  |  | rs2292318 | 83636 | 0.06 | 0.03^*^ | 0.28 |  |
| MAFB | 20q12 | rs2865879 |  |  | -0.02 | 0.52 | 0.79 |  |
|  |  |  | rs2903114 | 6059 | -0.02 | 0.56 | 0.83 |  |
|  |  |  | rs761564 | 9618 | -0.12 | 0.006^*^ | 0.14 |  |
|  |  |  | rs2050204 | 11427 | -0.11 | 0.01^*^ | 0.16 |  |
|  |  |  | rs2425444 | 18918 | -0.01 | 0.60 | 0.85 |  |
|  |  |  | rs2425425 | 37897 | -0.01 | 0.70 | 0.90 |  |
|  |  |  | rs2425451 | 15985 | -0.03 | 0.38 | 0.69 |  |
|  |  |  | rs3091957 | 39042 | -0.02 | 0.55 | 0.82 |  |
|  |  |  | rs2425421 | 39988 | -0.01 | 0.72 | 0.91 |  |
| HDL Medium | |  |  |  |  |  |  |  |
| APOA2 | 1q23.3 | rs4073054 |  |  | -0.12 | 0.001^**^ | 0.06 |  |
| FADS1-3 | 11q12.2 | rs174537 |  |  | 0.05 | 0.37 | 0.68 |  |
|  |  |  | rs174535 | 1324 | 0.05 | 0.18 | 0.45 |  |
|  |  |  | rs102275 | 5123 | 0.04 | 0.33 | 0.63 |  |
|  |  |  | rs1535 | 45292 | 0.05 | 0.29 | 0.59 |  |
|  |  |  | rs174566 | 39682 | 0.04 | 0.24 | 0.54 |  |
|  |  |  | rs174576 | 50830 | -0.007 | 0.85 | 0.95 |  |
|  |  |  | rs174577 | 52134 | 0.009 | 0.79 | 0.93 |  |
|  |  |  | rs174583 | 57070 | 6.97*10^-4^ | 0.98 | 1.00 |  |
|  |  |  | rs108499 | 5443 | 0.05 | 0.38 | 0.69 |  |
| APOC1-APOE | 19q13.32 | rs405509 |  |  | -0.15 | 1.57*10^-4*^ | 0.02* |  |
| *TIMD4\|HAVCR1* | 5q33.3 | rs4704810 |  |  | 0.006 | 0.92 | 0.97 |  |
| HDL Small | |  |  |  |  |  |  |  |
| GCKR | 2p23.3 | rs780094 |  |  | 0.25 | 0.30 | 0.59 |  |
| APOA1-A5 | 11q23.3 | rs518181 |  |  | 0.04 | 0.88 | 0.97 |  |
|  |  |  | rs7120706 | 57300 | 0.14 | 0.59 | 0.84 |  |
|  |  |  | rs7120963 | 57474 | 0.07 | 0.78 | 0.92 |  |
|  |  |  | rs10892052 | 61526 | 0.10 | 0.71 | 0.91 |  |
|  |  |  | rs10892053 | 84216 | 0.08 | 0.77 | 0.92 |  |
|  |  |  | rs10892063 | 123368 | -0.09 | 0.76 | 0.92 |  |
|  |  |  | rs11216267 | 179605 | 0.09 | 0.74 | 0.92 |  |
|  |  |  | rs10892072 | 187526 | 0.08 | 0.77 | 0.92 |  |
|  |  |  | rs7120515 | 219139 | -0.12 | 0.57 | 0.84 |  |
| LIPC | 15q22.1 | rs1800588 |  |  | -0.33 | 0.11 | 0.44 |  |
| PLTP | 20q13.12.B | rs4810479 |  |  | 1.15 | 2.21*10^-8*^ | 1*10^-5^* |  |
| *LCAT* | 16q22.1 | rs1109166 |  |  | 0.16 | 0.43 | 0.72 |  |
|  |  |  | rs7199443 | 136253 | 0.18 | 0.35 | 0.65 |  |
| HDL total | |  |  |  |  |  |  |  |
| GCKR | 2p23.3 | rs1260326 |  |  | 0.49 | 0.12 | 0.44 |  |
| APOA1-A5 | 11q23.3 | rs518181 |  |  | -0.15 | 0.61 | 0.85 |  |
|  |  |  | rs7120706 | 57300 | 0.02 | 0.96 | 0.99 |  |
|  |  |  | rs7120963 | 57474 | 0.03 | 0.93 | 0.98 |  |
|  |  |  | rs10892052 | 61526 | -0.05 | 0.86 | 0.96 |  |
|  |  |  | rs10892053 | 84216 | -0.08 | 0.80 | 0.93 |  |
|  |  |  | rs10892063 | 123368 | -0.03 | 0.92 | 0.97 |  |
|  |  |  | rs11216267 | 179605 | -0.10 | 0.74 | 0.92 |  |
|  |  |  | rs10892072 | 187526 | -0.09 | 0.76 | 0.92 |  |
|  |  |  | rs7120515 | 219139 | -0.16 | 0.50 | 0.78 |  |
| CETP | 16q13 | rs7499892 |  |  | -0.99 | 1.77*10^-4*^ | 0.02* |  |
| PLTP | 20q13.12.B | rs6065906 |  |  | 0.46 | 0.12 | 0.44 |  |
|  |  |  | rs7679 | 22487 | -0.17 | 0.73 | 0.92 |  |
|  |  |  | rs6073972 | 36283 | -0.22 | 0.66 | 0.87 |  |
| *LCAT* | 16q22.1 | rs1109166 |  |  | -0.06 | 0.78 | 0.92 |  |
|  |  |  | rs7199443 | 136253 | -0.04 | 0.87 | 0.97 |  |
| *MAFB* | 20q12 | rs6016408 |  |  | -0.15 | 0.55 | 0.82 |  |
|  |  |  | rs6016400 | 11005 | -0.46 | 0.07 | 0.38 |  |
|  |  |  | rs735031 | 13417 | -0.45 | 0.10 | 0.44 |  |
|  |  |  | rs6029251 | 14697 | -0.48 | 0.08 | 0.41 |  |
| SBF2 | 11p15.4 | rs7938647 |  |  | -0.99 | 0.03^*^ | 0.25 |  |
|  |  |  | rs11042595 | 1310 | -0.79 | 0.04 | 0.28 |  |
|  |  |  | rs11042623 | 55911 | -0.69 | 0.07 | 0.38 |  |
|  |  |  | rs7938491 | 109969 | -0.31 | 0.32 | 0.62 |  |
|  |  |  | rs7935934 | 139762 | -0.49 | 0.18 | 0.45 |  |
|  |  |  | rs1822292 | 183255 | -0.60 | 0.12 | 0.44 |  |
|  |  |  | rs10840327 | 109455 | -0.99 | 0.02^*^ | 0.20 |  |
|  |  |  | rs4910081 | 119572 | -0.94 | 0.03^*^ | 0.28 |  |
|  |  |  | rs7114303 | 34264 | 0.03 | 0.90 | 0.97 |  |
|  |  |  | rs1372807 | 76876 | -0.18 | 0.50 | 0.78 |  |
|  |  |  | rs4909917 | 80258 | -0.10 | 0.72 | 0.91 |  |
|  |  |  | rs4576815 | 74195 | -0.27 | 0.37 | 0.68 |  |
|  |  |  | rs4910086 | 75443 | -0.13 | 0.64 | 0.87 |  |
|  |  |  | rs7104093 | 277711 | -0.96 | 0.05 | 0.31 |  |
|  |  |  | rs11042543 | 121605 | -1.00 | 0.01^*^ | 0.18 |  |
| VLDL diameter | |  |  |  |  |  |  |  |
| APOB | 2p24.1 | rs676210 |  |  | 0.03 | 0.25 | 0.55 |  |
|  |  |  | rs6754295 | 25341 | 0.02 | 0.34 | 0.64 |  |
| GCKR | 2p23.3 | rs1260326 |  |  | -0.007 | 0.77 | 0.92 |  |
| intergenic, PPP1R3B | 8p23.1 | rs983309 |  |  | 0.004 | 0.83 | 0.95 |  |
| LDL diameter | |  |  |  |  |  |  |  |
| GCKR | 2p23.3 | rs780094 |  |  | -0.002 | 0.92 | 0.97 |  |
| LPL | 8p21.3 | rs2083637 |  |  | 0.005 | 0.82 | 0.94 |  |
|  |  |  | rs765547 | 1099 | -0.01 | 0.66 | 0.87 |  |
|  |  |  | rs1919484 | 4501 | -0.008 | 0.73 | 0.92 |  |
|  |  |  | rs17411126 | 9903 | 9.89*10^-5^ | 1.00 | 1.00 |  |
|  |  |  | rs4922117 | 12589 | 0.001 | 0.96 | 0.99 |  |
|  |  |  | rs17489282 | 12657 | 1.41*10^-4^ | 0.99 | 1.00 |  |
|  |  |  | rs17411045 | 12813 | 1.38*10^-4^ | 0.99 | 1.00 |  |
|  |  |  | rs17411031 | 12865 | 1.39*10^-4^ | 0.99 | 1.00 |  |
|  |  |  | rs17489268 | 13130 | -0.002 | 0.94 | 0.98 |  |
|  |  |  | rs327 | 45639 | 0.03 | 0.07 | 0.39 |  |
|  |  |  | rs331 | 44770 | 0.03 | 0.10 | 0.44 |  |
|  |  |  | rs301 | 48241 | NA | NA | NA |  |
|  |  |  | rs297 | 48804 | 0.03 | 0.15 | 0.44 |  |
|  |  |  | rs3916027 | 40307 | 0.03 | 0.09 | 0.43 |  |
|  |  |  | rs12541912 | 5384 | 0.006 | 0.76 | 0.92 |  |
|  |  |  | rs4523270 | 8636 | -0.004 | 0.84 | 0.95 |  |
|  |  |  | rs2165558 | 8915 | -0.004 | 0.84 | 0.95 |  |
|  |  |  | rs15285 | 40508 | 0.04 | 0.03^*^ | 0.28 |  |
|  |  |  | rs11986942 | 2270 | -0.03 | 0.13 | 0.44 |  |
|  |  |  | rs13702 | 40683 | 0.04 | 0.04^*^ | 0.28 |  |
|  |  |  | rs326 | 45736 | -0.04 | 0.03^*^ | 0.27 |  |
|  |  |  | rs1441766 | 2387 | 0.02 | 0.18 | 0.45 |  |
| APOA1-A5 | 11q23.3 | rs3135506 |  |  | -0.08 | 0.05^*^ | 0.32 |  |
| LIPC | 15q22.1 | rs1800588 |  |  | 0.06 | 0.001^*^ | 0.06 |  |
| CETP | 16q13 | rs1800775 |  |  | -0.03 | 0.11 | 0.44 |  |
| LIPG | 18q21.1 | rs4939883 |  |  | -0.01 | 0.48 | 0.76 |  |
|  |  |  | rs10438978 | 9028 | -0.03 | 0.06 | 0.35 |  |
|  |  |  | rs7239867 | 2497 | -0.03 | 0.46 | 0.75 |  |
|  |  |  | rs7240405 | 8124 | -0.01 | 0.60 | 0.85 |  |
|  |  |  | rs2156552 | 14454 | -0.03 | 0.48 | 0.76 |  |
|  |  |  | rs1943973 | 12302 | -0.05 | 0.27 | 0.58 |  |
| PLTP | 20q13.12.B | rs6065906 |  |  | -0.06 | 0.01^*^ | 0.16 |  |
|  |  |  | rs7679 | 22487 | -0.07 | 0.06 | 0.36 |  |
|  |  |  | rs6073972 | 36283 | -0.09 | 0.03^*^ | 0.27 |  |
| TIMD4\|HAVCR1 | 5q33.3 | rs7706174 |  |  | -0.03 | 0.13 | 0.44 |  |
|  |  |  | rs6878732 | 10142 | 0.03 | 0.07 | 0.38 |  |
|  |  |  | rs10059699 | 9244 | -0.05 | 0.007^*^ | 0.14 |  |
| LCAT | 16q22.1 | rs7200210 |  |  | 0.005 | 0.78 | 0.92 |  |
|  |  |  | rs6499141 | 184762 | 0.02 | 0.36 | 0.67 |  |
|  |  |  | rs7200950 | 292144 | 0.01 | 0.46 | 0.75 |  |
|  |  |  | rs7195415 | 292839 | 0.01 | 0.46 | 0.75 |  |
|  |  |  | rs9972635 | 299316 | 0.03 | 0.16 | 0.44 |  |
| MAFB | 20q12 | rs2865892 |  |  | -0.006 | 0.80 | 0.93 |  |
|  |  |  | rs2206665 | 5724 | 0.01 | 0.63 | 0.87 |  |
| HDL diameter | |  |  |  |  |  |  |  |
| GCKR | 2p23.3 | rs780094 |  |  | 0.006 | 0.75 | 0.92 |  |
| LPL | 8p21.3 | rs331 |  |  | 0.02 | 0.35 | 0.65 |  |
|  |  |  | rs17489268 | 31640 | 0.01 | 0.59 | 0.84 |  |
|  |  |  | rs17411031 | 31905 | 0.01 | 0.60 | 0.85 |  |
|  |  |  | rs17411045 | 31957 | 0.01 | 0.60 | 0.85 |  |
|  |  |  | rs17489282 | 32113 | 0.01 | 0.60 | 0.85 |  |
|  |  |  | rs4922117 | 32181 | 0.01 | 0.61 | 0.85 |  |
|  |  |  | rs17411126 | 34867 | 0.01 | 0.58 | 0.84 |  |
|  |  |  | rs2083637 | 44770 | 0.01 | 0.56 | 0.83 |  |
|  |  |  | rs765547 | 45869 | 0.003 | 0.91 | 0.97 |  |
|  |  |  | rs1919484 | 49271 | 0.008 | 0.74 | 0.92 |  |
|  |  |  | rs15285 | 4262 | 0.006 | 0.70 | 0.90 |  |
|  |  |  | rs13702 | 4087 | 0.006 | 0.69 | 0.89 |  |
|  |  |  | rs326 | 966 | -0.02 | 0.14 | 0.44 |  |
|  |  |  | rs2165558 | 35855 | -0.007 | 0.70 | 0.90 |  |
|  |  |  | rs4523270 | 36134 | -0.007 | 0.72 | 0.91 |  |
|  |  |  | rs12541912 | 39386 | 0.002 | 0.93 | 0.98 |  |
| FADS1-3 | 11q12.2 | rs1535 |  |  | -0.04 | 0.09 | 0.43 |  |
|  |  |  | rs174568 | 4156 | -0.03 | 0.31 | 0.61 |  |
|  |  |  | rs174546 | 28142 | -0.03 | 0.29 | 0.59 |  |
|  |  |  | rs174576 | 5538 | -0.002 | 0.90 | 0.97 |  |
|  |  |  | rs174566 | 5610 | -0.02 | 0.38 | 0.69 |  |
|  |  |  | rs174577 | 6842 | 0.009 | 0.58 | 0.84 |  |
|  |  |  | rs174537 | 45292 | -0.03 | 0.32 | 0.62 |  |
|  |  |  | rs174583 | 11778 | -8.93*10^-4^ | 0.96 | 0.99 |  |
|  |  |  | rs102275 | 40169 | -0.02 | 0.24 | 0.54 |  |
|  |  |  | rs174535 | 46616 | -0.004 | 0.84 | 0.95 |  |
|  |  |  | rs4246215 | 33673 | -0.03 | 0.27 | 0.58 |  |
|  |  |  | rs174556 | 17337 | -0.04 | 0.11 | 0.44 |  |
|  |  |  | rs174555 | 18212 | -0.04 | 0.11 | 0.44 |  |
|  |  |  | rs174549 | 26590 | -0.05 | 0.11 | 0.44 |  |
| LIPC | 15q22.1 | rs1800588 |  |  | 0.07 | 9.11*10^-6**^ | 0.002* |  |
| CETP | 16q13 | rs1800775 |  |  | -0.05 | 0.001^**^ | 0.06 |  |
| LIPG | 18q21.1 | rs4939883 |  |  | -0.01 | 0.41 | 0.71 |  |
|  |  |  | rs10438978 | 9028 | -0.03 | 0.07 | 0.39 |  |
|  |  |  | rs7239867 | 2497 | -0.02 | 0.64 | 0.87 |  |
|  |  |  | rs7240405 | 8124 | -0.02 | 0.52 | 0.79 |  |
|  |  |  | rs2156552 | 14454 | -0.007 | 0.86 | 0.96 |  |
|  |  |  | rs1943973 | 12302 | -0.006 | 0.89 | 0.97 |  |
| PLTP | 20q13.12.B | rs6065906 |  |  | -0.05 | 0.01^*^ | 0.16 |  |
|  |  |  | rs7679 | 22487 | -0.06 | 0.12 | 0.44 |  |
|  |  |  | rs6073972 | 36283 | -0.06 | 0.08 | 0.42 |  |
| LCAT | 16q22.1 | rs4986970 |  |  | 0.04 | 0.72 | 0.92 |  |
| MAFB | 20q12 | rs2865892 |  |  | -0.03 | 0.13 | 0.44 |  |
|  |  |  | rs2206665 | 5724 | -0.02 | 0.36 | 0.67 |  |

^a^ models are adjusted for age, gender, BMI, current smoking status, study centers and Principle Components (PC1-PC4)

^*^ significant p-value or Q-value results with asterisk

**S 5 Table** SNP-phenotype associations using proxy SNPS in the Hispanic American population of MESA

| Genes | Chr | | SNPs | Proxy SNPs | distance | Hispanic Americans | | |
| --- | --- | --- | --- | --- | --- | --- | --- | --- |
|  |  |  |  |  |  | beta | P | Q |
| VLDL Large | | |  |  |  |  |  |  |
| GCKR | 2p23.3 | | rs1260326 |  |  | 0.16 | 7.63*10^-4*^ | 0.008* |
| LPL | 8p21.3 | | rs1059611 |  |  | -0.11 | 0.12 | 0.36 |
|  |  | |  | rs12682115 | 22497 | -0.14 | 0.06 | 0.21 |
|  |  | |  | rs17411024 | 27571 | -0.10 | 0.13 | 0.36 |
| APOA1-A5 | 11q23.3 | | rs3135506 |  |  | 0.32 | 2.13*10^-6*^ | 5*10^-5*^ |
| APOC1-APOE | 19q13.32 | | rs439401 |  |  | 0.002 | 0.97 | 0.97 |
| TIMD4\|HAVCR1 | 5q33.3 | | rs1354163 |  |  | -0.19 | 0.03^*^ | 0.12 |
|  |  | |  | rs1948759 | 21954 | -0.08 | 0.23 | 0.49 |
|  |  | |  | rs905211 | 26515 | -0.08 | 0.25 | 0.51 |
|  |  | |  | rs1393209 | 56837 | -0.13 | 0.13 | 0.36 |
|  |  | |  | rs1553316 | 58806 | -0.12 | 0.11 | 0.35 |
|  |  | |  | rs1553317 | 58721 | -0.13 | 0.10 | 0.32 |
| LCAT | 16q22.1 | | rs1109166 |  |  | -0.02 | 0.75 | 0.90 |
|  |  | |  | rs8060686 | 65865 | -0.01 | 0.82 | 0.92 |
|  |  | |  | rs9928531 | 144211 | -0.02 | 0.78 | 0.92 |
|  |  | |  | rs12449157 | 268485 | 0.01 | 0.86 | 0.94 |
| MAFB | 20q12 | | rs1076759 |  |  | 0.01 | 0.83 | 0.93 |
| VLDL Medium | | |  |  |  |  |  |  |
| ANGPTL3 | 1p31.3 | | rs10889353 |  |  | -0.15 | 0.03^*^ | 0.15 |
|  |  | |  | rs1168040 | 157621 | -0.14 | 0.04 | 0.17 |
|  |  | |  | rs1168113 | 37902 | -0.16 | 0.02 | 0.12 |
| APOB | 2p24.1 | | rs673548 |  |  | -0.07 | 0.34 | 0.58 |
| GCKR | 2p23.3 | | rs1260326 |  |  | 0.17 | 0.03^*^ | 0.12 |
| MLXIPL | 7q11.23 | | rs2240466 |  |  | -0.36 | 0.01^*^ | 0.06 |
|  |  | |  | rs17145750 | 170109 | -0.25 | 0.04 | 0.17 |
| LPL | 8p21.3 | | rs1059611 |  |  | -0.50 | 1.93*10^-5*^ | 3.8*10^-4^* |
| APOA1-A5 | 11q23.3 | | rs3135506 |  |  | 0.52 | 1.35*10^-6**^ | 4*10^-5^* |
| JMJD1C | 10q21.3 | | rs12768534 |  |  | -0.05 | 0.48 | 0.73 |
|  |  | |  | rs10822184 | 3744 | 0.02 | 0.71 | 0.87 |
|  |  | |  | rs10822186 | 9486 | 0.03 | 0.68 | 0.85 |
| TIMD4\|HAVCR1 | 5q33.3 | | rs1354163 |  |  | -0.45 | 7.11*10^-4**^ | 0.008* |
| LCAT | 16q22.1 | | rs255052 |  |  | -0.01 | 0.91 | 0.95 |
|  |  | |  | rs255048 | 12146 | -0.01 | 0.92 | 0.95 |
| MAFB | 20q12 | | rs2865892 |  |  | -0.16 | 0.08 | 0.26 |
| VLDL Small | | |  |  |  |  |  |  |
| PCSK9 | 1p32.3 | | rs11591147 |  |  | -0.15 | 0.76 | 0.90 |
| ANGPTL3 | 1p31.3 | | rs1167998 |  |  | -0.15 | 0.02^*^ | 0.11 |
|  |  | |  | rs1168040 | 28943 | -0.16 | 0.02^*^ | 0.10 |
|  |  | |  | rs1168113 | 224466 | -0.15 | 0.02^*^ | 0.12 |
| CELSR2/PSRC1/SPRT1 | 1p13.3 | | rs646776 |  |  | -0.15 | 0.04^*^ | 0.17 |
| APOB | 2p24.1 | | rs6754295 |  |  | -0.30 | 2.84*10^-5*^ | 5*10^-4^* |
| LPL | 8p21.3 | | rs328 |  |  | -0.16 | 0.19 | 0.44 |
| ABO | 9q34.2 | | rs507666 |  |  | 0.16 | 0.09 | 0.28 |
| APOA1-A5 | 11q23.3 | | rs3135506 |  |  | 0.15 | 0.14 | 0.37 |
| CETP | 16q13 | | rs1800775 |  |  | -0.14 | 0.04^*^ | 0.16 |
| LDLR | 19p13.2 | | rs6511720 |  |  | 0.06 | 0.71 | 0.87 |
|  |  | |  | rs2228671 | 8606 | 0.19 | 0.20 | 0.47 |
| APOC1-APOE | 19q13.32 | | rs439401 |  |  | -0.11 | 0.20 | 0.45 |
| TIMD4\|HAVCR1 | 5q33.3 | | rs1363232 |  |  | -0.10 | 0.18 | 0.43 |
|  |  | |  | rs6874202 | 8206 | -0.13 | 0.06 | 0.23 |
|  |  | |  | rs12657266 | 12581 | -0.14 | 0.05 | 0.19 |
|  |  | |  | rs1501908 | 14747 | -0.14 | 0.04 | 0.18 |
| LCAT | 16q22.1 | | rs255052 |  |  | 0.04 | 0.64 | 0.82 |
|  |  | |  | rs255048 | 12416 | 0.06 | 0.54 | 0.78 |
| MAFB | 20q12 | | rs6102085 |  |  | -0.02 | 0.81 | 0.92 |
| VLDL Total | | |  |  |  |  |  |  |
| PCSK9 | 1p32.3 | | rs11591147 |  |  | -0.13 | 0.82 | 0.92 |
| ANGPTL3 | 1p31.3 | | rs10889353 |  |  | -0.22 | 0.005^*^ | 0.04* |
| APOB | 2p24.1 | | rs673548 |  |  | -0.25 | 0.004^*^ | 0.03* |
| GCKR | 2p23.3 | | rs780094 |  |  | 0.21 | 0.01^*^ | 0.06 |
| LPL | 8p21.3 | | rs328 |  |  | -0.61 | 1.54*10^-5*^ | 3.3*10^-4^* |
| APOA1-A5 | 11q23.3 | | rs3135506 |  |  | 0.56 | 2.98*10^-6*^ | 7*10^-5^* |
| CETP | 16q13 | | rs1800775 |  |  | 0.01 | 0.89 | 0.95 |
| APOC1-APOE | 19q13.32 | | rs439401 |  |  | -0.09 | 0.39 | 0.63 |
| TIMD4\|HAVCR1 | 5q33.3 | | rs1354163 |  |  | -0.51 | 5.22*10^-4*^ | 0.006* |
| LCAT | 16q22.1 | | rs255052 |  |  | 0.03 | 0.81 | 0.92 |
|  |  | |  | rs255048 | 12416 | 0.04 | 0.73 | 0.88 |
| MAFB | 20q12 | | rs2865892 |  |  | -0.19 | 0.06 | 0.21 |
| JMJD1C | 10q21.3 | | rs7923609 |  |  | -0.03 | 0.73 | 0.88 |
|  |  | |  | rs10822184 | 203331 | 0.04 | 0.61 | 0.81 |
|  |  | |  | rs10822186 | 216561 | 0.04 | 0.56 | 0.79 |
|  |  | |  | rs4746203 | 190175 | -0.04 | 0.60 | 0.81 |
|  |  | |  | rs3847326 | 198037 | -0.04 | 0.57 | 0.79 |
|  |  | |  | rs10761785 | 184944 | NA | NA | NA |
| IDL total | | |  |  |  |  |  |  |
| GCKR | 2p23.3 | | rs780094 |  |  | 0.11 | 0.56 | 0.79 |
| LIPC | 15q22.1 | | rs1532085 |  |  | 0.50 | 0.005^*^ | 0.04* |
| CETP | 16q13 | | rs1800775 |  |  | 0.36 | 0.05 | 0.18 |
| *TIMD4\|HAVCR1* | 5q33.3 | | rs1363232 |  |  | -0.05 | 0.80 | 0.92 |
|  |  | |  | rs6874202 | 8206 | -0.26 | 0.17 | 0.42 |
|  |  | |  | rs12657266 | 12581 | -0.27 | 0.14 | 0.38 |
|  |  | |  | rs1501908 | 14747 | -0.28 | 0.13 | 0.37 |
| *LCAT* | 16q22.1 | | rs255049 |  |  | -0.29 | 0.15 | 0.39 |
| LDL Large | | |  |  |  |  |  |  |
| PCSK9 | 1p32.3 | | rs11591147 |  |  | -33.00 | 0.65 | 0.83 |
| APOB | 2p24.1 | | rs1713222 |  |  | -38.72 | 0.007^*^ | 0.05 |
| HMGCR | 5q13.3 | | rs5744680 |  |  | -8.60 | 0.38 | 0.63 |
|  |  | |  | rs3846662 | 228806 | 9.64 | 0.32 | 0.57 |
|  |  | |  | rs12654264 | 231287 | -1.23 | 0.90 | 0.95 |
|  |  | |  | rs11749783 | 253808 | -1.72 | 0.87 | 0.94 |
|  |  | |  | rs7703051 | 254403 | -1.74 | 0.86 | 0.94 |
|  |  | |  | rs3846663 | 224164 | -3.64 | 0.72 | 0.88 |
|  |  | |  | rs2335418 | 276411 | 0.32 | 0.97 | 0.98 |
|  |  | |  | rs11957260 | 271158 | 4.70 | 0.63 | 0.81 |
| LPL | 8p21.3 | | rs2083637 |  |  | 23.86 | 0.04^*^ | 0.17 |
|  |  | |  | rs327 | 45639 | 21.82 | 0.05 | 0.18 |
|  |  | |  | rs331 | 44770 | 25.97 | 0.02^*^ | 0.11 |
|  |  | |  | rs301 | 48241 | NA | NA | NA |
|  |  | |  | rs297 | 48804 | 30.80 | 0.01^*^ | 0.07 |
|  |  | |  | rs12541912 | 5384 | 12.78 | 0.25 | 0.51 |
|  |  | |  | rs4523270 | 8636 | 12.11 | 0.28 | 0.55 |
|  |  | |  | rs2165558 | 8915 | 11.57 | 0.30 | 0.57 |
|  |  | |  | rs15285 | 40508 | 18.44 | 0.08 | 0.28 |
|  |  | |  | rs11986942 | 2270 | 14.81 | 0.16 | 0.40 |
|  |  | |  | rs13702 | 40683 | 18.11 | 0.09 | 0.29 |
|  |  | |  | rs326 | 45736 | 23.29 | 0.03 | 0.13 |
|  |  | |  | rs1441766 | 2387 | 5.86 | 0.58 | 0.79 |
| FADS1-3 | 11q12.2 | | rs1535 |  |  | 28.53 | 0.006^*^ | 0.05 |
| LIPC | 15q22.1 | | rs1800588 |  |  | 59.47 | 8.5*10^-9*^ | 5*10^-7*^ |
| CETP | 16q13 | | rs1864163 |  |  | -50.34 | 3.58*10^-5*^ | 5.7*10^-4*^ |
| LIPG | 18q21.1 | | rs8090363 |  |  | -13.52 | 0.20 | 0.45 |
| LDLR | 19p13.2 | | rs6511720 |  |  | 13.63 | 0.54 | 0.78 |
|  |  | |  | rs2228671 | 8606 | 27.54 | 0.25 | 0.51 |
| APOC1-APOE | 19q13.32 | | rs4803750 |  |  | -6.98 | 0.62 | 0.81 |
|  |  | |  | rs1531517 | 5454 | -32.28 | 0.13 | 0.37 |
| PLTP | 20q13.12.B | | rs4810479 |  |  | -12.45 | 0.21 | 0.46 |
| TIMD4\|HAVCR1 | 5q33.3 | | rs7706174 |  |  | 3.51 | 0.78 | 0.92 |
|  |  | |  | rs6878732 | 10142 | 3.29 | 0.78 | 0.92 |
| MAFB | 20q12 | | rs6102085 |  |  | -0.96 | 0.92 | 0.95 |
| LDL Small | | |  |  |  |  |  |  |
| CELSR2/PSRC1/SPRT1 | 1p13.3 | | rs646776 |  |  | -42.09 | 0.009^*^ | 0.06 |
| GCKR | 2p23.3 | | rs780094 |  |  | -2.70 | 0.86 | 0.94 |
| LPL | 8p21.3 | | rs2083637 |  |  | -55.28 | 0.001^*^ | 0.01^*^ |
| TRIB1 | 8q24.13 | | rs6982636 |  |  | -15.33 | 0.28 | 0.55 |
|  |  | |  | rs10808546 | 16503 | -27.82 | 0.06 | 0.23 |
| APOA1-A5 | 11q23.3 | | rs3135506 |  |  | 54.33 | 0.01^*^ | 0.08 |
| LIPC | 15q22.1 | | rs1800588 |  |  | -56.96 | 1.53*10^-4*^ | 0.002^*^ |
| CETP | 16q13 | | rs1800775 |  |  | 54.18 | 2.96*10^-4*^ | 0.004^*^ |
| APOC1-APOE | 19q13.32 | | rs769449 |  |  | -24.60 | 0.47 | 0.72 |
| PLTP | 20q13.12.B | | rs6065906 |  |  | 13.89 | 0.52 | 0.76 |
| TIMD4\|HAVCR1 | 5q33.3 | | rs7706174 |  |  | -8.87 | 0.63 | 0.81 |
|  |  | |  | rs6878732 | 10142 | 1.69 | 0.92 | 0.95 |
| LCAT | 16q22.1 | | rs7200210 |  |  | -1.70 | 0.95 | 0.96 |
|  |  | |  | rs8061384 | 70022 | 2.94 | 0.91 | 0.95 |
|  |  | |  | rs7200950 | 292144 | 2.60 | 0.93 | 0.95 |
|  |  | |  | rs7195415 | 292839 | 2.58 | 0.93 | 0.95 |
|  |  | |  | rs9972635 | 299316 | 15.24 | 0.58 | 0.79 |
| MAFB | 20q12 | | rs2865892 |  |  | -23.79 | 0.19 | 0.44 |
| LDL Total | | |  |  |  |  |  |  |
| PCSK9 | 1p32.3 | | rs11591147 |  |  | -1.33 | 0.32 | 0.57 |
| CELSR2/PSRC1/SPRT1 | 1p13.3 | | rs646776 |  |  | -1.11 | 3.26*10^-8*^ | 1.7*10^-6*^ |
| APOB | 2p24.1 | | rs312985 |  |  | -0.69 | 0.003^*^ | 0.03^*^ |
| GCKR | 2p23.3 | | rs780094 |  |  | -0.17 | 0.38 | 0.62 |
| TRIB1 | 8q24.13 | | rs6982636 |  |  | -0.27 | 0.13 | 0.37 |
|  |  | |  | rs10808546 | 16503 | -0.46 | 0.02* | 0.09 |
| APOA1-A5 | 11q23.3 | | rs3135506 |  |  | 0.18 | 0.51 | 0.75 |
| CETP | 16q13 | | rs1800775 |  |  | 0.08 | 0.69 | 0.86 |
| LDLR | 19p13.2 | | rs6511720 |  |  | -0.71 | 0.08 | 0.27 |
|  |  | |  | rs2228671 | 8606 | -0.52 | 0.23 | 0.49 |
| APOC1-APOE | 19q13.32 | | rs769449 |  |  | -0.32 | 0.46 | 0.71 |
| TIMD4\|HAVCR1 | 5q33.3 | | rs1363232 |  |  | 0.01 | 0.95 | 0.96 |
|  |  | |  | rs6874202 | 8206 | 0.10 | 0.62 | 0.81 |
|  |  | |  | rs12657266 | 12581 | 0.08 | 0.68 | 0.85 |
|  |  | |  | rs1501908 | 14747 | 0.10 | 0.61 | 0.81 |
| MAFB | 20q12 | | rs2865892 |  |  | -0.35 | 0.12 | 0.36 |
| HDL Large | | |  |  |  |  |  |  |
| LPL | 8p21.3 | | rs331 |  |  | 0.03 | 0.15 | 0.39 |
|  |  | |  | rs2083637 | 44770 | 0.05 | 0.02 | 0.12 |
|  |  | |  | rs15285 | 4262 | 0.03 | 0.17 | 0.41 |
|  |  | |  | rs13702 | 4087 | 0.03 | 0.18 | 0.44 |
|  |  | |  | rs326 | 966 | 0.04 | 0.08 | 0.26 |
|  |  | |  | rs301 | 3471 | NA | NA | NA |
|  |  | |  | rs297 | 4034 | 0.03 | 0.32 | 0.57 |
|  |  | |  | rs2165558 | 35855 | 0.03 | 0.28 | 0.55 |
|  |  | |  | rs4523270 | 36134 | 0.02 | 0.30 | 0.57 |
|  |  | |  | rs12541912 | 39386 | 0.02 | 0.34 | 0.58 |
| FADS1-3 | 11q12.2 | | rs174546 |  |  | 0.08 | 2.33*10^-4*^ | 0.003^*^ |
| CCDC92/DNAH10/ZNF664 | 12q24.31.B | | rs7307277 |  |  | 0.03 | 0.19 | 0.44 |
|  |  | |  | rs10846580 | 59703 | 0.02 | 0.30 | 0.57 |
| LIPC | 15q22.1 | | rs1800588 |  |  | 0.17 | <1*10^-10*^ | <1*10^-10*^ |
| CETP | 16q13 | | rs1800775 |  |  | -0.11 | 7.49*10^-8*^ | 2.73*10^-6*^ |
| LIPG | 18q21.1 | | rs4939883 |  |  | -0.02 | 0.53 | 0.77 |
|  |  | |  | rs10438978 | 9028 | -0.03 | 0.31 | 0.57 |
|  |  | |  | rs7239867 | 2497 | -0.04 | 0.33 | 0.57 |
|  |  | |  | rs7240405 | 8124 | -0.05 | 0.14 | 0.37 |
|  |  | |  | rs2156552 | 14454 | -0.04 | 0.32 | 0.57 |
|  |  | |  | rs1943973 | 12302 | -0.05 | 0.21 | 0.46 |
| PLTP | 20q13.12.B | | rs6065904 |  |  | -0.05 | 0.05 | 0.18 |
| TIMD4\|HAVCR1 | 5q33.3 | | rs6873053 |  |  | -0.008 | 0.91 | 0.95 |
| LCAT | 16q22.1 | | rs2271293 |  |  | 0.02 | 0.46 | 0.71 |
|  |  | |  | rs4474673 | 143292 | 0.03 | 0.38 | 0.63 |
| MAFB | 20q12 | | rs2865879 |  |  | 0.02 | 0.44 | 0.69 |
|  |  | |  | rs761564 | 9618 | 0.03 | 0.30 | 0.57 |
|  |  | |  | rs2050204 | 11427 | 0.03 | 0.30 | 0.57 |
| HDL Medium | | |  |  |  |  |  |  |
| APOA2 | 1q23.3 | | rs4073054 |  |  | -0.04 | 0.29 | 0.56 |
| FADS1-3 | 11q12.2 | | rs174537 |  |  | -0.02 | 0.48 | 0.73 |
|  |  | |  | rs102275 | 5123 | -0.02 | 0.61 | 0.81 |
|  |  | |  | rs174566 | 39682 | -0.03 | 0.40 | 0.64 |
|  |  | |  | rs174576 | 50830 | -0.02 | 0.49 | 0.73 |
|  |  | |  | rs174577 | 52134 | -0.03 | 0.31 | 0.57 |
|  |  | |  | rs174583 | 57070 | -0.03 | 0.32 | 0.57 |
| APOC1-APOE | 19q13.32 | | rs405509 |  |  | -0.01 | 0.72 | 0.88 |
| *TIMD4\|HAVCR1* | 5q33.3 | | rs4704810 |  |  | 0.02 | 0.57 | 0.79 |
| HDL Small | | |  |  |  |  |  |  |
| GCKR | 2p23.3 | | rs780094 |  |  | 0.59 | 0.005^*^ | 0.04^*^ |
| APOA1-A5 | 11q23.3 | | rs518181 |  |  | 0.10 | 0.63 | 0.81 |
| LIPC | 15q22.1 | | rs1800588 |  |  | -0.53 | 0.01^*^ | 0.07 |
| PLTP | 20q13.12.B | | rs4810479 |  |  | 1.21 | 2*10^-9*^ | 1.74*10^-7*^ |
| *LCAT* | 16q22.1 | | rs1109166 |  |  | 0.34 | 0.16 | 0.40 |
|  |  | |  | rs8060686 | 65865 | 0.38 | 0.11 | 0.35 |
|  |  | |  | rs9928531 | 144211 | 0.21 | 0.43 | 0.68 |
|  |  | |  | rs12449157 | 268485 | 0.27 | 0.32 | 0.57 |
| HDL total | | |  |  |  |  |  |  |
| GCKR | 2p23.3 | | rs1260326 |  |  | 0.55 | 0.02 | 0.10 |
| APOA1-A5 | 11q23.3 | | rs518181 |  |  | 0.09 | 0.70 | 0.87 |
| CETP | 16q13 | | rs7499892 |  |  | -0.77 | 0.006^*^ | 0.05 |
| PLTP | 20q13.12.B | | rs6065906 |  |  | 1.03 | 0.002^*^ | 0.01^*^ |
| *LCAT* | 16q22.1 | | rs1109166 |  |  | 0.40 | 0.12 | 0.36 |
|  |  | |  | rs8060686 | 65865 | 0.40 | 0.12 | 0.35 |
|  |  | |  | rs9928531 | 144211 | 0.28 | 0.31 | 0.57 |
|  |  | |  | rs12449157 | 268485 | 0.29 | 0.31 | 0.57 |
| *MAFB* | 20q12 | | rs6016408 |  |  | -0.73 | 0.006^*^ | 0.04^*^ |
| SBF2 | 11p15.4 | | rs7938647 |  |  | 0.18 | 0.53 | 0.77 |
|  |  | |  | rs7935934 | 139762 | 0.31 | 0.26 | 0.53 |
|  |  | |  | rs1822292 | 183255 | 0.36 | 0.19 | 0.44 |
|  |  | |  | rs4910081 | 119572 | -0.03 | 0.90 | 0.95 |
|  |  | |  | rs7114303 | 34264 | 0.00 | 0.99 | 0.99 |
|  |  | |  | rs1372807 | 76876 | 0.08 | 0.73 | 0.88 |
|  |  | |  | rs4909917 | 80258 | -0.05 | 0.81 | 0.92 |
|  |  | |  | rs4576815 | 74195 | -0.04 | 0.88 | 0.95 |
|  |  | |  | rs4910086 | 75443 | -0.08 | 0.71 | 0.87 |
|  |  | |  | rs7104093 | 277711 | 0.39 | 0.19 | 0.44 |
|  |  | |  | rs11042543 | 121605 | -0.05 | 0.86 | 0.94 |
| VLDL diameter | | |  |  |  |  |  |  |
| APOB | | 2p24.1 | rs676210 |  |  | 0.06 | 0.01^*^ | 0.07 |
| GCKR | | 2p23.3 | rs1260326 |  |  | 0.09 | 7.78*10^-5*^ | 0.001^*^ |
| intergenic, PPP1R3B | | 8p23.1 | rs983309 |  |  | 0.05 | 0.05 | 0.19 |
| LDL diameter | | |  |  |  |  |  |  |
| GCKR | 2p23.3 | | rs780094 |  |  | -0.03 | 0.24 | 0.50 |
| LPL | 8p21.3 | | rs2083637 |  |  | 0.08 | 0.002^*^ | 0.02* |
| APOA1-A5 | 11q23.3 | | rs3135506 |  |  | -0.13 | 2.95*10^-5*^ | 5.02*10^-4*^ |
| LIPC | 15q22.1 | | rs1800588 |  |  | 0.11 | 4.39*10^-7*^ | 1.40*10^-5*^ |
| CETP | 16q13 | | rs1800775 |  |  | -0.12 | 6.01*10^-8*^ | 2.55*10^-6*^ |
| LIPG | 18q21.1 | | rs4939883 |  |  | -0.02 | 0.39 | 0.63 |
|  |  | |  | rs10438978 | 9028 | -0.02 | 0.37 | 0.62 |
|  |  | |  | rs7239867 | 2497 | -0.05 | 0.15 | 0.39 |
|  |  | |  | rs7240405 | 8124 | -0.04 | 0.22 | 0.47 |
|  |  | |  | rs2156552 | 14454 | -0.06 | 0.13 | 0.37 |
|  |  | |  | rs1943973 | 12302 | -0.07 | 0.07 | 0.26 |
| PLTP | 20q13.12.B | | rs6065906 |  |  | -0.04 | 0.16 | 0.40 |
| TIMD4\|HAVCR1 | 5q33.3 | | rs7706174 |  |  | 0.01 | 0.62 | 0.81 |
|  |  | |  | rs6878732 | 10142 | -0.004 | 0.89 | 0.95 |
| LCAT | 16q22.1 | | rs7200210 |  |  | 0.02 | 0.56 | 0.79 |
|  |  | |  | rs8061384 | 70022 | 0.02 | 0.61 | 0.81 |
|  |  | |  | rs7200950 | 292144 | 0.01 | 0.81 | 0.92 |
|  |  | |  | rs7195415 | 292839 | 0.01 | 0.81 | 0.92 |
|  |  | |  | rs9972635 | 299316 | 0.004 | 0.92 | 0.95 |
| MAFB | 20q12 | | rs2865892 |  |  | 0.04 | 0.10 | 0.32 |
| HDL diameter | | |  |  |  |  |  |  |
| GCKR | 2p23.3 | | rs780094 |  |  | 0.004 | 0.80 | 0.92 |
| LPL | 8p21.3 | | rs331 |  |  | 0.01 | 0.43 | 0.67 |
|  |  | |  | rs2083637 | 44770 | 0.03 | 0.06 | 0.22 |
|  |  | |  | rs15285 | 4262 | 0.01 | 0.48 | 0.73 |
|  |  | |  | rs13702 | 4087 | 0.01 | 0.51 | 0.75 |
|  |  | |  | rs326 | 966 | 0.01 | 0.42 | 0.67 |
|  |  | |  | rs301 | 3471 | NA | NA | NA |
|  |  | |  | rs297 | 4034 | 0.008 | 0.67 | 0.85 |
|  |  | |  | rs2165558 | 35855 | 0.02 | 0.33 | 0.57 |
|  |  | |  | rs4523270 | 36134 | 0.02 | 0.35 | 0.58 |
|  |  | |  | rs12541912 | 39386 | 0.02 | 0.35 | 0.58 |
| FADS1-3 | 11q12.2 | | rs1535 |  |  | 0.06 | 2.54*10^-4*^ | 0.003^*^ |
| LIPC | 15q22.1 | | rs1800588 |  |  | 0.10 | 3*10^-10*^ | 3.40*10^-8*^ |
| CETP | 16q13 | | rs1800775 |  |  | -0.04 | 0.004^*^ | 0.04^*^ |
| LIPG | 18q21.1 | | rs4939883 |  |  | -0.005 | 0.80 | 0.92 |
|  |  | |  | rs10438978 | 9028 | -0.03 | 0.15 | 0.39 |
|  |  | |  | rs7239867 | 2497 | -0.02 | 0.55 | 0.78 |
|  |  | |  | rs7240405 | 8124 | -0.03 | 0.16 | 0.40 |
|  |  | |  | rs2156552 | 14454 | -0.01 | 0.61 | 0.81 |
|  |  | |  | rs1943973 | 12302 | -0.02 | 0.46 | 0.71 |
| PLTP | 20q13.12.B | | rs6065906 |  |  | -0.03 | 0.20 | 0.45 |
| LCAT | 16q22.1 | | rs4986970 |  |  | -0.01 | 0.92 | 0.95 |
| MAFB | 20q12 | | rs2865892 |  |  | 0.003 | 0.87 | 0.94 |

^a^ models are adjusted for age, gender, BMI, current smoking status, study centers, Mexican/Non-Mexican status and Principle Components (PC1-PC4)

^*^ significant p-value or Q-value results with asterisk

**S 6 Table** SNP-phenotype associations using proxy SNPS in the Chinese American population of MESA

| Genes | Chr | SNPs | Proxy SNPs | distance | Chinese-American | | |  |
| --- | --- | --- | --- | --- | --- | --- | --- | --- |
|  |  |  |  |  | beta | P | Q |  |
| VLDL Large | |  |  |  |  |  |  |  |
| GCKR | 2p23.3 | rs1260326 |  |  | 0.16 | 0.04 | 0.59 |  |
| LPL | 8p21.3 | rs1059611 |  |  | 0.17 | 0.12 | 0.59 |  |
|  |  |  | rs7016880 | 52183 | 0.14 | 0.21 | 0.59 |  |
|  |  |  | rs7816447 | 58387 | 0.14 | 0.23 | 0.59 |  |
| APOA1-A5 | 11q23.3 | rs3135506 |  |  | -4.36 | 0.82 | 0.90 |  |
| APOC1-APOE | 19q13.32 | rs439401 |  |  | 0.05 | 0.54 | 0.73 |  |
| TIMD4\|HAVCR1 | 5q33.3 | rs1354163 |  |  | 0.10 | 0.84 | 0.91 |  |
| LCAT | 16q22.1 | rs1109166 |  |  | -0.12 | 0.34 | 0.60 |  |
|  |  |  | rs6499145 | 47781 | 0.35 | 0.17 | 0.59 |  |
|  |  |  | rs10468274 | 55040 | 0.26 | 0.23 | 0.59 |  |
|  |  |  | rs8060686 | 65865 | 0.27 | 0.21 | 0.59 |  |
|  |  |  | rs11553287 | 71975 | 0.36 | 0.16 | 0.59 |  |
|  |  |  | rs13334918 | 76039 | 0.36 | 0.15 | 0.59 |  |
|  |  |  | rs7198357 | 92763 | 0.35 | 0.15 | 0.59 |  |
|  |  |  | rs6499143 | 103118 | 0.38 | 0.14 | 0.59 |  |
|  |  |  | rs7199443 | 136253 | 0.44 | 0.10 | 0.59 |  |
|  |  |  | rs9928531 | 144211 | 0.45 | 0.09 | 0.59 |  |
|  |  |  | rs28563117 | 195252 | 0.46 | 0.10 | 0.59 |  |
|  |  |  | rs12449157 | 268485 | 0.48 | 0.10 | 0.59 |  |
| MAFB | 20q12 | rs1076759 |  |  | 0.09 | 0.28 | 0.59 |  |
| VLDL Medium | |  |  |  |  |  |  |  |
| ANGPTL3 | 1p31.3 | rs10889353 |  |  | -0.18 | 0.20 | 0.59 |  |
|  |  |  | rs1168089 | 4477 | -0.16 | 0.22 | 0.59 |  |
|  |  |  | rs7539035 | 11429 | -0.16 | 0.20 | 0.59 |  |
|  |  |  | rs6587980 | 27338 | -0.15 | 0.23 | 0.59 |  |
|  |  |  | rs1748195 | 68603 | -0.15 | 0.23 | 0.59 |  |
|  |  |  | rs4329540 | 91172 | -0.15 | 0.23 | 0.59 |  |
|  |  |  | rs1168018 | 117076 | -0.15 | 0.23 | 0.59 |  |
|  |  |  | rs1781195 | 129224 | -0.15 | 0.23 | 0.59 |  |
|  |  |  | rs656297 | 210601 | -0.16 | 0.20 | 0.59 |  |
|  |  |  | rs1168099 | 4663 | -0.17 | 0.18 | 0.59 |  |
|  |  |  | rs10889347 | 42370 | -0.16 | 0.22 | 0.59 |  |
|  |  |  | rs6678483 | 43754 | -0.16 | 0.22 | 0.59 |  |
|  |  |  | rs6675401 | 44221 | -0.16 | 0.22 | 0.59 |  |
|  |  |  | rs10789117 | 45931 | -0.16 | 0.22 | 0.59 |  |
|  |  |  | rs1168013 | 121358 | -0.15 | 0.23 | 0.59 |  |
|  |  |  | rs6690733 | 57885 | -0.15 | 0.23 | 0.59 |  |
|  |  |  | rs1748201 | 72690 | -0.15 | 0.23 | 0.59 |  |
|  |  |  | rs10889337 | 137589 | -0.15 | 0.23 | 0.59 |  |
|  |  |  | rs1168032 | 150449 | -0.15 | 0.23 | 0.59 |  |
|  |  |  | rs631106 | 216389 | -0.17 | 0.20 | 0.59 |  |
|  |  |  | rs637723 | 211468 | -0.15 | 0.23 | 0.59 |  |
|  |  |  | rs624660 | 197808 | -0.16 | 0.22 | 0.59 |  |
|  |  |  | rs583609 | 201400 | -0.16 | 0.22 | 0.59 |  |
|  |  |  | rs11207969 | 206445 | -0.15 | 0.23 | 0.59 |  |
|  |  |  | rs1167998 | 186564 | -0.15 | 0.23 | 0.59 |  |
|  |  |  | rs10889332 | 167338 | -0.14 | 0.28 | 0.59 |  |
|  |  |  | rs11207997 | 56290 | -0.15 | 0.23 | 0.59 |  |
|  |  |  | rs1168040 | 157621 | -0.15 | 0.23 | 0.59 |  |
|  |  |  | rs11208000 | 9822 | -0.16 | 0.22 | 0.59 |  |
| APOB | 2p24.1 | rs673548 |  |  | 0.02 | 0.86 | 0.92 |  |
|  |  |  | rs6754295 | 31361 | -0.07 | 0.57 | 0.73 |  |
|  |  |  | rs6544366 | 33527 | -0.05 | 0.70 | 0.83 |  |
|  |  |  | rs11902417 | 38652 | -0.05 | 0.70 | 0.83 |  |
|  |  |  | rs6728178 | 43615 | -0.05 | 0.69 | 0.83 |  |
| GCKR | 2p23.3 | rs1260326 |  |  | 0.07 | 0.54 | 0.73 |  |
| MLXIPL | 7q11.23 | rs2240466 |  |  | 0.01 | 0.97 | 0.99 |  |
| LPL | 8p21.3 | rs1059611 |  |  | -0.07 | 0.71 | 0.83 |  |
|  |  |  | rs7016880 | 52183 | -0.04 | 0.83 | 0.90 |  |
|  |  |  | rs7816447 | 58387 | -0.05 | 0.81 | 0.89 |  |
| APOA1-A5 | 11q23.3 | rs3135506 |  |  | -8.52 | 0.79 | 0.89 |  |
| JMJD1C | 10q21.3 | rs12768534 |  |  | -0.09 | 0.49 | 0.71 |  |
|  |  |  | rs10822184 | 3744 | 0.06 | 0.61 | 0.77 |  |
|  |  |  | rs10822186 | 9486 | 0.07 | 0.55 | 0.73 |  |
| TIMD4\|HAVCR1 | 5q33.3 | rs1354163 |  |  | 0.51 | 0.52 | 0.73 |  |
| LCAT | 16q22.1 | rs255052 |  |  | 0.001 | 1.00 | 1.00 |  |
|  |  |  | rs255048 | 12146 | -0.04 | 0.85 | 0.92 |  |
| MAFB | 20q12 | rs2865892 |  |  | 0.05 | 0.68 | 0.82 |  |
| VLDL Small | |  |  |  |  |  |  |  |
| PCSK9 | 1p32.3 | rs11591147 |  |  | 9.82 | 0.05 | 0.59 |  |
| ANGPTL3 | 1p31.3 | rs1167998 |  |  | -0.06 | 0.54 | 0.73 |  |
|  |  |  | rs4350231 | 8972 | -0.15 | 0.15 | 0.59 |  |
|  |  |  | rs3913007 | 10046 | -0.14 | 0.17 | 0.59 |  |
|  |  |  | rs10889333 | 25398 | -0.14 | 0.19 | 0.59 |  |
|  |  |  | rs10889335 | 28469 | -0.16 | 0.13 | 0.59 |  |
|  |  |  | rs12116574 | 106205 | -0.15 | 0.13 | 0.59 |  |
|  |  |  | rs3850634 | 118966 | -0.15 | 0.13 | 0.59 |  |
|  |  |  | rs1748199 | 125504 | -0.13 | 0.20 | 0.59 |  |
|  |  |  | rs4587594 | 202298 | -0.14 | 0.16 | 0.59 |  |
|  |  |  | rs11208004 | 213807 | -0.14 | 0.17 | 0.59 |  |
|  |  |  | rs10158897 | 18713 | -0.15 | 0.14 | 0.59 |  |
|  |  |  | rs1979722 | 8465 | -0.15 | 0.15 | 0.59 |  |
|  |  |  | rs10789119 | 166603 | -0.15 | 0.14 | 0.59 |  |
|  |  |  | rs1570694 | 171585 | -0.15 | 0.15 | 0.59 |  |
|  |  |  | rs995000 | 175894 | -0.15 | 0.14 | 0.59 |  |
|  |  |  | rs10889353 | 186564 | -0.15 | 0.15 | 0.59 |  |
|  |  |  | rs10159255 | 89184 | -0.15 | 0.13 | 0.59 |  |
|  |  |  | rs11207995 | 117919 | -0.15 | 0.14 | 0.59 |  |
|  |  |  | rs1168114 | 224411 | -0.12 | 0.24 | 0.59 |  |
|  |  |  | rs1168113 | 224466 | -0.07 | 0.48 | 0.70 |  |
|  |  |  | rs6682423 | 239431 | -0.09 | 0.37 | 0.61 |  |
|  |  |  | rs4409689 | 245733 | -0.09 | 0.38 | 0.62 |  |
|  |  |  | rs12136083 | 231441 | -0.16 | 0.13 | 0.59 |  |
|  |  |  | rs7518497 | 246064 | -0.15 | 0.14 | 0.59 |  |
|  |  |  | rs1168041 | 28618 | -0.19 | 0.07 | 0.59 |  |
|  |  |  | rs880694 | 241652 | -0.09 | 0.37 | 0.61 |  |
|  |  |  | rs9787151 | 247506 | -0.09 | 0.38 | 0.62 |  |
| CELSR2/PSRC1/SPRT1 | 1p13.3 | rs646776 |  |  | -0.05 | 0.78 | 0.89 |  |
|  |  |  | rs599839 | 3636 | 0.02 | 0.90 | 0.95 |  |
| APOB | 2p24.1 | rs6754295 |  |  | 0.32 | 5.55*10^-4*^ | 0.07 |  |
|  |  |  | rs676210 | 25341 | 0.35 | 1.68*10^-4*^ | 0.03^*^ |  |
|  |  |  | rs673548 | 31361 | 0.35 | 1.69*10^-4*^ | 0.03^*^ |  |
| LPL | 8p21.3 | rs328 |  |  | 0.10 | 0.44 | 0.68 |  |
| ABO | 9q34.2 | rs507666 |  |  | 0.21 | 0.04 | 0.59 |  |
| APOA1-A5 | 11q23.3 | rs3135506 |  |  | 9.53 | 0.68 | 0.82 |  |
| CETP | 16q13 | rs1800775 |  |  | -0.24 | 0.01 | 0.26 |  |
| LDLR | 19p13.2 | rs6511720 |  |  | -1.37 | 0.11 | 0.59 |  |
| APOC1-APOE | 19q13.32 | rs439401 |  |  | 0.02 | 0.87 | 0.93 |  |
| TIMD4\|HAVCR1 | 5q33.3 | rs1363232 |  |  | -0.17 | 0.14 | 0.59 |  |
|  |  |  | rs6874202 | 8206 | -0.17 | 0.06 | 0.59 |  |
|  |  |  | rs12657266 | 12581 | -0.17 | 0.06 | 0.59 |  |
|  |  |  | rs1501908 | 14747 | -0.17 | 0.06 | 0.59 |  |
| LCAT | 16q22.1 | rs255052 |  |  | 0.08 | 0.55 | 0.73 |  |
|  |  |  | rs255048 | 12146 | 0.10 | 0.51 | 0.73 |  |
| MAFB | 20q12 | rs6102085 |  |  | 0.16 | -0.05 | 0.59 |  |
|  |  |  | rs6016404 | 1091 | 0.09 | 0.31 | 0.59 |  |
| VLDL Total | |  |  |  |  |  |  |  |
| PCSK9 | 1p32.3 | rs11591147 |  |  | 17.46 | 0.006^*^ | 0.26 |  |
| ANGPTL3 | 1p31.3 | rs10889353 |  |  | -0.21 | 0.10 | 0.59 |  |
|  |  |  | rs1168089 | 4477 | -0.12 | 0.30 | 0.59 |  |
|  |  |  | rs7539035 | 11429 | -0.12 | 0.29 | 0.59 |  |
|  |  |  | rs6587980 | 27338 | -0.12 | 0.31 | 0.59 |  |
|  |  |  | rs1748195 | 68603 | -0.12 | 0.31 | 0.59 |  |
|  |  |  | rs4329540 | 91172 | -0.12 | 0.31 | 0.59 |  |
|  |  |  | rs1168018 | 117076 | -0.12 | 0.31 | 0.59 |  |
|  |  |  | rs1781195 | 129224 | -0.12 | 0.31 | 0.59 |  |
|  |  |  | rs656297 | 210601 | -0.13 | 0.28 | 0.59 |  |
|  |  |  | rs1168099 | 4663 | -0.14 | 0.24 | 0.59 |  |
|  |  |  | rs10889347 | 42370 | -0.12 | 0.31 | 0.59 |  |
|  |  |  | rs6678483 | 43754 | -0.12 | 0.31 | 0.59 |  |
|  |  |  | rs6675401 | 44221 | -0.12 | 0.31 | 0.59 |  |
|  |  |  | rs10789117 | 45931 | -0.12 | 0.31 | 0.59 |  |
|  |  |  | rs1168013 | 121358 | -0.12 | 0.31 | 0.59 |  |
|  |  |  | rs6690733 | 57885 | -0.12 | 0.31 | 0.59 |  |
|  |  |  | rs1748201 | 72690 | -0.12 | 0.32 | 0.59 |  |
|  |  |  | rs10889337 | 137589 | -0.12 | 0.31 | 0.59 |  |
|  |  |  | rs1168032 | 150449 | -0.12 | 0.31 | 0.59 |  |
|  |  |  | rs631106 | 216389 | -0.13 | 0.27 | 0.59 |  |
|  |  |  | rs637723 | 211468 | -0.11 | 0.33 | 0.60 |  |
|  |  |  | rs624660 | 197808 | -0.12 | 0.30 | 0.59 |  |
|  |  |  | rs583609 | 201400 | -0.12 | 0.30 | 0.59 |  |
|  |  |  | rs11207969 | 206445 | -0.12 | 0.30 | 0.59 |  |
|  |  |  | rs1167998 | 186564 | -0.12 | 0.31 | 0.59 |  |
|  |  |  | rs10889332 | 167338 | -0.11 | 0.35 | 0.60 |  |
|  |  |  | rs11207997 | 56290 | -0.12 | 0.31 | 0.59 |  |
|  |  |  | rs1168040 | 157621 | -0.12 | 0.31 | 0.59 |  |
|  |  |  | rs11208000 | 9822 | -0.12 | 0.31 | 0.59 |  |
| APOB | 2p24.1 | rs673548 |  |  | 0.27 | 0.02^*^ | 0.49 |  |
|  |  |  | rs6754295 | 31361 | 0.18 | 0.12 | 0.59 |  |
|  |  |  | rs6544366 | 33527 | 0.17 | 0.15 | 0.59 |  |
|  |  |  | rs11902417 | 38652 | 0.20 | 0.09 | 0.59 |  |
|  |  |  | rs6728178 | 43615 | 0.20 | 0.10 | 0.59 |  |
| GCKR | 2p23.3 | rs780094 |  |  | 0.09 | 0.42 | 0.66 |  |
| LPL | 8p21.3 | rs328 |  |  | 0.02 | 0.90 | 0.95 |  |
| APOA1-A5 | 11q23.3 | rs3135506 |  |  | -0.48 | 0.99 | 1.00 |  |
| CETP | 16q13 | rs1800775 |  |  | -0.05 | 0.67 | 0.81 |  |
| APOC1-APOE | 19q13.32 | rs439401 |  |  | 0.08 | 0.52 | 0.73 |  |
| TIMD4\|HAVCR1 | 5q33.3 | rs1354163 |  |  | 0.33 | 0.65 | 0.81 |  |
| LCAT | 16q22.1 | rs255052 |  |  | 0.04 | 0.84 | 0.91 |  |
|  |  |  | rs255048 | 12146 | 0.03 | 0.89 | 0.95 |  |
| MAFB | 20q12 | rs2865892 |  |  | 0.09 | 0.46 | 0.69 |  |
| JMJD1C | 10q21.3 | rs7923609 |  |  | 0.00 | 0.97 | 0.99 |  |
|  |  |  | rs7897379 | 167903 | -0.08 | 0.45 | 0.68 |  |
|  |  |  | rs10822184 | 203331 | -0.09 | 0.40 | 0.64 |  |
|  |  |  | rs10822186 | 216561 | -0.09 | 0.41 | 0.64 |  |
|  |  |  | rs4746203 | 190175 | 0.08 | 0.48 | 0.70 |  |
|  |  |  | rs3847326 | 198037 | 0.07 | 0.54 | 0.73 |  |
|  |  |  | rs10761785 | 184944 | NA | NA | NA |  |
| IDL total | |  |  |  |  |  |  |  |
| GCKR | 2p23.3 | rs780094 |  |  | 0.21 | 0.34 | 0.60 |  |
| LIPC | 15q22.1 | rs1532085 |  |  | 0.35 | 0.12 | 0.59 |  |
|  |  |  | rs7350789 | 3698 | 0.36 | 0.12 | 0.59 |  |
| CETP | 16q13 | rs1800775 |  |  | 0.36 | 0.12 | 0.59 |  |
| *TIMD4\|HAVCR1* | 5q33.3 | rs1363232 |  |  | -0.72 | 0.02^*^ | 0.43 |  |
|  |  |  | rs6874202 | 8206 | -0.38 | 0.11 | 0.59 |  |
|  |  |  | rs12657266 | 12581 | -0.38 | 0.11 | 0.59 |  |
|  |  |  | rs1501908 | 14747 | -0.37 | 0.12 | 0.59 |  |
| *LCAT* | 16q22.1 | rs255049 |  |  | 0.80 | 0.02^*^ | 0.56 |  |
| LDL Large | |  |  |  |  |  |  |  |
| PCSK9 | 1p32.3 | rs11591147 |  |  | -1700.27 | 0.02^*^ | 0.43 |  |
| APOB | 2p24.1 | rs1713222 |  |  | 81.43 | 0.52 | 0.73 |  |
|  |  |  | rs12714264 | 5805 | 94.21 | 0.32 | 0.59 |  |
| HMGCR | 5q13.3 | rs5744680 |  |  | -15.50 | 0.19 | 0.59 |  |
| LPL | 8p21.3 | rs2083637 |  |  | 2.86 | 0.85 | 0.91 |  |
|  |  |  | rs2165558 | 8915 | 3.79 | 0.80 | 0.89 |  |
| FADS1-3 | 11q12.2 | rs1535 |  |  | 9.33 | 0.45 | 0.68 |  |
| LIPC | 15q22.1 | rs1800588 |  |  | 23.09 | 0.08 | 0.59 |  |
| CETP | 16q13 | rs1864163 |  |  | -52.76 | 0.005^*^ | 0.26 |  |
| LIPG | 18q21.1 | rs8090363 |  |  | 3.23 | 0.81 | 0.89 |  |
| LDLR | 19p13.2 | rs6511720 |  |  | -222.30 | 0.07 | 0.59 |  |
| APOC1-APOE | 19q13.32 | rs4803750 |  |  | 23.70 | 0.30 | 0.59 |  |
|  |  |  | rs1531517 | 5454 | -1.25 | 0.95 | 0.99 |  |
| PLTP | 20q13.12.B | rs4810479 |  |  | -12.85 | 0.30 | 0.59 |  |
| TIMD4\|HAVCR1 | 5q33.3 | rs7706174 |  |  | 3.34 | 0.82 | 0.90 |  |
| MAFB | 20q12 | rs6102085 |  |  | 3.19 | 0.79 | 0.89 |  |
|  |  |  | rs6016404 | 1091 | -10.13 | 0.41 | 0.64 |  |
| LDL Small | |  |  |  |  |  |  |  |
| CELSR2/PSRC1/SPRT1 | 1p13.3 | rs646776 |  |  | -49.84 | 0.17 | 0.59 |  |
|  |  |  | rs599839 | 3636 | -47.39 | 0.14 | 0.59 |  |
| GCKR | 2p23.3 | rs780094 |  |  | 12.92 | 0.48 | 0.70 |  |
| LPL | 8p21.3 | rs2083637 |  |  | -5.89 | 0.79 | 0.89 |  |
|  |  |  | rs2165558 | 8915 | -12.56 | 0.58 | 0.75 |  |
| TRIB1 | 8q24.13 | rs6982636 |  |  | 9.87 | 0.60 | 0.76 |  |
|  |  |  | rs2980856 | 2936 | -49.02 | 0.07 | 0.59 |  |
|  |  |  | rs10808546 | 16503 | -37.37 | 0.21 | 0.59 |  |
| APOA1-A5 | 11q23.3 | rs3135506 |  |  | -3212.93 | 0.52 | 0.73 |  |
| LIPC | 15q22.1 | rs1800588 |  |  | -0.72 | 0.97 | 0.99 |  |
| CETP | 16q13 | rs1800775 |  |  | 3.30 | 0.86 | 0.93 |  |
| APOC1-APOE | 19q13.32 | rs769449 |  |  | 69.81 | 0.06 | 0.59 |  |
| PLTP | 20q13.12.B | rs6065906 |  |  | 44.71 | 0.44 | 0.68 |  |
| TIMD4\|HAVCR1 | 5q33.3 | rs7706174 |  |  | -16.62 | 0.46 | 0.69 |  |
| LCAT | 16q22.1 | rs7200210 |  |  | -38.86 | 0.70 | 0.83 |  |
|  |  |  | rs7185536 | 10268 | -39.81 | 0.69 | 0.83 |  |
|  |  |  | rs14178 | 12365 | -25.48 | 0.81 | 0.89 |  |
|  |  |  | rs16957590 | 47171 | 250.48 | 0.11 | 0.59 |  |
|  |  |  | rs8061384 | 70022 | 329.72 | 0.09 | 0.59 |  |
|  |  |  | rs8051653 | 133381 | 373.01 | 0.09 | 0.59 |  |
|  |  |  | rs8058835 | 167031 | 219.89 | 0.13 | 0.59 |  |
|  |  |  | rs16957524 | 183891 | 220.10 | 0.10 | 0.59 |  |
|  |  |  | rs6499141 | 184762 | 260.07 | 0.20 | 0.59 |  |
|  |  |  | rs7200950 | 292144 | 302.38 | 0.37 | 0.61 |  |
|  |  |  | rs7195415 | 292839 | 310.43 | 0.35 | 0.60 |  |
|  |  |  | rs9972635 | 299316 | 184.51 | 0.56 | 0.73 |  |
| MAFB | 20q12 | rs2865892 |  |  | 55.21 | 0.007^*^ | 0.27 |  |
| LDL Total | |  |  |  |  |  |  |  |
| PCSK9 | 1p32.3 | rs11591147 |  |  | -31.53 | 0.02^*^ | 0.51 |  |
| CELSR2/PSRC1/SPRT1 | 1p13.3 | rs646776 |  |  | -0.58 | 0.21 | 0.59 |  |
|  |  |  | rs599839 | 3636 | -0.46 | 0.26 | 0.59 |  |
| APOB | 2p24.1 | rs312985 |  |  | -0.11 | 0.94 | 0.98 |  |
|  |  |  | rs527034 | 3981 | -0.06 | 0.97 | 0.99 |  |
|  |  |  | rs4560142 | 4912 | -0.52 | 0.67 | 0.81 |  |
|  |  |  | rs4591370 | 4937 | -0.06 | 0.97 | 0.99 |  |
|  |  |  | rs1712247 | 5146 | -0.06 | 0.97 | 0.99 |  |
|  |  |  | rs312970 | 8990 | -0.06 | 0.97 | 0.99 |  |
|  |  |  | rs576203 | 14818 | -0.06 | 0.97 | 0.99 |  |
|  |  |  | rs506585 | 18377 | -0.06 | 0.97 | 0.99 |  |
|  |  |  | rs478442 | 20411 | -0.06 | 0.97 | 0.99 |  |
|  |  |  | rs1878512 | 41553 | 1.37 | 0.52 | 0.73 |  |
|  |  |  | rs10169543 | 45184 | 1.41 | 0.51 | 0.73 |  |
|  |  |  | rs365946 | 60524 | 0.92 | 0.56 | 0.73 |  |
|  |  |  | rs541041 | 83830 | -0.16 | 0.91 | 0.96 |  |
|  |  |  | rs562338 | 90484 | -0.06 | 0.97 | 0.99 |  |
| GCKR | 2p23.3 | rs780094 |  |  | 0.17 | 0.46 | 0.69 |  |
| TRIB1 | 8q24.13 | rs6982636 |  |  | 0.09 | 0.70 | 0.83 |  |
|  |  |  | rs2980856 | 2936 | -0.59 | 0.09 | 0.59 |  |
|  |  |  | rs10808546 | 16503 | -0.37 | 0.32 | 0.59 |  |
| APOA1-A5 | 11q23.3 | rs3135506 |  |  | -41.69 | 0.51 | 0.73 |  |
| CETP | 16q13 | rs1800775 |  |  | -0.08 | 0.75 | 0.86 |  |
| LDLR | 19p13.2 | rs6511720 |  |  | -1.42 | 0.54 | 0.73 |  |
| APOC1-APOE | 19q13.32 | rs769449 |  |  | 0.87 | 0.06 | 0.59 |  |
| TIMD4\|HAVCR1 | 5q33.3 | rs1363232 |  |  | -0.22 | 0.48 | 0.70 |  |
|  |  |  | rs6874202 | 8206 | -0.25 | 0.33 | 0.60 |  |
|  |  |  | rs12657266 | 12581 | -0.25 | 0.32 | 0.59 |  |
|  |  |  | rs1501908 | 14747 | -0.25 | 0.31 | 0.59 |  |
| MAFB | 20q12 | rs2865892 |  |  | 0.76 | 0.003^*^ | 0.26 |  |
| HDL Large | |  |  |  |  |  |  |  |
| LPL | 8p21.3 | rs331 |  |  | -0.02 | 0.52 | 0.73 |  |
|  |  |  | rs2165558 | 35855 | -0.01 | 0.71 | 0.83 |  |
| FADS1-3 | 11q12.2 | rs174546 |  |  | 0.06 | 0.03^*^ | 0.59 |  |
| CCDC92/DNAH10/ZNF664 | 12q24.31.B | rs7307277 |  |  | -0.01 | 0.79 | 0.89 |  |
|  |  |  | rs11057396 | 56094 | -0.04 | 0.38 | 0.62 |  |
|  |  |  | rs12298484 | 56482 | -0.04 | 0.35 | 0.60 |  |
|  |  |  | rs10846580 | 59703 | -0.04 | 0.35 | 0.60 |  |
|  |  |  | rs9971695 | 61665 | -0.04 | 0.35 | 0.60 |  |
|  |  |  | rs3802999 | 61058 | -0.04 | 0.35 | 0.60 |  |
|  |  |  | rs11057397 | 55428 | -0.04 | 0.37 | 0.61 |  |
|  |  |  | rs4930721 |  | -0.03 | 0.47 | 0.70 |  |
| LIPC | 15q22.1 | rs1800588 |  |  | 0.08 | 0.01^*^ | 0.43 |  |
| CETP | 16q13 | rs1800775 |  |  | -0.01 | 0.61 | 0.77 |  |
| LIPG | 18q21.1 | rs4939883 |  |  | -0.02 | 0.60 | 0.76 |  |
|  |  |  | rs10438978 | 9028 | -0.03 | 0.30 | 0.59 |  |
|  |  |  | rs7239867 | 2497 | -0.02 | 0.66 | 0.81 |  |
|  |  |  | rs7240405 | 8124 | -0.02 | 0.66 | 0.81 |  |
| PLTP | 20q13.12.B | rs6065904 |  |  | -0.03 | 0.34 | 0.60 |  |
| TIMD4\|HAVCR1 | 5q33.3 | rs6873053 |  |  | -0.30 | 0.53 | 0.73 |  |
| LCAT | 16q22.1 | rs2271293 |  |  | -0.06 | 0.56 | 0.73 |  |
|  |  |  | rs2292318 | 83636 | 0.03 | 0.50 | 0.72 |  |
| MAFB | 20q12 | rs2865879 |  |  | 0.01 | 0.63 | 0.78 |  |
|  |  |  | rs761564 | 9618 | -0.05 | 0.35 | 0.60 |  |
|  |  |  | rs2050204 | 11427 | -0.06 | 0.29 | 0.59 |  |
| HDL Medium | |  |  |  |  |  |  |  |
| APOA2 | 1q23.3 | rs4073054 |  |  | 0.16 | 0.03^*^ | 0.59 |  |
| FADS1-3 | 11q12.2 | rs174537 |  |  | -0.03 | 0.53 | 0.73 |  |
| APOC1-APOE | 19q13.32 | rs405509 |  |  | 0.07 | 0.24 | 0.59 |  |
| *TIMD4\|HAVCR1* | 5q33.3 | rs4704810 |  |  | -0.01 | 0.87 | 0.93 |  |
| HDL Small | |  |  |  |  |  |  |  |
| GCKR | 2p23.3 | rs780094 |  |  | 0.73 | 0.009^*^ | 0.30 |  |
| APOA1-A5 | 11q23.3 | rs518181 |  |  | -0.60 | 0.07 | 0.59 |  |
|  |  |  | rs7120706 | 57300 | -0.32 | 0.33 | 0.60 |  |
|  |  |  | rs7120963 | 57474 | -0.32 | 0.33 | 0.60 |  |
|  |  |  | rs10892052 | 61526 | -0.23 | 0.51 | 0.73 |  |
|  |  |  | rs10892053 | 84216 | -0.31 | 0.35 | 0.60 |  |
|  |  |  | rs10892063 | 123368 | -0.29 | 0.38 | 0.62 |  |
|  |  |  | rs11216267 | 179605 | -0.30 | 0.37 | 0.61 |  |
|  |  |  | rs10892072 | 187526 | -0.33 | 0.33 | 0.59 |  |
|  |  |  | rs7120515 | 219139 | -0.63 | 0.04 | 0.59 |  |
| LIPC | 15q22.1 | rs1800588 |  |  | 0.19 | 0.55 | 0.73 |  |
| PLTP | 20q13.12.B | rs4810479 |  |  | -0.51 | 0.07 | 0.59 |  |
| *LCAT* | 16q22.1 | rs1109166 |  |  | -0.56 | 0.24 | 0.59 |  |
|  |  |  | rs6499145 | 47781 | 0.24 | 0.81 | 0.89 |  |
|  |  |  | rs10468274 | 55040 | -0.25 | 0.76 | 0.87 |  |
|  |  |  | rs8060686 | 65865 | 0.71 | 0.39 | 0.64 |  |
|  |  |  | rs11553287 | 71975 | 0.25 | 0.80 | 0.89 |  |
|  |  |  | rs13334918 | 76039 | 0.26 | 0.79 | 0.89 |  |
|  |  |  | rs7198357 | 92763 | 0.27 | 0.78 | 0.89 |  |
|  |  |  | rs6499143 | 103118 | 0.31 | 0.75 | 0.87 |  |
|  |  |  | rs7199443 | 136253 | 0.70 | 0.50 | 0.72 |  |
|  |  |  | rs9928531 | 144211 | 0.77 | 0.46 | 0.69 |  |
|  |  |  | rs28563117 | 195252 | 1.07 | 0.33 | 0.59 |  |
|  |  |  | rs12449157 | 268485 | 1.12 | 0.32 | 0.59 |  |
| HDL total | |  |  |  |  |  |  |  |
| GCKR | 2p23.3 | rs1260326 |  |  | 0.45 | 0.11 | 0.59 |  |
| APOA1-A5 | 11q23.3 | rs518181 |  |  | -0.34 | 0.31 | 0.59 |  |
|  |  |  | rs7120706 | 57300 | -0.43 | 0.20 | 0.59 |  |
|  |  |  | rs7120963 | 57474 | -0.43 | 0.20 | 0.59 |  |
|  |  |  | rs10892052 | 61526 | -0.50 | 0.15 | 0.59 |  |
|  |  |  | rs10892053 | 84216 | -0.41 | 0.21 | 0.59 |  |
|  |  |  | rs10892063 | 123368 | -0.36 | 0.28 | 0.59 |  |
|  |  |  | rs11216267 | 179605 | -0.35 | 0.29 | 0.59 |  |
|  |  |  | rs10892072 | 187526 | -0.35 | 0.29 | 0.59 |  |
|  |  |  | rs7120515 | 219139 | 0.01 | 0.97 | 0.99 |  |
| CETP | 16q13 | rs7499892 |  |  | -0.56 | 0.16 | 0.59 |  |
|  |  |  | rs7203984 | 7332 | -0.21 | 0.53 | 0.73 |  |
| PLTP | 20q13.12.B | rs6065906 |  |  | -0.40 | 0.64 | 0.80 |  |
| *LCAT* | 16q22.1 | rs1109166 |  |  | 0.00 | 1.00 | 1.00 |  |
|  |  |  | rs6499145 | 47781 | 0.44 | 0.66 | 0.81 |  |
|  |  |  | rs10468274 | 55040 | -0.27 | 0.74 | 0.86 |  |
|  |  |  | rs8060686 | 65865 | 0.95 | 0.25 | 0.59 |  |
|  |  |  | rs11553287 | 71975 | 0.40 | 0.68 | 0.82 |  |
|  |  |  | rs13334918 | 76039 | 0.42 | 0.67 | 0.81 |  |
|  |  |  | rs7198357 | 92763 | 0.35 | 0.71 | 0.83 |  |
|  |  |  | rs6499143 | 103118 | 0.55 | 0.57 | 0.74 |  |
|  |  |  | rs7199443 | 136253 | 0.84 | 0.41 | 0.64 |  |
|  |  |  | rs9928531 | 144211 | 0.84 | 0.42 | 0.66 |  |
|  |  |  | rs28563117 | 195252 | 1.15 | 0.29 | 0.59 |  |
|  |  |  | rs12449157 | 268485 | 1.13 | 0.31 | 0.59 |  |
| *MAFB* | 20q12 | rs6016408 |  |  | -0.57 | 0.28 | 0.59 |  |
|  |  |  | rs6016400 | 11005 | -0.29 | 0.29 | 0.59 |  |
|  |  |  | rs735031 | 13417 | -0.08 | 0.76 | 0.87 |  |
|  |  |  | rs6029251 | 14697 | 0.00 | 1.00 | 1.00 |  |
| SBF2 | 11p15.4 | rs7938647 |  |  | -3.99 | 0.22 | 0.59 |  |
|  |  |  | rs7950447 | 83402 | -4.00 | 0.19 | 0.59 |  |
|  |  |  | rs7938491 | 109969 | -0.31 | 0.64 | 0.80 |  |
|  |  |  | rs7935934 | 139762 | -1.24 | 0.13 | 0.59 |  |
|  |  |  | rs1822292 | 183255 | -1.26 | 0.14 | 0.59 |  |
|  |  |  | rs7114303 | 34264 | -0.22 | 0.53 | 0.73 |  |
|  |  |  | rs1372807 | 76876 | -0.52 | 0.13 | 0.59 |  |
|  |  |  | rs4909917 | 80258 | -0.29 | 0.40 | 0.64 |  |
|  |  |  | rs4576815 | 74195 | -0.27 | 0.44 | 0.68 |  |
|  |  |  | rs4910086 | 75443 | -0.27 | 0.44 | 0.68 |  |
| VLDL diameter | |  |  |  |  |  |  |  |
| APOB | 2p24.1 | rs676210 |  |  | -0.06 | 0.05 | 0.59 |  |
|  |  |  | rs6754295 | 25341 | -0.05 | 0.06 | 0.59 |  |
|  |  |  | rs6544366 | 27507 | -0.04 | 0.14 | 0.59 |  |
|  |  |  | rs11902417 | 32632 | -0.05 | 0.07 | 0.59 |  |
|  |  |  | rs6728178 | 37595 | -0.06 | 0.06 | 0.59 |  |
| GCKR | 2p23.3 | rs1260326 |  |  | 0.08 | 0.005^*^ | 0.26 |  |
| intergenic, PPP1R3B | 8p23.1 | rs983309 |  |  | 0.18 | 0.15 | 0.59 |  |
| LDL diameter | |  |  |  |  |  |  |  |
| GCKR | 2p23.3 | rs780094 |  |  | -0.008 | 0.76 | 0.87 |  |
| LPL | 8p21.3 | rs2083637 |  |  | 0.01 | 0.74 | 0.86 |  |
|  |  |  | rs2165558 | 8915 | 0.02 | 0.58 | 0.75 |  |
| APOA1-A5 | 11q23.3 | rs3135506 |  |  | 4.13 | 0.55 | 0.73 |  |
| LIPC | 15q22.1 | rs1800588 |  |  | 0.03 | 0.30 | 0.59 |  |
| CETP | 16q13 | rs1800775 |  |  | -0.02 | 0.44 | 0.68 |  |
| LIPG | 18q21.1 | rs4939883 |  |  | -0.04 | 0.20 | 0.59 |  |
|  |  |  | rs10438978 | 9028 | -0.03 | 0.30 | 0.59 |  |
|  |  |  | rs7239867 | 2497 | -0.06 | 0.11 | 0.59 |  |
|  |  |  | rs7240405 | 8124 | -0.06 | 0.10 | 0.59 |  |
| PLTP | 20q13.12.B | rs6065906 |  |  | -0.07 | 0.36 | 0.61 |  |
| TIMD4\|HAVCR1 | 5q33.3 | rs7706174 |  |  | 0.02 | 0.55 | 0.73 |  |
| LCAT | 16q22.1 | rs7200210 |  |  | 0.19 | 0.16 | 0.59 |  |
|  |  |  | rs7185536 | 10268 | 0.19 | 0.16 | 0.59 |  |
|  |  |  | rs14178 | 12365 | 0.18 | 0.21 | 0.59 |  |
|  |  |  | rs16957590 | 47171 | -0.12 | 0.58 | 0.74 |  |
|  |  |  | rs8061384 | 70022 | -0.22 | 0.40 | 0.64 |  |
|  |  |  | rs8051653 | 133381 | -0.36 | 0.23 | 0.59 |  |
|  |  |  | rs8058835 | 167031 | -0.14 | 0.46 | 0.69 |  |
|  |  |  | rs16957524 | 183891 | -0.19 | 0.28 | 0.59 |  |
|  |  |  | rs6499141 | 184762 | -0.28 | 0.31 | 0.59 |  |
|  |  |  | rs7200950 | 292144 | -0.38 | 0.41 | 0.64 |  |
|  |  |  | rs7195415 | 292839 | -0.37 | 0.40 | 0.64 |  |
|  |  |  | rs9972635 | 299316 | -0.22 | 0.60 | 0.76 |  |
| MAFB | 20q12 | rs2865892 |  |  | -0.06 | 0.04^*^ | 0.59 |  |
| HDL diameter | |  |  |  |  |  |  |  |
| GCKR | 2p23.3 | rs780094 |  |  | -1.54*10^-4^ | 0.99 | 1.00 |  |
| LPL | 8p21.3 | rs331 |  |  | -0.03 | 0.28 | 0.59 |  |
|  |  |  | rs2165558 | 35855 | -0.01 | 0.64 | 0.80 |  |
| FADS1-3 | 11q12.2 | rs1535 |  |  | 0.06 | 0.002^*^ | 0.15 |  |
| LIPC | 15q22.1 | rs1800588 |  |  | 0.03 | 0.18 | 0.59 |  |
| CETP | 16q13 | rs1800775 |  |  | 0.01 | 0.57 | 0.73 |  |
| LIPG | 18q21.1 | rs4939883 |  |  | 0.05 | 0.03^*^ | 0.59 |  |
|  |  |  | rs10438978 | 9028 | 0.03 | 0.23 | 0.59 |  |
|  |  |  | rs7239867 | 2497 | 0.03 | 0.30 | 0.59 |  |
|  |  |  | rs7240405 | 8124 | 0.03 | 0.30 | 0.59 |  |
| PLTP | 20q13.12.B | rs6065906 |  |  | -0.12 | 0.06 | 0.59 |  |
| LCAT | 16q22.1 | rs4986970 |  |  | 1.91*10^-4^ | 1.00 | 1.00 |  |
| MAFB | 20q12 | rs2865892 |  |  | -0.01 | 0.53 | 0.73 |  |

^a^ models are adjusted for age, gender, BMI, current smoking status, study centers, and Principle Components (PC1-PC4)

^*^ significant p-value or Q-value results with asterisk

**S 7 Table** Selection of proxy SNPs.

| Original SNP | Proxy SNPs examined | | |
| --- | --- | --- | --- |
|  | African Americans | Hispanic Americans | Chinese Americans |
| rs1260326 |  |  |  |
| rs1059611 |  |  |  |
|  | rs3735964 | rs12682115 | rs7016880 |
|  | rs12679834 | rs17411024 | rs7816447 |
|  | rs17482753 |  |  |
|  | rs12678919 |  |  |
|  | rs12682115 |  |  |
|  | rs10503669 |  |  |
|  | rs17411024 |  |  |
|  | rs7016880 |  |  |
|  | rs7816447 |  |  |
| rs3135506 |  |  |  |
| rs439401 |  |  |  |
| rs1354163 |  |  |  |
|  | rs1501910 | rs1948759 |  |
|  | rs6896499 | rs905211 |  |
|  | rs1948759 | rs1393209 |  |
|  | rs2902132 | rs1553316 |  |
|  | rs905211 | rs1553317 |  |
|  | rs1393209 |  |  |
|  | rs1553316 |  |  |
|  | rs7731951 |  |  |
|  | rs1553317 |  |  |
| rs1109166 |  |  |  |
|  | rs7199443 | rs8060686 | rs6499145 |
|  |  | rs9928531 | rs10468274 |
|  |  | rs12449157 | rs8060686 |
|  |  |  | rs11553287 |
|  |  |  | rs13334918 |
|  |  |  | rs7198357 |
|  |  |  | rs6499143 |
|  |  |  | rs7199443 |
|  |  |  | rs9928531 |
|  |  |  | rs28563117 |
|  |  |  | rs12449157 |
| rs1076759 |  |  |  |
|  | rs6102127 |  |  |
|  |  |  |  |
| rs10889353 |  |  |  |
|  | rs1168089 | rs1168040 | rs1168089 |
|  | rs7539035 | rs1168113 | rs7539035 |
|  | rs6587980 |  | rs6587980 |
|  | rs1748195 |  | rs1748195 |
|  | rs4329540 |  | rs4329540 |
|  | rs1168018 |  | rs1168018 |
|  | rs1781195 |  | rs1781195 |
|  | rs656297 |  | rs656297 |
|  | rs10889333 |  | rs1168099 |
|  | rs1168099 |  | rs10889347 |
|  | rs10889347 |  | rs6678483 |
|  | rs6678483 |  | rs6675401 |
|  | rs6675401 |  | rs10789117 |
|  | rs10789117 |  | rs1168013 |
|  | rs1168013 |  | rs6690733 |
|  | rs10158897 |  | rs1748201 |
|  | rs6690733 |  | rs10889337 |
|  | rs1748201 |  | rs1168032 |
|  | rs10889337 |  | rs631106 |
|  | rs1168032 |  | rs637723 |
|  | rs10889335 |  | rs624660 |
|  | rs1979722 |  | rs583609 |
|  | rs1748199 |  | rs11207969 |
|  | rs3913007 |  | rs1167998 |
|  | rs631106 |  | rs10889332 |
|  | rs637723 |  | rs11207997 |
|  | rs4350231 |  | rs1168040 |
|  | rs624660 |  | rs11208000 |
|  | rs583609 |  |  |
|  | rs11207969 |  |  |
|  | rs1167998 |  |  |
|  | rs10889332 |  |  |
|  | rs880694 |  |  |
|  | rs11207997 |  |  |
|  | rs1168040 |  |  |
|  | rs11208000 |  |  |
|  | rs12136083 |  |  |
|  | rs7518497 |  |  |
|  | rs1168114 |  |  |
|  | rs1168113 |  |  |
|  | rs6682423 |  |  |
|  | rs4409689 |  |  |
|  | rs9787151 |  |  |
| rs673548 |  |  |  |
|  | rs6754295 |  | rs6754295 |
|  |  |  | rs6544366 |
|  |  |  | rs11902417 |
|  |  |  | rs6728178 |
| rs1260326 |  |  |  |
| rs2240466 |  |  |  |
|  |  | rs17145750 |  |
| rs12768534 |  |  |  |
|  | rs10822184 | rs10822184 | rs10822184 |
|  | rs10822186 | rs10822186 | rs10822186 |
|  | rs10761785 |  |  |
| rs255052 |  |  |  |
|  | rs255048 | rs255048 | rs255048 |
| rs2865892 |  |  |  |
|  | rs2206665 |  |  |
| rs11591147 |  |  |  |
| rs1167998 |  |  |  |
|  | rs12116574 |  | rs4350231 |
|  | rs3850634 |  | rs3913007 |
|  | rs4587594 |  | rs10889333 |
|  | rs11208004 |  | rs10889335 |
|  | rs10789119 |  | rs12116574 |
|  | rs1570694 |  | rs3850634 |
|  | rs995000 |  | rs1748199 |
|  | rs10889353 |  | rs4587594 |
|  | rs10159255 |  | rs11208004 |
|  | rs11207995 |  | rs10158897 |
|  | rs1168041 |  | rs1979722 |
|  |  |  | rs10789119 |
|  |  |  | rs1570694 |
|  |  |  | rs995000 |
|  |  |  | rs10889353 |
|  |  |  | rs10159255 |
|  |  |  | rs11207995 |
|  |  |  | rs1168114 |
|  |  |  | rs1168113 |
|  |  |  | rs6682423 |
|  |  |  | rs4409689 |
|  |  |  | rs12136083 |
|  |  |  | rs7518497 |
|  |  |  | rs1168041 |
|  |  |  | rs880694 |
|  |  |  | rs9787151 |
| rs646776 |  |  |  |
|  | rs7528419 |  | rs599839 |
|  | rs599839 |  |  |
| rs6754295 |  |  |  |
|  | rs6544366 |  | rs676210 |
|  | rs11902417 |  | rs673548 |
|  | rs6728178 |  |  |
|  | rs676210 |  |  |
|  | rs673548 |  |  |
| rs328 |  |  |  |
| rs507666 |  |  |  |
| rs1800775 |  |  |  |
| rs6511720 |  |  |  |
|  | rs2228671 | rs2228671 |  |
| rs1363232 |  |  |  |
|  | rs7724832 | rs6874202 | rs6874202 |
|  | rs6874202 | rs12657266 | rs12657266 |
|  | rs12657266 | rs1501908 | rs1501908 |
|  | rs1501908 |  |  |
|  | rs7717984 |  |  |
| rs6102085 |  |  |  |
|  | rs6016404 |  | rs6016404 |
| rs780094 |  |  |  |
| rs7923609 |  |  |  |
|  | rs4746203 | rs4746203 | rs7897379 |
|  | rs3847326 | rs3847326 | rs4746203 |
|  |  | rs10761785 | rs3847326 |
|  |  |  | rs10761785 |
| rs1532085 |  |  |  |
|  | rs7350789 |  | rs7350789 |
| rs255049 |  |  |  |
| rs1713222 |  |  |  |
|  | rs12714264 |  | rs12714264 |
| rs5744680 |  |  |  |
|  | rs3846662 | rs3846662 |  |
|  | rs12654264 | rs12654264 |  |
|  | rs11749783 | rs11749783 |  |
|  | rs7703051 | rs7703051 |  |
|  | rs3846663 | rs3846663 |  |
|  | rs2335418 | rs2335418 |  |
|  | rs11957260 | rs11957260 |  |
| rs2083637 |  |  |  |
|  | rs765547 | rs327 | rs2165558 |
|  | rs1919484 | rs331 |  |
|  | rs17411126 | rs301 |  |
|  | rs4922117 | rs297 |  |
|  | rs17489282 | rs12541912 |  |
|  | rs17411045 | rs4523270 |  |
|  | rs17411031 | rs2165558 |  |
|  | rs17489268 | rs15285 |  |
|  | rs327 | rs11986942 |  |
|  | rs331 | rs13702 |  |
|  | rs301 | rs326 |  |
|  | rs297 | rs1441766 |  |
|  | rs3916027 |  |  |
|  | rs12541912 |  |  |
|  | rs4523270 |  |  |
|  | rs2165558 |  |  |
|  | rs15285 |  |  |
|  | rs11986942 |  |  |
|  | rs13702 |  |  |
|  | rs326 |  |  |
|  | rs1441766 |  |  |
| rs1535 |  |  |  |
|  | rs174568 |  |  |
|  | rs174546 |  |  |
|  | rs174576 |  |  |
|  | rs174566 |  |  |
|  | rs174577 |  |  |
|  | rs174537 |  |  |
|  | rs174583 |  |  |
|  | rs102275 |  |  |
|  | rs174535 |  |  |
|  | rs4246215 |  |  |
|  | rs174556 |  |  |
|  | rs174555 |  |  |
|  | rs174549 |  |  |
| rs1800588 |  |  |  |
| rs1864163 |  |  |  |
| rs8090363 |  |  |  |
| rs4803750 |  |  |  |
|  | rs1531517 | rs1531517 | rs1531517 |
| rs4810479 |  |  |  |
| rs7706174 |  |  |  |
|  | rs6878732 | rs6878732 |  |
|  | rs10059699 |  |  |
| rs6982636 |  |  |  |
|  | rs2980856 | rs10808546 | rs2980856 |
|  | rs17321515 |  | rs10808546 |
|  | rs2980869 |  |  |
|  | rs10808546 |  |  |
|  | rs6982502 |  |  |
|  | rs2001945 |  |  |
| rs769449 |  |  |  |
| rs6065906 |  |  |  |
|  | rs7679 |  |  |
|  | rs6073972 |  |  |
| rs7200210 |  |  |  |
|  | rs6499141 | rs8061384 | rs7185536 |
|  | rs7200950 | rs7200950 | rs14178 |
|  | rs7195415 | rs7195415 | rs16957590 |
|  | rs9972635 | rs9972635 | rs8061384 |
|  |  |  | rs8051653 |
|  |  |  | rs8058835 |
|  |  |  | rs16957524 |
|  |  |  | rs6499141 |
|  |  |  | rs7200950 |
|  |  |  | rs7195415 |
|  |  |  | rs9972635 |
| rs312985 |  |  |  |
|  |  |  | rs527034 |
|  |  |  | rs4560142 |
|  |  |  | rs4591370 |
|  |  |  | rs1712247 |
|  |  |  | rs312970 |
|  |  |  | rs576203 |
|  |  |  | rs506585 |
|  |  |  | rs478442 |
|  |  |  | rs1878512 |
|  |  |  | rs10169543 |
|  |  |  | rs365946 |
|  |  |  | rs541041 |
|  |  |  | rs562338 |
| rs331 |  |  |  |
|  | rs2083637 | rs2083637 |  |
| rs174546 |  |  |  |
|  | rs1535 |  |  |
| rs7307277 |  |  |  |
|  | rs7311969 | rs10846580 | rs11057396 |
|  | rs1187415 |  | rs12298484 |
|  | rs12823740 |  | rs10846580 |
|  | rs7973683 |  | rs9971695 |
|  | rs4765219 |  | rs3802999 |
|  | rs7312404 |  | rs11057397 |
|  | rs4930726 |  | rs4930721 |
|  | rs11057396 |  |  |
|  | rs12298484 |  |  |
|  | rs10846580 |  |  |
|  | rs9971695 |  |  |
|  | rs3802999 |  |  |
|  | rs11057397 |  |  |
|  | rs7305864 |  |  |
|  | rs6488914 |  |  |
|  | rs2178663 |  |  |
|  | rs11057401 |  |  |
|  | rs4930721 |  |  |
|  | rs1054852 |  |  |
| rs4939883 |  |  |  |
|  | rs10438978 | rs10438978 | rs10438978 |
|  | rs7239867 | rs7239867 | rs7239867 |
|  | rs7240405 | rs7240405 | rs7240405 |
|  | rs2156552 | rs2156552 |  |
|  | rs1943973 | rs1943973 |  |
| rs6065904 |  |  |  |
| rs6873053 |  |  |  |
| rs2271293 |  |  |  |
|  | rs1124324 | rs4474673 | rs2292318 |
|  | rs3809630 |  |  |
|  | rs16942887 |  |  |
|  | rs4474673 |  |  |
|  | rs2292318 |  |  |
| rs2865879 |  |  |  |
|  | rs2903114 | rs761564 | rs761564 |
|  | rs761564 | rs2050204 | rs2050204 |
|  | rs2050204 |  |  |
|  | rs2425444 |  |  |
|  | rs2425425 |  |  |
|  | rs2425451 |  |  |
|  | rs3091957 |  |  |
|  | rs2425421 |  |  |
| rs4073054 |  |  |  |
| rs174537 |  |  |  |
|  | rs108499 | rs102275 |  |
|  |  | rs174566 |  |
|  |  | rs174576 |  |
|  |  | rs174577 |  |
|  |  | rs174583 |  |
| rs405509 |  |  |  |
| rs4704810 |  |  |  |
| rs518181 |  |  |  |
|  | rs7120706 |  | rs7120706 |
|  | rs7120963 |  | rs7120963 |
|  | rs10892052 |  | rs10892052 |
|  | rs10892053 |  | rs10892053 |
|  | rs10892063 |  | rs10892063 |
|  | rs11216267 |  | rs11216267 |
|  | rs10892072 |  | rs10892072 |
|  | rs7120515 |  | rs7120515 |
| rs7499892 |  |  |  |
|  |  |  | rs7203984 |
| rs6016408 |  |  |  |
|  | rs6016400 |  | rs6016400 |
|  | rs735031 |  | rs735031 |
|  | rs6029251 |  | rs6029251 |
| rs7938647 |  |  |  |
|  | rs11042595 | rs7935934 | rs7950447 |
|  | rs11042623 | rs1822292 | rs7938491 |
|  | rs7938491 | rs4910081 | rs7935934 |
|  | rs7935934 | rs7114303 | rs1822292 |
|  | rs1822292 | rs1372807 | rs7114303 |
|  | rs10840327 | rs4909917 | rs1372807 |
|  | rs4910081 | rs4576815 | rs4909917 |
|  | rs7114303 | rs4910086 | rs4576815 |
|  | rs1372807 | rs7104093 | rs4910086 |
|  | rs4909917 | rs11042543 |  |
|  | rs4576815 |  |  |
|  | rs4910086 |  |  |
|  | rs7104093 |  |  |
|  | rs11042543 |  |  |
| rs676210 |  |  |  |
| rs983309 |  |  |  |
| rs4986970 |  |  |  |

**S 8 Table** Secondary Analyses - associations of GRSs and lipoprotein phenotypes among individuals not reporting use of lipid medication nor presence of type II diabetes

| Lipoprotein subfraction | European-Americans (n=1890) | | | African-Americans (n=1204) | | | Hispanic-Americans (n=1103) | | | Chinese-Americans (n=617) | | |
| --- | --- | --- | --- | --- | --- | --- | --- | --- | --- | --- | --- | --- |
|  | **β** | ***P*** | **Q** | **β** | ***P*** | **Q** | **β** | ***P*** | **Q** | **β** | ***P*** | **Q** |
| VLDL Large | 0.09 | 4.77*10^-05^ | **7.95*10^-05^** | 0.08 | 4.54*10^-03^ | **0.01** | 0.10 | 4.43*10^-05^ | **6.05*10^-05^** | 0.01 | 0.80 | 0.91 |
| VLDL Medium | 0.10 | 5.88*10^-05^ | **8.82*10^-05^** | 0.07 | 0.03 | 0.06^*^ | 0.15 | 2.16*10^-06^ | **4.63*10^-06^** | -0.01 | 0.92 | 0.92 |
| VLDL Small | 0.07 | 2.05*10^-04^ | **2.36*10^-04^** | 0.04 | 0.08 | 0.11 | 0.10 | 9.99*10^-05^ | **1.25*10^-04^** | 0.01 | 0.85 | 0.91 |
| VLDL Total | 0.10 | 8.61*10^-05^ | **1.08*10^-04^** | 0.04 | 0.28 | 0.35 | 0.19 | 1.90*10^-09^ | **9.40*10^-09^** | -0.02 | 0.61 | 0.78 |
| IDL Total | 0.22 | 8.11*10^-04^ | **8.69*10^-04^** | -0.005 | 0.95 | 0.95 | 0.18 | 0.06 | 0.06 | 0.37 | 2.10*10^-03^ | **0.02** |
| LDL Large | 18.20 | <1*10^-10^ | **<1*10^-10^** | 10.49 | 3.74*10^-03^ | **0.01** | 15.80 | 1.4*10^-05^ | **2.04*10^-05^** | 3.18 | 0.54 | 0.78 |
| LDL Small | 22.01 | <1*10^-10^ | **1.48*10^-10^** | 7.19 | 0.12 | 0.16 | 23.94 | 1.5*10^-06^ | **4.41*10^-06^** | 4.89 | 0.53 | 0.78 |
| LDL Total | 0.35 | 2.60*10^-09^ | **6.00*10^-09^** | 0.25 | 3.04*10^-03^ | **0.01** | 0.35 | 9.5*10^-06^ | **1.58*10^-05^** | 0.11 | 0.33 | 0.78 |
| HDL Large | 0.07 | <1*10^-10^ | **<1*10^-10^** | 0.03 | 1.19*10^-03^ | **8.76*10^-03^** | 0.05 | <1*10^-10^ | **1.30*10^-09^** | 0.02 | 0.09 | 0.26 |
| HDL Medium | 0.06 | 6.87*10^-05^ | **9.37*10^-05^** | 0.11 | 2.04*10^-05^ | **3.10*10^-04^** | 0.02 | 0.33 | 0.33 | 0.06 | 0.06 | 0.22 |
| HDL Small | 0.52 | 3.10*10^-09^ | **7.00*10^-09^** | 0.33 | 1.75*10^-03^ | **8.76*10^-03^** | 0.51 | 3.4*10^-07^ | **1.27*10^-06^** | 0.53 | 9.05*10^-04^ | **0.01** |
| HDL Total | 0.24 | 4.76*10^-03^ | **4.76*10^-03^** | 0.07 | 0.59 | 0.68 | 0.26 | 0.02 | **0.02** | 0.39 | 0.03 | 0.16^*^ |
| VLDL diameter | 0.06 | 6.22*10^-07^ | **1.17*10^-06^** | 0.00 | 0.90 | 0.95 | 0.07 | 2*10^-06^ | **4.63*10^-06^** | 0.02 | 0.43 | 0.78 |
| LDL diameter | 0.04 | 2.00*10^-10^ | **7.03*10^-10^** | 0.02 | 0.04 | 0.08^*^ | 0.06 | 2*10^-10^ | **1.30*10^-09^** | 0.01 | 0.63 | 0.78 |
| HDL diameter | 0.04 | <1*10^-10^ | **<1*10^-10^** | 0.01 | 0.06 | 0.10 | 0.03 | 2.5*10^-06^ | **4.70*10^-06^** | 0.01 | 0.58 | 0.78 |

**Significant results in bold**; ^*^nominally significant results with asterisk

**S 9 Table** Secondary Analyses - associations of GRSs and lipoprotein phenotypes stratified by gender

| Lipoprotein subfraction | | European-Americans | | | African-Americans | | | Hispanic-Americans | | | Chinese-Americans | | |
| --- | --- | --- | --- | --- | --- | --- | --- | --- | --- | --- | --- | --- | --- |
|  |  | **β** | ***P*** | **Q** | **β** | ***P*** | **Q** | **β** | ***P*** | **Q** | **β** | ***P*** | **Q** |
| VLDL Large | F^a^ | 0.11 | 4.11*10^-05^ | **6.85*10^-05^** | 0.04 | 0.19 | 0.27 | 0.09 | 3.62*10^-03^ | **9.04*10^-03^** | -0.04 | 0.50 | 0.97 |
|  | M^b^ | 0.10 | 5.17*10^-04^ | **7.75*10^-04^** | 0.07 | 0.07 | 0.12 | 0.10 | 3.24*10^-03^ | **6.08*10^-03^** | -3.32*10^-3^ | 0.95 | 0.95 |
| P for interaction | | 0.87 | |  | 0.61 | |  | 0.57 | |  | 0.80 | |  |
| VLDL Medium | F | 0.11 | 4.35*10^-04^ | **6.53*10^-04^** | 0.07 | 0.06 | 0.16 | 0.18 | 2.34*10^-06^ | **1.75*10^-05^** | 0.02 | 0.74 | 0.97 |
|  | M | 0.16 | 2.16*10^-06^ | **8.08*10^-06^** | 0.12 | 4.32*10^-3^ | **0.02** | 0.16 | 1.07*10^-04^ | **5.34*10^-04^** | 0.02 | 0.82 | 0.91 |
| P for interaction | | 0.26 | |  | 0.31 | |  | 0.94 | |  | 0.86 | |  |
| VLDL Small | F | 0.11 | 1.65*10^-05^ | **3.10*10^-05^** | 0.04 | 0.22 | 0.27 | 0.14 | 3.03*10^-05^ | **1.51*10^-04^** | 0.03 | 0.51 | 0.97 |
|  | M | 0.02 | 0.34 | 0.34 | 0.06 | 0.06 | 0.12 | 0.12 | 5.44*10^-04^ | **1.17*10^-03^** | -0.01 | 0.85 | 0.91 |
| P for interaction | | 0.03^*^ | |  | 0.44 | |  | 0.39 | |  | 0.47 | |  |
| VLDL Total | F | 0.10 | 2.27*10^-03^ | **2.61*10^-03^** | 0.06 | 0.11 | 0.24 | 0.20 | 8.51*10^-07^ | **1.28*10^-05^** | 6.43*10^-3^ | 0.91 | 0.97 |
|  | M | 0.12 | 2.40*10^-04^ | **4.50*10^-04^** | 0.05 | 0.30 | 0.34 | 0.18 | 2.31*10^-05^ | **1.73*10^-04^** | -0.02 | 0.74 | 0.91 |
| P for interaction | | 0.60 | |  | 0.62 | |  | 0.90 | |  | 0.61 | |  |
| IDL Total | F | 0.15 | 0.07 | 0.07 | -0.13 | 0.19 | 0.27 | 0.05 | 0.70 | 0.75 | 0.30 | 0.06 | 0.48 |
|  | M | 0.28 | 1.13*10^-03^ | **1.41*10^-03^** | 0.17 | 0.09 | 0.13 | -0.04 | 0.73 | 0.74 | 0.19 | 0.26 | 0.91 |
| P for interaction | | 0.42 | |  | 0.03^*^ | |  | 0.97 | |  | 0.57 | |  |
| LDL Large | F | 20.54 | 8.00*10^-10^ | **3.10*10^-09^** | 12.72 | 4.34*10^-3^ | **0.02** | 11.70 | 0.01 | **0.02** | -4.01 | 0.54 | 0.97 |
|  | M | 12.47 | 1.10*10^-04^ | **2.76*10^-04^** | 7.23 | 0.12 | 0.14 | 13.71 | 3.84*10^-03^ | **6.40*10^-03^** | 9.59 | 0.09 | 0.47 |
| P for interaction | | 0.08 | |  | 0.34 | |  | 0.89 | |  | 0.14 | |  |
| LDL Small | F | 19.92 | 1.59*10^-06^ | **4.76*10^-06^** | 5.39 | 0.30 | 0.30 | 11.62 | 0.08 | 0.13 | 7.79 | 0.45 | 0.97 |
|  | M | 20.34 | 1.97*10^-06^ | **8.08*10^-06^** | 10.55 | 0.08 | 0.13 | 16.14 | 5.41*10^-03^ | **7.44*10^-03^** | -9.55 | 0.36 | 0.91 |
| P for interaction | | 0.86 | |  | 0.49 | |  | 0.61 | |  | 0.19 | |  |
| LDL Total | F | 0.25 | 5.52*10^-04^ | **7.53*10^-04^** | 0.12 | 0.21 | 0.27 | 0.25 | 0.02 | **0.03** | 0.06 | 0.67 | 0.97 |
|  | M | 0.28 | 1.57*10^-04^ | **3.36*10^-04^** | 0.29 | 0.01 | **0.03** | 0.27 | 5.45*10^-03^ | **7.44*10^-03^** | -0.07 | 0.67 | 0.91 |
| P for interaction | | 0.80 | |  | 0.21 | |  | 0.53 | |  | 0.49 | |  |
| HDL Large | F | 0.08 | <1*10^-10^ | **<1*10^-10^** | 0.03 | 0.01 | **0.046** | 0.01 | 0.24 | 0.33 | 0.04 | 0.04 | 0.48 |
|  | M | 0.05 | <1*10^-10^ | **<1*10^-10^** | 0.04 | 3.76*10^-3^ | **0.02** | 0.01 | 0.16 | 0.21 | -0.03 | 0.09 | 0.47 |
| P for interaction | | 0.05 | |  | 0.84 | |  | 0.81 | |  | 0.01^*^ | |  |
| HDL Medium | F | 0.06 | 1.56*10^-03^ | **1.95*10^-03^** | 0.14 | 1.02*10^-5^ | **1.54*10^-4^** | 0.02 | 0.54 | 0.68 | -6.44*10^-3^ | 0.88 | 0.97 |
|  | M | 0.05 | 0.01 | **0.01** | 0.06 | 0.06 | 0.12 | 7.80*10^-3^ | 0.74 | 0.74 | -0.01 | 0.72 | 0.91 |
| P for interaction | | 0.65 | |  | 0.08 | |  | 0.96 | |  | 0.87 | |  |
| HDL Small | F | 0.76 | 4.00*10^-10^ | **2.00*10^-09^** | 0.40 | 4.33*10^-3^ | **0.02** | 0.56 | 1.10*10^-04^ | **4.14*10^-04^** | -0.16 | 0.47 | 0.97 |
|  | M | 0.35 | 9.14*10^-04^ | **1.25*10^-03^** | 0.44 | 6.97*10^-4^ | **0.01** | 0.51 | 7.39*10^-06^ | **1.11*10^-04^** | -0.11 | 0.55 | 0.91 |
| P for interaction | | 0.01^*^ | |  | 0.87 | |  | 0.76 | |  | 0.93 | |  |
| HDL Total | F | 0.33 | 3.81*10^-03^ | **4.08*10^-03^** | 0.38 | 0.02 | **0.046** | 0.07 | 0.60 | 0.70 | -0.01 | 0.97 | 0.97 |
|  | M | 0.18 | 0.07 | 0.07 | -0.10 | 0.49 | 0.52 | 0.44 | 3.36*10^-04^ | **8.93*10^-04^** | 0.13 | 0.55 | 0.91 |
| P for interaction | | 0.38 | |  | 0.04^*^ | |  | 0.21 | |  | 0.70 | |  |
| VLDL diameter | F | 0.07 | 1.14*10^-05^ | **2.43*10^-05^** | 0.02 | 0.25 | 0.27 | 0.06 | 2.34*10^-03^ | **7.02*10^-03^** | 0.02 | 0.45 | 0.97 |
|  | M | 0.06 | 3.49*10^-04^ | **5.81*10^-04^** | -0.01 | 0.55 | 0.55 | 0.08 | 1.69*10^-04^ | **6.33*10^-04^** | 0.01 | 0.65 | 0.91 |
| P for interaction | | 0.57 | |  | 0.19 | |  | 0.36 | |  | 0.68 | |  |
| LDL diameter | F | 0.04 | 3.07*10^-06^ | **7.68*10^-06^** | 0.01 | 0.24 | 0.27 | 0.02 | 0.17 | 0.26 | 1.12*10^-3^ | 0.94 | 0.97 |
|  | M | 0.04 | 4.88*10^-06^ | **1.47*10^-05^** | 0.02 | 0.02 | **0.047** | 0.04 | 3.57*10^-04^ | **8.93*10^-04^** | -4.79*10^-3^ | 0.75 | 0.91 |
| P for interaction | | 0.98 | |  | 0.48 | |  | 0.33 | |  | 0.69 | |  |
| HDL diameter | F | 0.05 | <1*10^-10^ | **<1*10^-10^** | 0.01 | 0.20 | 0.27 | -1.92*10^-4^ | 0.98 | 0.98 | 3.56*10^-3^ | 0.75 | 0.97 |
|  | M | 0.04 | 2.70*10^-09^ | **2.00*10^-08^** | 0.02 | 0.09 | 0.13 | 5.84*10^-3^ | 0.46 | 0.53 | -0.03 | 0.02 | 0.24^*^ |
| P for interaction | | 0.04^*^ | |  | 0.89 | |  | 0.44 | |  | 0.04^*^ | |  |

^a^ F: female; ^b^ M: male

**Significant results in bold**; ^*^nominally significant results with asterisk

**S 10 Table** Imputation quality metric for 61 unique lipoprotein associated SNPs in MESA

| **rsid** | **chr** | **position** | **observed / expected variance** | | | |
| --- | --- | --- | --- | --- | --- | --- |
|  |  |  | **EA** | **AA** | **HIS** | **CHN** |
| rs11591147 | 1 | 55505647 | 0.5 | 0.4 | 0.5 | 0.1 |
| rs1167998 | 1 | 62931632 | 1.0 | 1.0 | 1.0 | 1.1 |
| rs646776 | 1 | 109818530 | 1.0 | 1.0 | 1.1 | 1.0 |
| rs6754295 | 2 | 21206183 | 1.0 | 1.0 | 1.0 | 1.0 |
| rs676210 | 2 | 21231524 | 1.0 | 1.0 | 1.0 | 1.0 |
| rs1713222 | 2 | 21271323 | 1.0 | 0.9 | 1.0 | 0.7 |
| rs312985 | 2 | 21378805 | 1.0 | 1.0 | 1.0 | 1.0 |
| rs1260326 | 2 | 27730940 | 1.0 | 1.0 | 1.0 | 0.9 |
| rs4704810 | 5 | 156273481 | 0.8 | 0.8 | 0.9 | 0.5 |
| rs6873053 | 5 | 156376703 | 0.8 | 0.8 | 0.7 | 0.4 |
| rs1363232 | 5 | 156383422 | 1.0 | 1.0 | 1.1 | 1.0 |
| rs2240466 | 7 | 72856269 | 1.0 | 0.9 | 1.0 | 0.9 |
| rs983309 | 8 | 9177732 | 1.0 | 1.0 | 1.0 | 0.8 |
| rs328 | 8 | 19819724 | 1.0 | 1.0 | 1.0 | 1.0 |
| rs331 | 8 | 19820405 | 1.0 | 1.0 | 1.0 | 1.0 |
| rs1059611 | 8 | 19824563 | 1.0 | 1.0 | 1.0 | 1.0 |
| rs2083637 | 8 | 19865175 | 1.0 | 1.0 | 1.0 | 1.0 |
| rs6982636 | 8 | 126479315 | 1.0 | 1.0 | 1.0 | 0.9 |
| rs507666 | 9 | 136149399 | 1.0 | 1.0 | 1.0 | 1.0 |
| rs7923609 | 10 | 65133822 | 1.0 | 1.0 | 1.1 | 1.0 |
| rs7938647 | 11 | 10061423 | 1.0 | 1.0 | 1.1 | 0.8 |
| rs174537 | 11 | 61552680 | 1.0 | 1.0 | 1.1 | 1.1 |
| rs174546 | 11 | 61569830 | 1.0 | 1.0 | 1.1 | 1.1 |
| rs1535 | 11 | 61597972 | 1.0 | 0.9 | 1.1 | 1.1 |
| rs3135506 | 11 | 116662407 | 0.9 | 0.8 | 1.0 | NA* |
| rs518181 | 11 | 116772787 | 1.0 | 1.0 | 1.0 | 1.0 |
| rs7307277 | 12 | 124475156 | 1.0 | 1.0 | 1.0 | 1.0 |
| rs1532085 | 15 | 58683366 | 0.9 | 1.0 | 1.0 | 1.0 |
| rs1800588 | 15 | 58723675 | 1.0 | 0.9 | 1.0 | 0.8 |
| rs1800775 | 16 | 56995236 | 0.9 | 0.9 | 0.9 | 0.9 |
| rs1864163 | 16 | 56997233 | 0.8 | 0.7 | 0.8 | 0.8 |
| rs7499892 | 16 | 57006590 | 0.7 | 0.7 | 0.8 | 0.8 |
| rs4986970 | 16 | 67976320 | 0.6 | 0.6 | 0.6 | 0.1 |
| rs1109166 | 16 | 67977382 | 1.0 | 1.0 | 1.1 | 1.0 |
| rs7200210 | 16 | 67981896 | 1.0 | 1.0 | 1.1 | 0.8 |
| rs255049 | 16 | 68013471 | 1.0 | 1.0 | 1.1 | 1.0 |
| rs6511720 | 19 | 11202306 | 0.6 | 0.5 | 0.6 | 0.2 |
| rs405509 | 19 | 45408836 | 0.8 | 0.8 | 0.8 | 0.8 |
| rs769449 | 19 | 45410002 | 0.8 | 0.6 | 0.7 | 0.7 |
| rs439401 | 19 | 45414451 | 0.6 | 0.6 | 0.7 | 0.7 |
| rs6102085 | 20 | 39281629 | 1.0 | 1.0 | 1.0 | 1.0 |
| rs2865892 | 20 | 39403896 | 1.0 | 1.0 | 1.0 | 1.0 |
| rs6065904 | 20 | 44534651 | 0.9 | 0.8 | 1.0 | 0.8 |
| rs4810479 | 20 | 44545048 | 0.9 | 0.9 | 1.0 | 0.9 |
| rs6065906 | 20 | 44554015 | 1.0 | 0.9 | 0.9 | 1.1 |
| *: NA because expected variance is zero while observed variance is 0.000006 | | | | | |  |
